# Oncogenic EGFR rewires STING-TBK1 signalosomes to license DNA damage tolerance in NSCLC

**DOI:** 10.1038/s44318-026-00856-3

**Published:** 2026-07-10

**Authors:** Qin Shen, Chen Mei, Yidan Chen, Qian Zhang, Qingzhe Wu, Xinyuan Yu, Fei Zhang, Shengduo Liu, Chen Chen, Cunqi Ye, Qi Zhang, Xin-Hua Feng, Li Shen, Hai Song, Tingbo Liang, Penghong Song, Bing Xia, Pinglong Xu

**Affiliations:** 1 MOE Laboratory of Biosystems Homeostasis and Protection, Zhejiang Provincial Key Laboratory for Cancer Molecular Cell Biology, Life Sciences Institute, Zhejiang University, Hangzhou, China; 2 Institute of Intelligent Medicine, ZJU-Hangzhou Global Scientific and Technological Innovation Center, Hangzhou, China; 3 Department of Hepatobiliary and Pancreatic Surgery and Zhejiang Provincial Key Laboratory of Pancreatic Disease, The First Affiliated Hospital, University School of Medicine, Zhejiang University, Hangzhou, China; 4 Department of Thoracic Cancer, Affiliated Hangzhou Cancer Hospital, Westlake University, Hangzhou, China; 5 Cancer Center, Zhejiang University, Hangzhou, China

**Keywords:** Cancer, DNA Replication, Recombination & Repair, Immunology

## Abstract

EGFR hotspot mutations (mEGFR), including primary L858R, exon 19 deletion, and secondary T790M, are pivotal oncogenic drivers in human non-small cell lung cancer (NSCLC). At the same time, NSCLC resistance to third-generation tyrosine kinase inhibitors (TKIs) is a major clinical challenge and remains mechanistically unresolved. Here, we uncover a previously unrecognized tumor cell-intrinsic mechanism in which mutant EGFR (mEGFR) exploits innate immune signaling via the cGAS–STING–TBK1 pathway to sustain oncogenic signaling and therapeutic resistance. Mechanistically, mutant EGFR kinase aberrantly associates with STING signalosomes and phosphorylates STING (Y245/Y314) and TBK1 (Y577/Y677), stabilizing and hyperactivating TBK1 and establishing an unexpected kinase loop critical for DNA damage repair. Genetic or pharmacological disruption of mEGFR–STING–TBK1 coupling sensitizes resistant patient-derived NSCLC organoids to chemotherapy. Combining TBK1 inhibition with cisplatin suppressed mEGFR-driven tumors in murine models of spontaneous and immunocompetent NSCLC and in patient-derived organoids. Our findings suggest a new function of cGAS–STING in DNA damage tolerance, its paradoxical exploitation by oncogenic driver mutations, and an innate immune therapeutic vulnerability in NSCLC.

## Synopsis

Epidermal growth factor receptor (EGFR) hyperactivation drives lung cancer, but its mechanistic consequences remain unclear. Here, mutant EGFR is shown to engage innate immune signaling in non-small cell lung cancer (NSCLC), activating pro-survival pathways, and reinforcing DNA repair.

- EGFR hotspot mutations facilitate cGAS-STING signaling dependent on kinase activity in human NSCLC cells.
- Mutant EGFR aberrantly integrates into STING signalosomes.
- Tyrosine phosphorylation by mutant EGFR solidifies STING clustering and hyperactivates TBK1.
- STING and TBK1 are required for mutant EGFR-driven NSCLC tumorigenesis in vivo and confer resistance to cisplatin.
- Combining cisplatin and TBK1 inhibition diminishes mEGFR-driven spontaneous NSCLC tumors.

## Abstract (web summary)

A novel tumor-cell-intrinsic mechanism highlights hijacking of innate immune signaling by mutant EGFR, supporting pro-survival pathways and resistance in lung cancer.

## Graphical abstract

**Figure:**
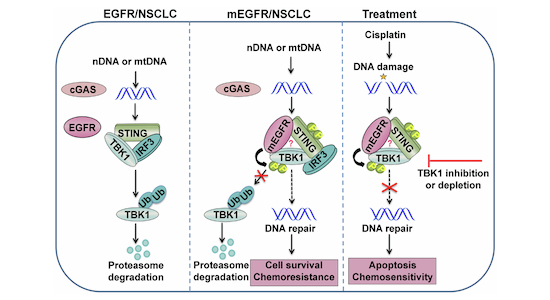

**Figure:**
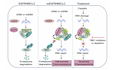

## Introduction

Hyperactivation, overexpression, or genetic alterations in epidermal growth factor receptor (EGFR) contribute to the development and progression of an array of cancers, including breast cancer, lung cancer, colorectal and esophageal cancers, head and neck cancers, and glioblastoma (Arteaga and Engelman, 2014; Blasco et al, 2019). Notably, hotspot mutations in the EGFR gene, especially the L858R mutation in exon 21 and exon 19 deletions, account for 85–90% of all EGFR mutations observed in non-small cell lung cancer (NSCLC) (Kosaka et al, 2004; Inukai et al, 2006). These mutations lead to constitutive activation of the EGFR tyrosine kinase (Lynch et al, 2004; Yun et al, 2008), sustaining persistent oncogenic signaling through Ras-ERK, PLCγ1, PI3K-Akt, and STAT pathways (Yarden, 2001; Yarden and Pines, 2012), thereby promoting cancer cell proliferation and survival while conferring a significant growth advantage. NSCLC driven by EGFR mutations initially responds to first- and second-generation TKIs, including erlotinib (Miyamoto et al, 2020), gefitinib (Makoto et al, 2010), and afatinib (Bian et al, 2023). However, resistance frequently arises through secondary mutations (Jia et al, 2016; Yoshida et al, 2014), particularly the T790M variant, and the activation of alternative signaling pathways (Rotow and Bivona, 2017). Third-generation EGFR TKIs, such as Osimertinib (Cross et al, 2014; Ramalingam et al, 2018), can overcome T790M-mediated resistance, but acquired resistance continues to emerge (Chmielecki et al, 2023), highlighting the urgent need to develop novel therapeutic strategies. These TKIs provide a lifeline for patients with specific resistance mutations, while immunotherapy and chemotherapy are standard subsequent treatment options when resistance develops (Planchard et al, 2023; Zhao et al, 2024). Cisplatin, a platinum-based chemotherapeutic agent that induces DNA damage and subsequent cell death (Siddik, 2003), has been used for decades to treat various solid tumors, including NSCLC (Kelland, 2007). However, the clinical efficacy of cisplatin is often compromised by the emergence of drug resistance (Galluzzi et al, 2012), occurring through numerous mechanisms, such as increased DNA repair, drug efflux, alterations in cell survival signals, epigenetic regulation, and evasion of apoptosis (Kelland, 2007; Shen et al, 2012). Therefore, elucidating the mechanisms underlying next-generation TKIs and chemotherapeutic agents is paramount for designing more effective therapeutic strategies.

Understanding the molecular mechanisms underlying mEGFR-driven NSCLC, including the interactions between mEGFR and innate immune signaling pathways, is crucial for developing treatment strategies to overcome TKI and chemotherapy resistance. cGAS–STING (cyclic GMP–AMP synthase-stimulator of interferon genes) innate immune signaling triggers an array of physiological and pathological processes, including cytokine production, autophagy, and senescence (Chen and Xu, 2023; Liu et al, 2026), and is critical in host defense (Roers et al, 2016; Wu et al, 2020), autoimmune diseases (An et al, 2017), neurodegenerative diseases (Freischmidt et al, 2015; Yu et al, 2020; Wang et al, 2024; Liu et al, 2024), organ fibrosis (Zhang et al, 2022; Chung et al, 2019; Wu et al, 2024), and antitumor immunity (Meng et al, 2021; Wu et al, 2019a; Zhou et al, 2024). Upon detecting mislocalized dsDNA, cGAS uses ATP and GTP to synthesize 2’3’-cGAMP, a second messenger that activates STING proteins. STING, binding with cGAMP, transitions from the endoplasmic reticulum to the Golgi apparatus, and this migration enables its interaction with TBK1 (TANK-binding kinase 1) and triggers the activation of IRF3 (Interferon regulatory factor 3) and NF-κB (Nuclear factor kappa-light-chain-enhancer of activated B cells). These transcription factors regulate the transcription of type I interferons and other immune response genes, which are critical for cellular defense mechanisms and homeostasis (Chen and Xu, 2023).

cGAS–STING signaling is traditionally considered a major initiator of antitumor immunity. The intratumoral administration of STING agonists induces robust immune-mediated tumor eradication in murine models, elucidating the therapeutic potential of targeted STING activation in both cancer cells and immune cells (Baird et al, 2016; Sen et al, 2019). However, accumulating evidence over the past decade has established that cGAS–STING can also exert tumor cell-intrinsic, context-dependent effects that support tumor progression and therapeutic resistance in certain cancers (Lv et al, 2023; Hong et al, 2022). Whether and how oncogenic receptor tyrosine kinases directly interface with this pathway remains incompletely understood. In this study, we define a mechanistic link between oncogenic EGFR and the cGAS–STING–TBK1 axis in EGFR-mutant NSCLC. We show that EGFR hotspot mutations engage STING–TBK1 through kinase-dependent association and tyrosine phosphorylation, stabilize activated TBK1 by limiting K48-linked ubiquitination, activate pro-survival pathways, and support DDR-associated responses and DNA damage tolerance in mutant cells. We restore chemosensitivity by targeting this aberrant EGFR-STING–TBK1 axis, unveiling a previously unrecognized therapeutic vulnerability and a novel framework for overcoming TKI and chemotherapy resistance in EGFR-mutant NSCLC.

## Results

### EGFR hotspot mutations kinase-dependently facilitate cGAS–STING signaling

**Figure EV1:**
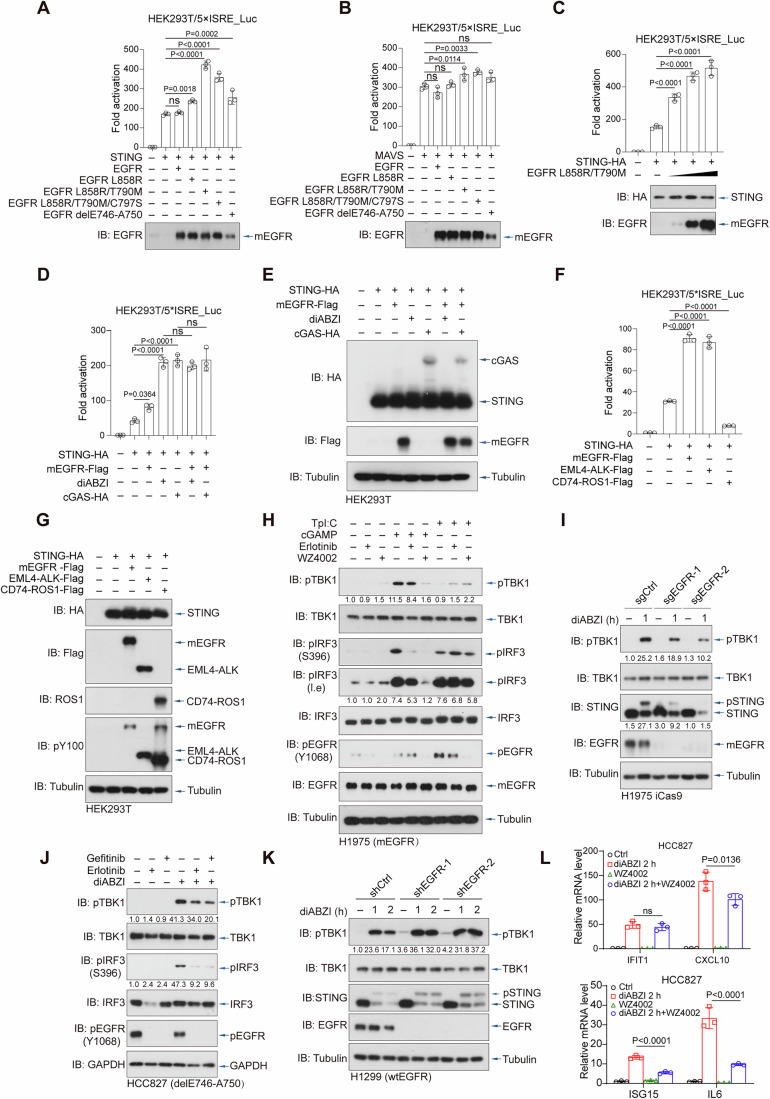
EGFR hotspot mutations kinase-dependently facilitate cGAS–STING signaling. (**A**) HEK293T cells were co-transfected with an IRF3-responsive ISRE-luciferase reporter, STING, and various EGFR constructs (L858R, L858R/T790M, L858R/T790M/C797S, or delE746-A750). Luciferase activity was measured 24 h post-transfection and presented as fold induction relative to the empty vector control. EGFR and mutant EGFR expression were confirmed by immunoblotting. (**B**) HEK293T ISRE-Luc cells were co-transfected with MAVS and the indicated EGFR mutants to evaluate their effect on MAVS-mediated IRF3 activation. Luciferase activity is shown as fold induction compared to the vector control. EGFR and mutant EGFR expression were validated by immunoblotting. (**C**) HEK293T ISRE-Luc cells were co-transfected with STING-HA and increasing amounts of EGFR L858R/T790M. Luciferase activity was quantified as a fold change relative to vector control. STING and EGFR expression were confirmed by immunoblotting. (**D**) HEK293T/5×ISRE-Luc cells were transfected with STING-HA alone or together with mEGFR-Flag, and were either left untreated or stimulated with diABZI, or co-transfected with cGAS-HA, as indicated. (**E**) Immunoblotting analysis of lysates from HEK293T cells transfected as in (**D**), confirming expression of STING-HA, mEGFR-Flag, and cGAS-HA. Tubulin is shown as a loading control. (**F**) HEK293T cells were co-transfected with an IRF3-responsive ISRE-luciferase reporter with STING-HA together with mEGFR-Flag (L858R/T790M), EML4-ALK-Flag, or CD74-ROS1-Flag, as indicated. Luciferase activity was measured 24 h post-transfection and is presented as fold activation relative to the vector control. (**G**) Immunoblotting analysis of lysates from HEK293T cells transfected as in (**F**) to confirm expression of STING-HA and the indicated oncogenic kinase constructs. pY100 was used as a readout of tyrosine phosphorylation. (**H**) H1975 NSCLC cells were treated with the STING agonist cGAMP (1 h), the dsRNA analog poly(I:C) (4 h), or vehicle, in the presence or absence of erlotinib or WZ4002. Phosphorylation and total levels of TBK1, IRF3, and EGFR were assessed by immunoblotting. (**I**) H1975 iCas9 cells transduced with control or EGFR-targeting sgRNAs were treated with diABZI (1 h) and subjected to immunoblotting. (**J**) HCC827 cells (EGFR delE746-A750) were treated with diABZI (1 h) with or without erlotinib or gefitinib (10 μM) and analyzed by immunoblotting as indicated. (**K**) H1299 (wtEGFR) cells expressing control (shCtrl) or EGFR-targeting shRNAs (shEGFR-1, shEGFR-2) were treated with diABZI for the indicated time points and analyzed by immunoblotting as indicated. (**L**) HCC827 cells were treated with diABZI (2 h), WZ4002, or the combination. mRNA levels of *ISG15*, *IL6, CXCL10*, and *IFIT1* were measured by quantitative RT-PCR. Data are presented as mean ± SEM. Statistical significance was determined by one-way ANOVA with Tukey’s multiple comparisons test. Exact *P* values are indicated in the panel. Data in (**A**–**D**, **F**) are presented as mean ± SEM, and statistical significance was determined by one-way ANOVA with Dunnett’s multiple comparisons test. Exact *P* values are indicated in the respective panels, ns, no significance. Unless otherwise indicated, *n*  =  3 independent biological experiments. Densitometric values are indicated below the bands and provided in the source data. Source data are available online for this figure.

EGFR attracts considerable attention due to its hyperactivation in various cancer pathologies, particularly in NSCLC (Da Cunha Santos et al, 2011). The roles of NSCLC-related EGFR hotspot mutations in cGAS–STING signaling were initially investigated using reconstitution in HEK293T cells. Intriguingly, we found that NSCLC-related EGFR hotspot mutants enhanced, rather than suppressed, IRF3 transactivation induced by STING but not MAVS (mitochondrial antiviral signaling protein) (Fig. EV1A,B). The EGFR L858R/T790M mutation, the most widely distributed mEGFR mutation in NSCLC targeted therapy, potently amplified STING signaling in a dose-dependent manner (Fig. EV1C). To benchmark the magnitude of the mEGFR effect, we compared STING + mEGFR with canonical STING activation conditions, including diABZI treatment and cGAS co-expression. mEGFR reproducibly enhanced STING-dependent reporter activity relative to STING alone, but did not produce activation comparable to canonical diABZI- or cGAS-driven stimulation. Moreover, when STING signaling was already strongly activated by diABZI or cGAS, mEGFR did not further increase reporter output substantially (Fig. EV1D,E). Extending this reporter analysis to additional oncogenic drivers, such as co-expression of EML4-ALK, also potentiated STING-dependent ISRE-luc activity, though CD74-ROS1 did not show a positive response (Fig. EV1F,G), suggesting a possible tyrosine phosphorylation-mediated, selective regulation of cGAS–STING signaling.

**Figure 1:**
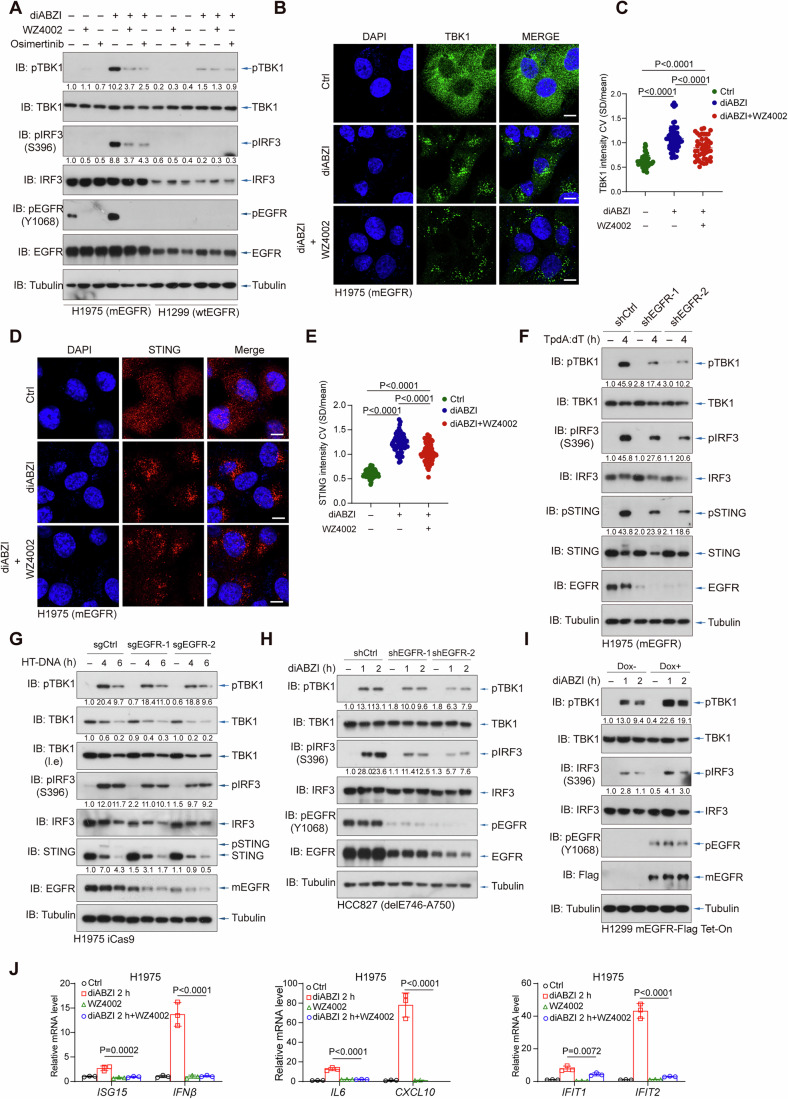
EGFR hotspot mutations kinase-dependently facilitate cGAS–STING signaling. (**A**) H1975 (EGFR L858R/T790M) and H1299 (wild-type EGFR) cells were treated with the STING agonist diABZI (10 μM, 1 h), with or without third-generation EGFR inhibitors WZ4002 or osimertinib (10 μM, 6 h). Immunoblotting was performed to assess the phosphorylation and total protein levels of TBK1, IRF3, and EGFR. (**B**) Immunofluorescence staining of TBK1 (green) in H1975 cells following diABZI stimulation (1 h) with or without WZ4002 (6 h), revealing subcellular clustering. Scale bars, 10 μm. (**C**) Quantification of TBK1 spatial heterogeneity, expressed as the coefficient of variation (CV = SD/mean) of per-cell fluorescence intensity, to assess diABZI-induced punctate redistribution and its attenuation by WZ4002. Each dot represents one cell. Data were pooled from three independent experiments (Ctrl *n* = 55, diABZI *n* = 58, diABZI + WZ4002 *n* = 45 cells). (**D**) Immunofluorescence staining of STING (red) was performed as in (**B**). Scale bars, 10 μm. (**E**) Quantification of STING spatial heterogeneity was performed as in (**C**). Data were pooled from three independent experiments (Ctrl *n* = 66, diABZI *n* = 65, diABZI + WZ4002 *n* = 76 cells). (**F**) H1975 cells expressing control or EGFR-targeting shRNAs were stimulated with poly(dA:dT) for 4 h, followed by immunoblotting analysis. (**G**) H1975 iCas9 cells transduced with control or EGFR-targeting sgRNAs were stimulated with herring testes DNA (HT-DNA) for the indicated time points and subjected to immunoblotting. (**H**) HCC827 cells with EGFR knockdown were treated with diABZI for the indicated time points, and phosphorylation of TBK1 and IRF3 was analyzed. (**I**) H1299 EGFR L858R/T790M Tet-On cells were treated with or without doxycycline (Dox) for 48 h, followed by diABZI stimulation for 1 or 2 h. TBK1 and IRF3 activation was evaluated by immunoblotting. (**J**) H1975 cells were treated with diABZI (2 h), WZ4002 (6 h), or both. mRNA levels of the interferon-stimulated genes *ISG15, IFNβ, IL6*, *CXCL10*, *IFIT1*, and *IFIT2* were measured by quantitative RT-PCR. Data are presented as mean ± SEM (*n* = 3). Statistical significance in (**C**, **E**, **J**) was determined by one-way ANOVA with Tukey’s multiple comparisons test. Exact *P* values are indicated in the respective panels. Unless otherwise indicated, *n*  =  3 independent biological experiments. Densitometric values are indicated below the bands and provided in the source data. Source data are available online for this figure.

In agreement with these observations, STING–TBK1–IRF3 signaling triggered by the STING agonist diABZI was attenuated when mEGFR activity was abrogated by the third-generation tyrosine kinase inhibitor WZ4002 and osimertinib in mEGFR-driven NSCLC cells H1975 (with L858R/T790M mutation), as evidenced by compromised levels of phospho-TBK1 and IRF3 (Fig. 1A). Conversely, H1299 NSCLC cells (with wild-type EGFR) were unresponsive to EGFR inhibitors and failed to influence STING signaling upon EGFR inhibition (Fig. 1A). STING signaling stimulated by its natural agonist 2’3’-cGAMP, rather than dsRNA sensing MAVS signaling, was impeded by mEGFR inhibitors (Fig. EV1H). Given that STING and TBK1 clustering are key indicators of STING signaling activation, we measured these events in mEGFR-driven H1975 cells. Confocal imaging revealed that diABZI induced prominent punctate structures of TBK1 (Fig. 1B) and STING (Fig. 1D) in H1975 cells, which were attenuated by WZ4002 co-treatment. We measured single-cell spatial heterogeneity (CV = SD/mean) of TBK1/STING fluorescence within cytoplasmic ROIs to quantify these changes. As expected for punctate aggregation, diABZI increased while WZ4002 significantly reduced CV (Fig. 1C,E). Consistent with these observations, depleting mEGFR using distinct shRNAs similarly curbed cGAS–STING signaling activated by synthetic B-DNA analogs in H1975 (Fig. 1F). We further validated this observation by CRISPR/Cas9-mediated deletion of endogenous EGFR in H1975 iCas9 cells, which impaired HT-DNA- and diABZI-induced pathway activation (Figs. 1G and EV1I).

The effect of mEGFR inhibition on STING signaling was extended to other NSCLCs with EGFR mutations, including HCC827 cells harboring an EGFR delE746-A750 mutation. Both EGFR depletion and inhibition similarly attenuated diABZI-induced STING pathway activation, as reflected by reduced pTBK1 and pIRF3 (Figs. 1H and EV1J). By contrast, in H1299 cells expressing wtEGFR, diABZI-induced activation of STING and TBK1 was largely preserved despite EGFR depletion (Fig. EV1K). Consistently, ectopic expression of mEGFR in H1299 cells significantly enhanced diABZI-induced STING signaling (Fig. 1I). We also observed that mEGFR inhibitor WZ4002 impeded mRNA levels of IFNβ and ISGs in diABZI-stimulated H1975 (Fig. 1J), as well as ISGs and inflammatory transcripts in HCC827 cells (Fig. EV1L). These observations indicate that oncogenic EGFR kinase activity enhances STING signaling activation and transcriptional branch in EGFR-mutant NSCLC cells.

### mEGFR associates with STING signalosomes

**Figure 2:**
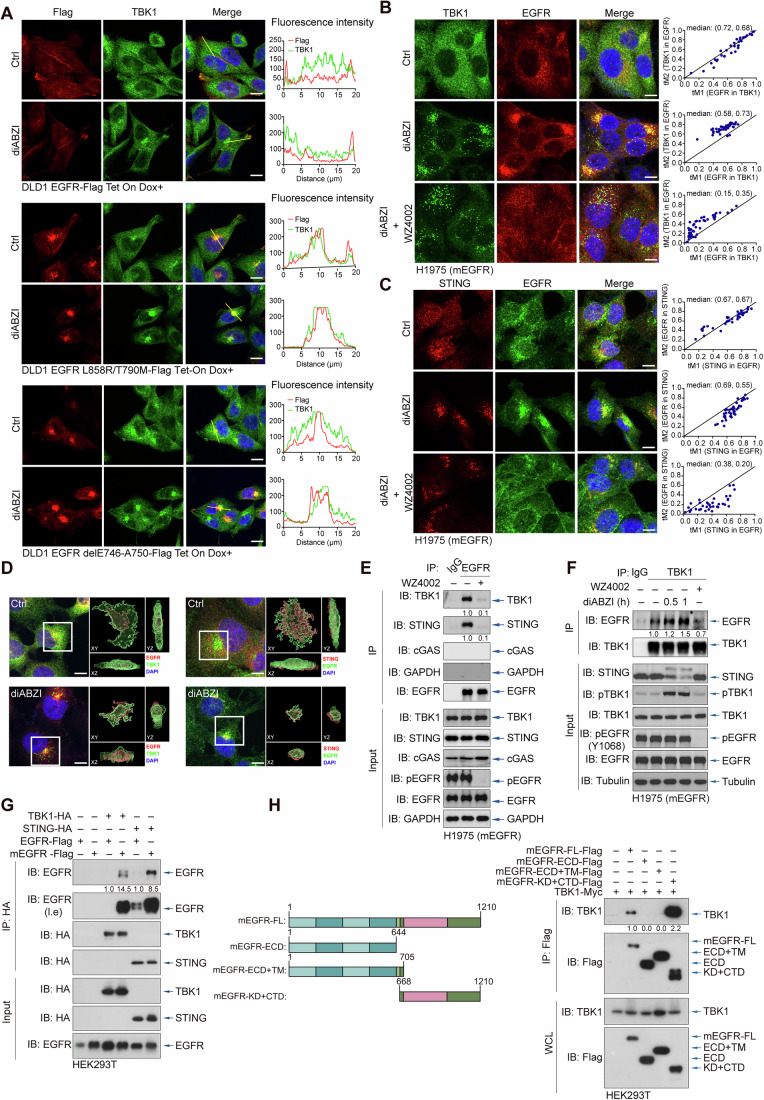
mEGFR, but not wild-type EGFR, associates with STING signalosomes. (**A**) DLD1 Tet-On cells stably expressing Flag-tagged wild-type EGFR, EGFR L858R/T790M, or EGFR delE746-A750 were induced with doxycycline (Dox) and treated with diABZI for 1 h or left untreated (Ctrl). Cells were fixed and stained for Flag (red) and TBK1 (green), and nuclei were counterstained with DAPI (blue). Representative immunofluorescence images show the subcellular localization of EGFR variants with TBK1, with the two proteins occupying similar intracellular areas, suggesting potential partial co-localization. Scale bars, 10 μm. (**B**, **C**) H1975 cells were treated with diABZI with or without WZ4002 and stained with antibodies against endogenous EGFR and TBK1 (**B**), or EGFR and STING (**C**). Scale bars, 10 μm. (**D**) Representative 3D reconstructions of confocal z-stacks from H1975 cells under Ctrl or diABZI treatment, showing the spatial organization of EGFR clusters with TBK1 (left) or STING (right). Boxed regions in the merged images indicate the volumes used for reconstruction. Orthogonal views (XY, XZ, YZ) are shown for each reconstruction; nuclei are counterstained with DAPI. Scale bars, 10 μm. (**E**) H1975 cells were treated with WZ4002 (10 μM, 6 h) or vehicle, as indicated. Cell lysates were immunoprecipitated using anti-EGFR or isotype-matched IgG control antibodies and analyzed by immunoblotting for TBK1, STING, cGAS, GAPDH, and EGFR. cGAS and GAPDH served as unrelated negative proteins for co-immunoprecipitation specificity. (**F**) H1975 cells were treated with diABZI (0.5 or 1 h) or WZ4002 (10 μM, 6 h). Lysates were immunoprecipitated with anti-TBK1 or IgG control, and analyzed for EGFR association by immunoblotting. (**G**) HEK293T cells were transfected with Flag-tagged wild-type EGFR or EGFR L858R/T790M (hereafter abbreviated as mEGFR), HA-tagged TBK1 or STING, or combinations. After 24 h, lysates were immunoprecipitated with anti-HA antibody and analyzed by immunoblotting with anti-EGFR and anti-HA antibodies. The corresponding HA-bait-negative conditions are shown. (**H**) Left: Schematic of EGFR truncation constructs used for interaction mapping, including full-length (FL), extracellular domain only (ECD), ECD plus transmembrane domain (ECD + TM), and kinase domain with C-terminal tail (KD + CTD). Right: HEK293T cells were co-transfected with Flag-tagged mEGFR truncation constructs and Myc-tagged TBK1. Anti-Flag immunoprecipitates were analyzed by immunoblotting for Flag and TBK1. The corresponding Flag-bait-negative condition is shown. Data information: unless otherwise indicated, *n*  =  3 independent biological experiments. Densitometric values are indicated below the bands and provided in the source data. Source data are available online for this figure.

**Figure EV2:**
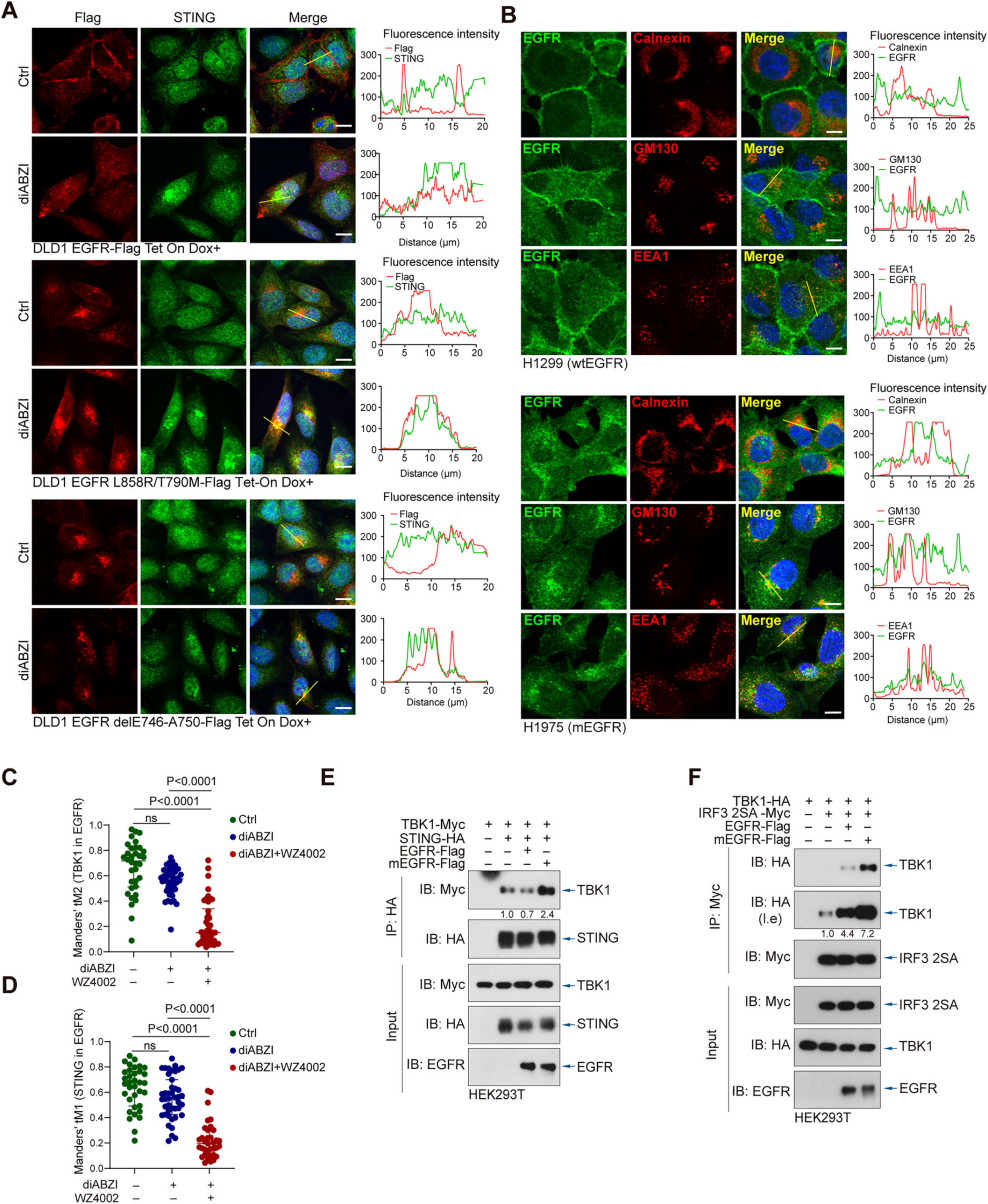
Mutant and wild-type EGFR differentially localize and interact with TBK1–STING signalosomes. (**A**) DLD1 cells expressing the indicated EGFR variants were treated with diABZI (1 h) with or without WZ4002 (10 μM, 6 h), then fixed and stained with antibodies against Flag (red) and STING (green). Right, fluorescence intensity profiles along the indicated line scans illustrate co-enrichment of Flag and STING signals in clustered regions. Scale bars, 10 μm. (**B**) H1975 and H1299 cells were fixed and co-stained with antibodies against EGFR (green) and one of the following organelle markers, including calnexin (ER), GM130 (Golgi), or EEA1 (early endosomes) in red. Representative confocal images are shown. Right, fluorescence intensity profiles along the indicated line scans (yellow) in the merged images illustrate the degree of spatial overlap between EGFR and each organelle marker. Scale bars, 10 μm. (**C**, **D**) Quantification of spatial overlap in H1975 (mEGFR) cells treated with diABZI with or without WZ4002, based on Manders overlap coefficients measured from single-cell ROIs. (**C**) Manders’ tM2 (TBK1 in EGFR). (**D**) Manders’ tM1 (STING in EGFR). Each dot represents one cell. Data were from three independent experiments (total cell numbers: TBK1-EGFR, Ctrl *n* = 34, diABZI *n* = 43, diABZI + WZ4002 *n* = 46; STING-EGFR, Ctrl *n* = 35, diABZI *n* = 43, diABZI + WZ4002 *n* = 35). Data in (**C**, **D**) are presented as mean ± SEM and statistical significance was determined by the Kruskal–Wallis test with Dunn’s multiple comparisons. Exact *P* values are indicated in the respective panels; ns, no significance. (**E**) HEK293T cells were co-transfected with Flag-tagged wild-type EGFR or mEGFR, HA-tagged STING, and Myc-tagged TBK1. Cell lysates were immunoprecipitated with anti-HA and analyzed by immunoblotting with anti-Myc, anti-HA, and anti-EGFR antibodies. (**F**) HEK293T cells were co-transfected with Flag-tagged wild-type EGFR or mEGFR, Myc-tagged IRF3 2SA mutant (S385A/S386A), and HA-tagged TBK1. Myc immunoprecipitates were analyzed by immunoblotting. l.e., longer exposure. Data information: unless otherwise indicated, *n*  =  3 independent biological experiments. Densitometric values are indicated below the bands and provided in the source data. Source data are available online for this figure.

To investigate the mechanisms by which mEGFR potentiates STING signaling, we generated DLD1 cells with inducible expression of Flag-tagged EGFR wild-type, L858R/T790M, and delE746-A750. Both mEGFR variants showed spatial association with TBK1 under basal conditions, and following diABZI stimulation, were evidently associated with TBK1, as supported by merged images and line-scan intensity profiles (Fig. 2A). In contrast, WT EGFR displayed a largely membrane-associated distribution with minimal overlap with TBK1 puncta under the same conditions (Fig. 2A). Similar results were obtained for mEGFR-STING association, as diABZI promoted co-enrichment of mEGFR with STING-positive assemblies. In contrast, WT EGFR exhibited little spatial coincidence (Fig. EV2A).

We next examined endogenous proteins in H1975 cells. We found that even in the absence of an exogenous agonist, EGFR exhibited measurable spatial association with TBK1 and STING, suggesting their pre-existing association. Similarly, endogenous EGFR L858R/T790M proteins in H1975 cells exhibited co-enrichment with TBK1 and STING puncta following diABZI treatment, an interaction that was largely abolished by mEGFR inhibitor WZ4002 (Fig. 2B,C). Consistent with this, single-cell Manders analyses indicated that diABZI alone did not significantly increase overall overlap relative to basal conditions, whereas WZ4002 robustly decreased EGFR spatial association with TBK1 and STING (Figs. 2B,C and EV2C,D), indicating kinase-dependent maintenance of this association. We reconstructed confocal z-stacks in three dimensions (3D) to exclude possible artifacts in 2D projections. EGFR signaling was well associated with TBK1-STING assemblies across multiple z-planes, supporting their three-dimensional inclusion (Fig. 2D; Movie EV1). Immunofluorescence imaging similarly revealed differences in subcellular distribution for mEGFR and wtEGFR, as mEGFR localized partially at the Golgi apparatus and endosome, while wtEGFR mainly anchored to the plasma membrane (Fig. EV2B).

Coimmunoprecipitation experiments further validated these observations in mEGFR-expressing H1975 cells. In the endogenous EGFR IP experiment, STING and TBK1 were specifically detected under basal conditions. In contrast, the unrelated proteins cGAS and GAPDH were not, and WZ4002 reduced the association of EGFR with both STING and TBK1 (Fig. 2E). In a complementary endogenous TBK1 IP, EGFR association remained detectable at baseline, and it was modestly enhanced following diABZI stimulation (Fig. 2F). Together, these results support the notion that mEGFR engages STING–TBK1 assemblies through a kinase-dependent, STING agonist-improved association.

By contrast, wild-type EGFR interacted only weakly with STING and TBK1, even when ectopically expressed (Fig. 2G). Intriguingly, mEGFR, unlike wild-type EGFR, markedly enhanced the associations of TBK1 with both its upstream adapter STING and downstream effector IRF3, indicating an assisting role of mEGFR in STING signalosome assembly (Fig. EV2E,F). We next analyzed their association using protein truncation mutants to determine whether their interaction is membrane-trafficking or protein-domain based. Kinase- and C-terminal domains of mEGFR were sufficient to interact with TBK1 (Fig. 2H). These reconstitution-based findings consistently indicate that mEGFR, but not wtEGFR, is an integral component of classical STING–TBK1–IRF3 signalosomes.

### Tyrosine phosphorylation of STING and TBK1 by mEGFR solidifies STING clustering

**Figure 3:**
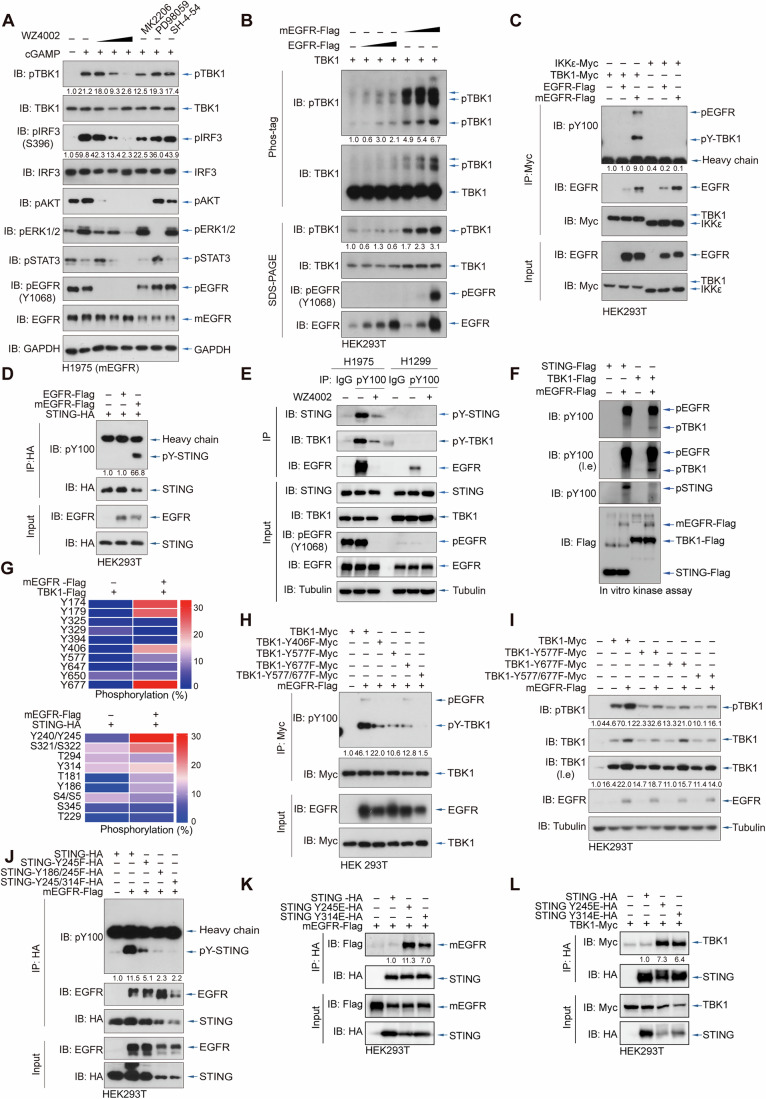
mEGFR tyrosine phosphorylates and facilitates STING and TBK1 activation. (**A**) H1975 cells were pretreated with increasing concentrations of EGFR inhibitor WZ4002 (0.1, 1, or 10 μM) or with specific pathway inhibitors MK2206 (AKT inhibitor), PD98059 (MEK1 inhibitor), or SH-4-54 (STAT3 inhibitor), each at 10 μM for 6 h, followed by stimulation with cGAMP (1 h). Whole-cell lysates were analyzed by immunoblotting. (**B**) HEK293T cells were transfected with Flag-tagged wild-type EGFR or mEGFR, along with Myc-tagged TBK1. Cell lysates were analyzed by conventional SDS-PAGE or Phos-tag gel electrophoresis to assess TBK1 phosphorylation. (**C**) HEK293T cells were co-transfected with Flag-tagged wild-type or mutant EGFR, along with TBK1-Myc or IKKε-Myc. Lysates were immunoprecipitated with anti-Myc and analyzed by immunoblotting with anti-phosphotyrosine (pY100) and anti-EGFR to detect tyrosine phosphorylation of TBK1 and IKKε. (**D**) HEK293T cells were co-transfected with HA-tagged STING with either wild-type or mutant EGFR. HA immunoprecipitants were probed with pY100 and STING antibodies to assess STING tyrosine phosphorylation. (**E**) H1975 and H1299 cells were treated with or without WZ4002 (10 μM, 6 h). Lysates were immunoprecipitated with pY100 antibody and analyzed for STING, TBK1, and EGFR by immunoblotting. (**F**) In vitro kinase assay using immunopurified Flag-tagged mEGFR, STING, or TBK1 isolated from HEK293T cells, as indicated. Reaction products were analyzed by immunoblotting with anti-pY100 to detect tyrosine phosphorylation. Longer exposure (l.e.) is shown for the pY100 signal. (**G**) Mass spectrometry analysis of phosphorylation sites in TBK1 and STING with or without mEGFR co-expression. For TBK1 (upper heatmap), HEK293T cells were co-transfected with TBK1-Flag in the absence or presence of mEGFR-Flag, and TBK1 immunoprecipitates were subjected to mass spectrometry. For STING (lower heatmap), HEK293T cells were transfected with STING-HA alone or with mEGFR-Flag, and the cells were then analyzed by mass spectrometry. Heatmaps indicate the relative phosphorylation levels (%) at the indicated residues. (**H**) HEK293T cells were co-transfected with mEGFR together with TBK1-Myc (WT) or the indicated tyrosine mutants (Y406F, Y577F, Y677F, or Y577F/Y677F). TBK1 was immunoprecipitated using an anti-Myc antibody, and tyrosine phosphorylation was detected by immunoblotting with anti-pY100. (**I**) Wild-type or mutant TBK1 constructs (Y577F, Y677F, or Y577F/Y677F) were co-expressed with mEGFR in HEK293T cells. TBK1 phosphorylation was reduced in single mutants and diminished in the double mutant. YF tyrosine to phenylalanine. (**J**) HEK293T cells were co-transfected with mEGFR together with STING-HA (WT) or the indicated tyrosine mutants (Y245F, Y186F/Y245F, or Y245F/Y314F). STING was immunoprecipitated using an anti-HA antibody, and tyrosine phosphorylation was detected by anti-pY100 immunoblotting. (**K**) HA-tagged wild-type STING or phosphomimetic mutants (Y245E, Y314E) were co-expressed with Flag-tagged EGFR L858R/T790M. HA immunoprecipitates were blotted for EGFR and STING to assess protein interaction. STING mutants. YE: tyrosine to glutamate. (**L**) HA-tagged wild-type or phosphomimetic STING mutants were co-expressed with Myc-tagged TBK1. HA immunoprecipitation followed by immunoblotting revealed enhanced binding of TBK1 to STING Y245E/Y314E compared to wild-type STING. Data information: unless otherwise indicated, *n*  =  3 independent biological experiments. Densitometric values are indicated below the bands and provided in the source data. Source data are available online for this figure.

To elucidate mechanisms by which mEGFR associates with the STING machinery and modulates STING signaling, we first used an array of inhibitors targeting mEGFR itself or its downstream signaling components, including AKT, ERK1/2, and STAT3/5 (Tomas et al, 2014). Inhibitors targeting mEGFR kinase activity impeded STING-induced phosphorylation of TBK1 and IRF3, while inhibitors acting on AKT, Erk1/2, and STAT3/5 did not (Fig. 3A). Phos-tag PAGE analyses further confirmed a substantial increase in TBK1 phosphorylation levels (phospho-S172) upon mEGFR co-expression, rather than wild-type EGFR (Fig. 3B). Noticeably, we detected a robust phospho-tyrosine signal (pY100) on both TBK1 (Fig. 3C) and STING proteins (Fig. 3D) following mEGFR expression, but not in IKKε or by wild-type EGFR. This tyrosine phosphorylation in endogenous STING and TBK1 proteins was seen in H1975 NSCLC cells harboring mEGFR but not in H1299 NSCLC cells harboring wtEGFR, while WZ4002 diminished them (Fig. 3E), suggesting a mEGFR-driven nature. We then performed an in vitro kinase assay using immunopurified Flag-tagged mEGFR, STING, and TBK1. Under these conditions, a pY100 signal of both STING and TBK1 was detected only in the presence of mEGFR (Fig. 3F).

Next, mass spectrometry analyses identified several tyrosine residues in TBK1 and STING (Fig. 3G) as potential mEGFR phosphorylation targets. To functionally interrogate these sites, we generated tyrosine (Y) to phenylalanine (F) mutants guided by mass spectrometry data, as well as tyrosine (Y) to glutamate (E) phosphomimetic mutants. In the presence of co-expressed mEGFR, TBK1 Y577F, Y677F, and especially the double mutant Y577F/Y677F, showed reduced pY100 reactivity compared with WT TBK1, indicating that these residues contribute to mEGFR-dependent tyrosine phosphorylation (Fig. 3H). Intriguingly, the Y577 residue of TBK1 forms a cation–π interaction with R375, a residue important for TBK1 incorporation into the STING signalosomes (Zhang et al, 2019). By contrast, mEGFR-enhanced TBK1 activation was reduced in the Y577F/Y677F mutant (Fig. 3I), suggesting Y577 and Y677 as candidate residues involved in mEGFR-mediated TBK1 modification. Meanwhile, immunoblotting following coimmunoprecipitations indicated that the STING Y245 and Y314 residues were the major candidates contributing to mEGFR-mediated phosphorylation (Fig. 3J). The phosphomimetic mutants of STING Y245E and Y314E exhibited increased association with both mEGFR (Fig. 3K) and TBK1 (Fig. 3L) relative to wild-type STING. These reconstitution-based data suggest that mEGFR-dependent tyrosine phosphorylation of STING and TBK1 may reinforce pre-existing association within the STING–TBK1 signaling complex and thereby facilitate signalosome assembly.

### Phosphorylation at Y577/Y677 residues stabilizes activated TBK1

**Figure 4:**
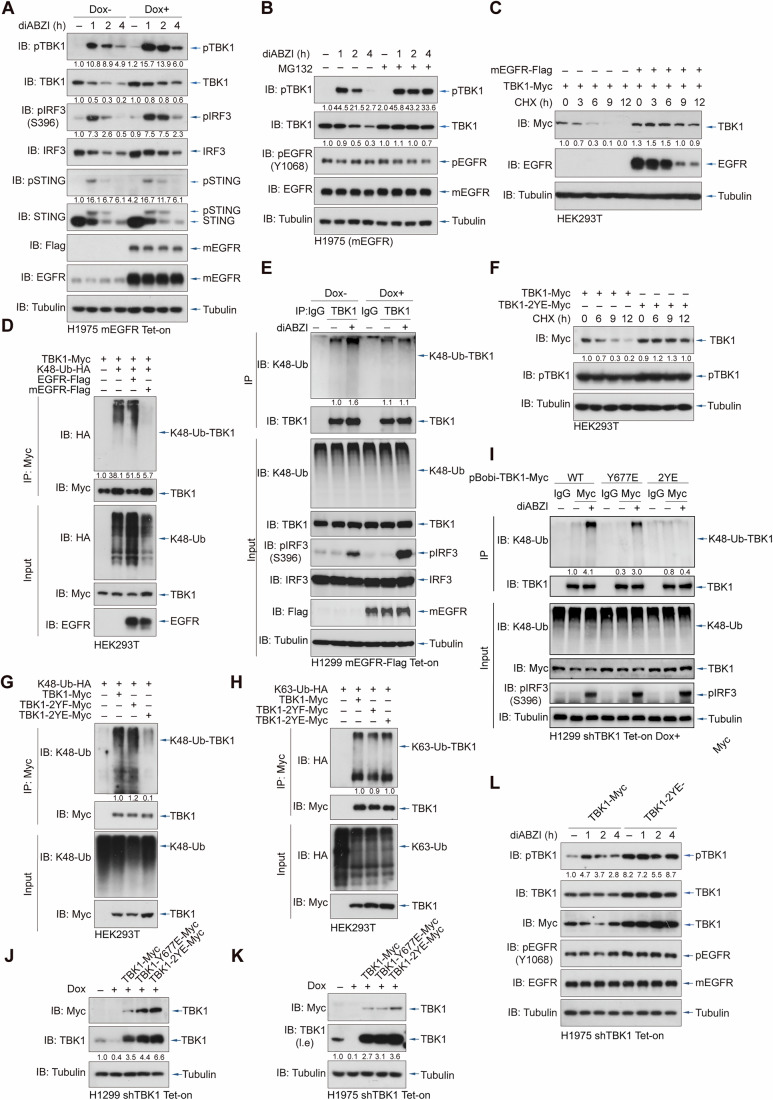
mEGFR-mediated phosphorylation hyperactivates and stabilizes TBK1. (**A**) H1975 cells stably expressing doxycycline-inducible mEGFR were treated with diABZI for the indicated time points with or without doxycycline pre-treatment (48 h). TBK1 protein levels were analyzed by immunoblotting to assess degradation dynamics. (**B**) H1975 cells were treated with diABZI for the indicated time points in the presence or absence of MG132 (10 μM, 6 h) to assess the effect of proteasomal inhibition on TBK1 degradation. (**C**) HEK293T cells were co-transfected with TBK1-Myc with or without Flag-tagged mEGFR. Cells were treated with cycloheximide (CHX) for the indicated time points to assess TBK1 protein stability by immunoblotting. (**D**) HEK293T cells were co-transfected with TBK1-Myc, HA-tagged K48-Ubiquitin (K48-Ub), and either wild-type or mutant EGFR. TBK1 ubiquitination was examined by immunoprecipitation with anti-Myc followed by anti-K48-Ub immunoblotting. (**E**) H1299 cells stably expressing doxycycline-inducible mEGFR were treated with diABZI (1 h) with or without doxycycline induction. Endogenous TBK1 was immunoprecipitated and analyzed for K48-linked ubiquitination. (**F**) HEK293T cells were transfected with either wild-type TBK1 or TBK1 phosphomimetic mutant (Y577E/Y677E, designated as TBK1-2YE). CHX chase assays and immunoblotting assessed TBK1 protein stability. (**G**) TBK1-Myc (wild-type, 2YF, or 2YE) was co-expressed with K48-Ub in HEK293T cells. TBK1 was immunoprecipitated and analyzed for K48-linked ubiquitination by immunoblotting to evaluate the role of phosphorylation in ubiquitin modification. (**H**) K63-linked ubiquitination of TBK1 was examined by co-transfecting K63-Ub-HA with TBK1 variants (WT, 2YF, or 2YE) in HEK293T cells. Immunoprecipitated TBK1 was analyzed for K63-Ub by immunoblotting. (**I**) H1299 cells with doxycycline-inducible knockdown of endogenous TBK1 were reconstituted with shRNA-resistant TBK1-Myc constructs (WT, Y677E, or 2YE). Cells were treated with diABZI (1 h), followed by immunoprecipitation using anti-Myc and analysis of K48-linked ubiquitination. (**J**, **K**) The steady-state expression levels of TBK1 variants (WT, Y677E, 2YE) were analyzed by immunoblotting in H1299 (**J**) or H1975 (**K**) Tet-On shTBK1 cells after 72 h of doxycycline-induced knockdown of endogenous TBK1. (**L**) H1975 shTBK1 Tet-On cells were reconstituted with TBK1-Myc (WT) or TBK1-2YE-Myc and treated with diABZI for the indicated times. Data information: unless otherwise indicated, *n*  =  3 independent biological experiments. Densitometric values are indicated below the bands and provided in the source data. Source data are available online for this figure.

Besides enhancing STING signalosome assembly that facilitates TBK1 activation, does mEGFR also regulate the magnitude or duration of TBK1 activation? TBK1 is degraded by the ubiquitin-proteasomal system (Deng et al, 2020; Cui et al, 2012). We observed that inducible mEGFR expression diminished diABZI-induced degradation of TBK1 (Fig. 4A; Appendix Fig. S1A). Similarly, blocking proteasome function with MG132 inhibited diABZI- and cGAMP-triggered degradation of endogenous TBK1 in H1975 cells (Fig. 4B; Appendix Fig. S1B), suggesting a role for mEGFR in stabilizing activated TBK1 upon STING activation. In the presence of cycloheximide, which blocked de novo protein synthesis, mEGFR significantly delayed TBK1 degradation (Fig. 4C). Furthermore, mEGFR, rather than wild-type EGFR, notably inhibited the K48-Ub modification of transfected TBK1 proteins in HEK293T cells (Fig. 4D), as well as endogenous TBK1 proteins in NSCLC cells (Fig. 4E). Noticeably, phosphomimetic TBK1 mutant (Y577E/Y677E, 2YE) exhibited a markedly extended half-life under cycloheximide chase assay (Fig. 4F). Consistently, a minimal level of K48-linked ubiquitination on the phosphomimetic TBK1 2YE mutant was detected when compared to WT or 2YF TBK1 mutant (Fig. 4G). However, their K63-linked ubiquitination remained comparable (Fig. 4H). Lentivirus-delivered reconstitution of mutant TBK1 in TBK1-deleted H1299 cells also indicated that STING agonist-induced K48-Ub modification on TBK1 was diminished in the case of TBK1-2YE (Fig. 4I), with reconstituted TBK1-2YE showing higher protein levels than wild-type TBK1 in both H1299 and H1975 (Fig. 4J,K). These findings collectively suggest that mEGFR-mediated phosphorylation of TBK1 at Y577/Y677 protects TBK1 from K48-Ub modification and proteasomal degradation, thereby sustaining hyperactive STING–TBK1 signaling.

### STING and TBK1 are both required for mEGFR-driven NSCLC survival

**Figure 5:**
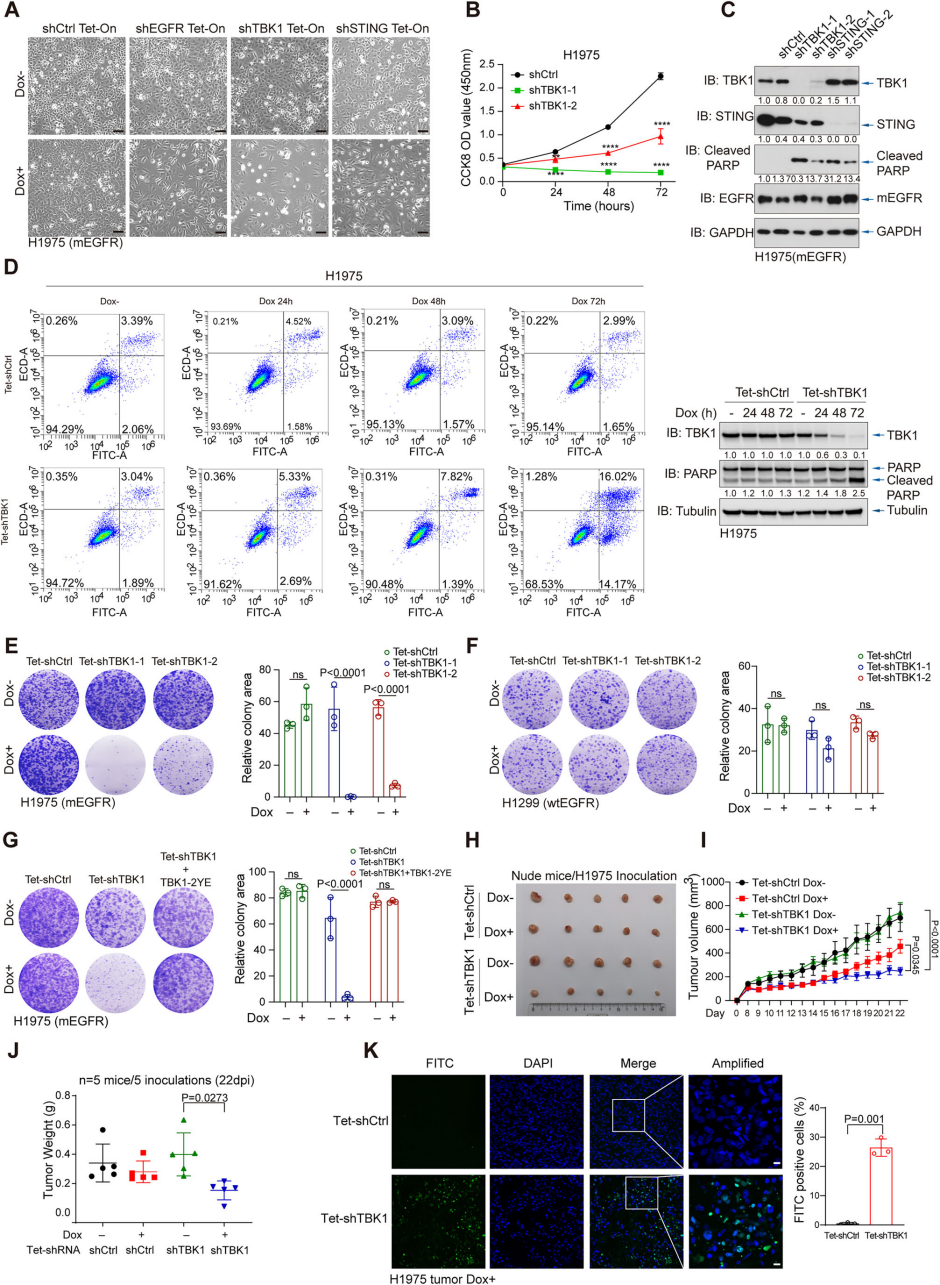
STING and TBK1 are indispensable for mEGFR-driven NSCLC survival. (**A**) Brightfield images of H1975 cells expressing inducible shRNAs targeting EGFR, TBK1, STING, or a non-targeting control (shCtrl). Cells were treated with Dox or left untreated for 72 h. Scale bar, 100 μm. (**B**) Cell viability of H1975 cells expressing two independent shRNAs targeting TBK1 (shTBK1) or shCtrl was assessed at the indicated time points using a CCK-8 assay. Data represent mean ± SEM. Statistical significance was determined by two-way ANOVA with Šídák’s multiple comparisons test. **P < 0.001, and ****P < 0.0001. Exact *P* values are provided in the source data. (**C**) Immunoblotting confirmed the knockdown efficiency of TBK1 and STING in H1975 cells. Apoptosis was evaluated by detection of cleaved PARP following TBK1 or STING depletion. (**D**) Apoptosis in H1975 cells expressing Tet-On shCtrl or shTBK1 was analyzed at 24, 48, and 72 h of Dox induction using Annexin V-FITC/PI staining and flow cytometry. Percentages of Annexin V-FITC/PI positive cells are indicated. Right: Corresponding TBK1 levels were validated by immunoblotting. (**E**, **F**) Colony formation assays of H1975 (**E**) and H1299 (**F**) cells expressing inducible shCtrl or two independent shTBK1 constructs, with or without Dox treatment. The bar graph shows quantification of colony formation (right panels). (**G**) Colony formation assays of H1975 cells with TBK1 knockdown and reconstitution of an shRNA-resistant TBK1-2YE. The bar graph shows quantification of colony formation (right panels). (**H**) Representative images of excised bilateral xenograft tumors at endpoint. *n* = 5 tumors per group, derived from 10 mice with H1975 cells expressing Tet-On shCtrl or shTBK1 xenografts implanted on opposite flanks. (**I**) Mice were fed with or without doxycycline-containing (1 mg/mL) water for 22 days post-inoculation. Tumor growth curves of H1975 xenografts with or without Dox-induced TBK1 depletion (*n* = 5 tumors per group). Tumor volumes were measured at the indicated time points. (**J**) Tumor weights of H1975 xenografts collected at 22 days post-inoculation (dpi) from mice treated with or without doxycycline (*n* = 5 tumors per group). (**K**) FITC-labeled TUNEL staining of tumor sections (*n* = 3 tumors per group) from H1975 xenografts expressing Tet-On shCtrl or shTBK1. DAPI (blue) stains nuclei, and FITC (green) indicates apoptotic cells. Scale bars, 10 μm. Quantification of FITC-positive cells is shown on the right panel. Data in (**E**–**G**, **I**, **J**) are presented as mean ± SEM and were analyzed by two-way ANOVA with Tukey’s multiple comparisons test. Exact *P* values are indicated in the respective panels. Data in (**K**) are presented as mean ± SEM and were analyzed by an unpaired two-tailed *t* test. The exact *P* value is indicated in (**K**). Unless otherwise indicated, *n* = 3 independent biological experiments. Densitometric values are indicated below the bands and provided in the source data. Source data are available online for this figure.

**Figure EV3:**
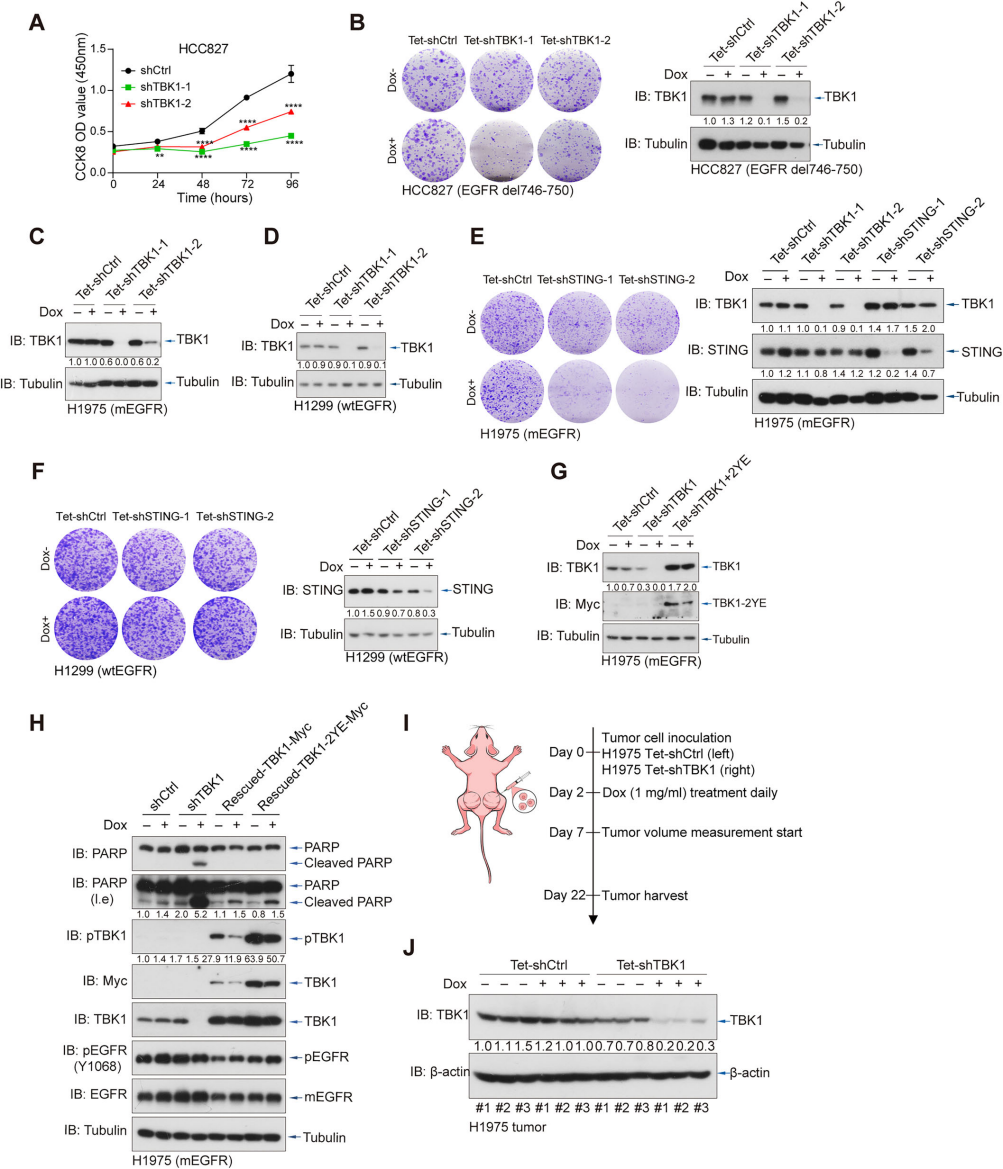
STING and TBK1 are indispensable for mEGFR-driven NSCLC survival. (**A**) CCK-8 assays measured cell viability in mutant EGFR-driven HCC827 NSCLC cells with shCtrl or shTBK1. Data are presented as mean ± SEM. Statistical significance was determined by two-way ANOVA with Šídák’s multiple comparisons test. **P < 0.01, and ****P < 0.0001. Exact P values are provided in the source data. (**B**) Colony formation assays of HCC827 cells expressing inducible shCtrl or two independent shTBK1 constructs, with or without Dox treatment. (**C**, **D**) Knockdown efficiency of TBK1 in H1975 (**C**) and H1299 (**D**) was confirmed by immunoblotting. (**E**, **F**) Colony formation assays were performed as in Fig. 5E in doxycycline-inducible shRNA cell lines targeting STING in H1975 (**E**) and H1299 (**F**) cells. Two independent shRNAs were used, and knockdown efficiency was confirmed by immunoblotting (right). (**G**) Knockdown and rescue efficiency were confirmed by immunoblotting in H1975 cells. (**H**) Doxycycline-inducible TBK1 knockdown H1975 (mEGFR) cells were reconstituted with an shRNA-resistant TBK1-Myc (WT) or TBK1-2YE-Myc rescue construct, as indicated. Whole-cell lysates were analyzed by immunoblotting to assess PARP cleavage and TBK1 expression. (**I**) Schematic illustration of the in vivo experimental timeline for H1975 xenograft implantation and doxycycline treatment. (**J**) Immunoblotting analysis of TBK1 protein levels in individual H1975 tumor samples (*n* = 3 per group) with or without doxycycline treatment to induce shTBK1 expression. Representative colony formation images and corresponding immunoblots from two independent experiments with similar results are shown. Data information: unless otherwise indicated, *n*  =  3 independent biological experiments. Densitometric values are indicated below the bands and provided in the source data. Source data are available online for this figure.

Hyperactivation of EGFR is a significant driver of cancer development, promoting tumorigenesis through uncontrolled proliferation, survival, and metastasis (Roskoski, 2014). However, our findings above suggest an unexpected role for mEGFR in promoting cGAS–STING signaling, an innate immune system poised to support antitumor immunity. Does mEGFR-potentiated cGAS–STING signaling contribute to protumorigenic functions? Noticeably, we found that doxycycline-induced shRNA depletion of either TBK1 or STING substantially inhibited H1975 growth to a level comparable to mEGFR depletion (Fig. 5A), while TBK1 depletion significantly reduced cell viability in mEGFR-driven NSCLC cells harboring L858R/T790M (Fig. 5B) and delE746-A750 (Fig. EV3A). Moreover, an elevated level of cleaved-PARP in H1975 cells following TBK1 or STING depletion was detected (Fig. 5C), and Annexin-FITC/PI assays revealed apparent apoptosis of mEGFR-driven H1975 NSCLC cells following doxycycline-induced TBK1 depletion (Fig. 5D), suggesting mEGFR-activated STING signalosomes in preventing NSCLC apoptotic death. In addition, TBK1 depletion potently impeded colony formation of mEGFR-driven H1975 (Figs. 5E and EV3C) and HCC827 (Fig. EV3B) NSCLC cells, while barely impacting colony formation in H1299 cells harboring wild-type EGFR, suggesting a unique dependency and vulnerability of mEGFR-driven cells on TBK1 (Figs. 5F and EV3D). Inducible depletion of STING phenocopied this growth defect in H1975 cells (Fig. EV3E). In contrast, STING depletion did not impair colony formation to a similar extent in wtEGFR H1299 cells (Fig. EV3F). Given that mEGFR stabilizes TBK1 via phosphorylation at Y577/Y677, we next performed rescue experiments. The reconstitution of the TBK1 2YE mutant into H1975 cells, combined with a silent mutation of the shRNA recognition site, wholly rescued the colony formation defect caused by TBK1 depletion (Figs. 5G and EV3G), suggesting an essential role of mEGFR-directed TBK1 stability in survival regulation. Likewise, reconstitution of either TBK1 or TBK1-2YE in the shTBK1 background attenuated TBK1 depletion-associated apoptosis readouts (Fig. EV3H).

Next, we employed an in vivo immune-independent model to validate the proposed role of STING signalosomes in mEGFR-driven NSCLC viability. H1975 cells with inducible shCtrl or shTBK1 shRNA were subcutaneously injected into immune-incompetent mice, and doxycycline-induced TBK1 depletion in H1975 cells significantly attenuated tumor growth and tumor weight (Figs. 5H–J and EV3I,J). Apoptosis levels in mEGFR-driven H1975 tumors upon TBK1 depletion were also verified by TUNEL staining, an effect not observed in shCtrl tumors (Fig. 5K). Collectively, these findings establish STING signalosomes as an indispensable factor, through TBK1 activation, for the maintenance and progression of mEGFR-driven NSCLC cells.

### TBK1 supports DDR-associated responses and cisplatin resistance in mEGFR-driven NSCLC

**Figure 6:**
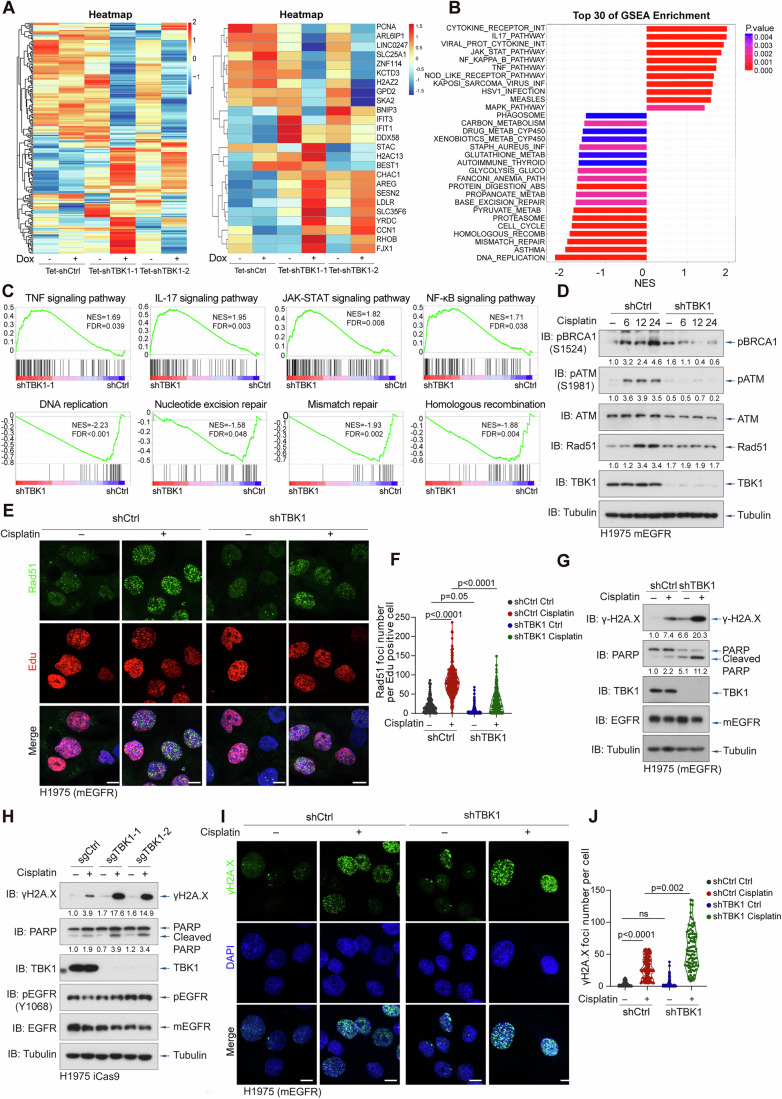
TBK1 supports DDR-associated responses and survival in mEGFR-driven NSCLC. (**A**) Heatmap of transcriptomic profiles from H1975 cells expressing doxycycline-inducible TBK1 shRNAs (Tet-shTBK1-1 and Tet-shTBK1-2) or control shRNA (Tet-shCtrl), with or without Dox induction (*n* = 2). Left: hierarchical clustering of differentially expressed genes. Right: top upregulated and downregulated genes. Color scale denotes relative expression levels from low (blue) to high (red). (**B**) Gene Set Enrichment Analysis (GSEA) revealed significant suppression of DNA repair pathways upon TBK1 depletion in H1975 cells. GSEA was performed using a weighted Kolmogorov–Smirnov statistic with 1000 gene-set permutations. Significance was assessed by permutation-derived nominal *P* values and Benjamini–Hochberg-adjusted FDR. (**C**) Representative GSEA plots demonstrating enrichment of inflammatory signaling and impairment of DNA damage repair pathways in TBK1-depleted cells. NES normalized enrichment score, FDR false discovery rate. (**D**) H1975 cells expressing control shCtrl or shTBK1 were treated with cisplatin for the indicated time points and analyzed by immunoblotting as indicated to monitor activation of the DNA damage response and homologous recombination markers. (**E**) H1975 cells expressing shCtrl or shTBK1 were left untreated or treated with cisplatin (24 h) and stained for Rad51 (green). EdU (red) marks S-phase cells; nuclei were counterstained with DAPI (blue). Representative images are shown. Scale bars, 10 μm. (**F**) Quantification of Rad51 foci in EdU+ cells under the indicated conditions. Each dot represents one cell. Data were from three independent experiments (*n* = 169, 266, 145, and 247 cells, respectively). Statistical significance was determined by the Kruskal–Wallis test with Dunn’s multiple comparisons test. Exact *P* values are indicated in the panel. (**G**) H1975 cells expressing shCtrl or shTBK1 were left untreated or treated with cisplatin (48 h), followed by immunoblotting analysis as indicated to assess γH2A.X and PARP cleavage. (**H**) H1975 iCas9 cells expressing sgCtrl or two independent sgTBK1 constructs (sgTBK1-1 and sgTBK1-2) were left untreated or treated with cisplatin (48 h), followed by immunoblotting analysis as indicated to assess γH2A.X and PARP cleavage. (**I**) H1975 cells expressing shCtrl or shTBK1 were left untreated or treated with cisplatin (48 h) and stained for γH2A.X (green); nuclei were counterstained with DAPI (blue). Representative images are shown. Scale bars, 10 μm. (**J**) Quantification of γH2A.X foci per nucleus under the indicated conditions. Each dot represents one cell. Data were pooled from three independent experiments (*n* = 117, 110, 94, and 83 cells, respectively). Statistical significance was determined by the Kruskal–Wallis test with Dunn’s multiple comparisons test. Exact *P* values are indicated in the panel. Unless otherwise indicated, *n*  =  3 independent biological experiments. Densitometric values are indicated below the bands and provided in the source data. Source data are available online for this figure.

**Figure EV4:**
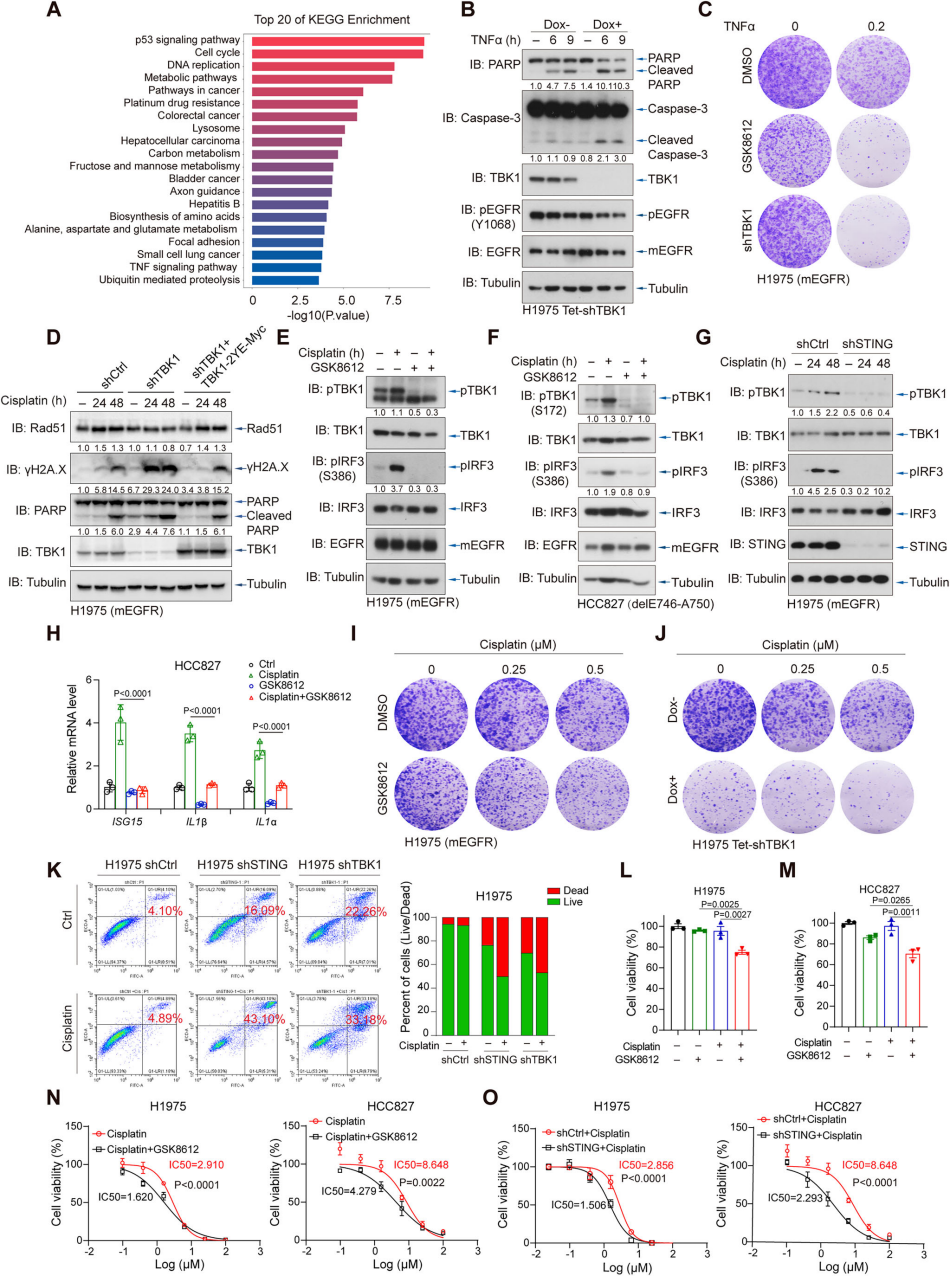
TBK1 supports DDR-associated responses and survival in mEGFR-driven NSCLC. (**A**) Transcriptomic analyses of KEGG-enriched pathways in H1975 cells with TBK1 depletion indicated suppression of oncogenic and survival pathways. *Y* axis: enriched pathways; *X* axis: enrichment scores; color intensity denotes *P* value significance. Enrichment significance was assessed using Fisher’s exact test. (**B**) Immunoblotting of H1975 Tet-shTBK1 cells treated with TNFα in the presence or absence of Dox for the indicated time points. TBK1 depletion exacerbated TNFα-induced apoptosis, as evidenced by increased levels of cleaved PARP and cleaved caspase-3. (**C**) Colony formation assays in H1975 cells treated with TNFα and DMSO, GSK8612, or TBK1 shRNA. Both pharmacological inhibition and genetic depletion of TBK1 sensitized cells to TNFα-induced growth suppression. (**D**) H1975 cells expressing shCtrl or shTBK1, or shTBK1 together with shRNA-resistant TBK1-2YE–Myc were treated with cisplatin (8 μM) for 24 h or 48 h, followed by immunoblotting analysis as indicated. (**E**, **F**) Immunoblotting of H1975 (**E**) and HCC827 (**F**) cells treated with cisplatin (8 μM, 48 h), with or without GSK8612 (5 μM), to assess TBK1-IRF3 pathway activation. (**G**) Immunoblotting analysis of H1975 cells expressing shCtrl or shSTING and treated with cisplatin (8 μM) for the indicated time points, showing STING-dependent activation of TBK1 signaling upon cisplatin treatment. (**H**) Quantitative RT-PCR analysis of interferon-stimulated genes (*ISG15*, *IL1β*, and *IL1α*) in HCC827 cells treated with cisplatin (8 μM), GSK8612 (5 μM), or the combination. TBK1 inhibition suppressed cisplatin-induced ISG expression. (**I**) Colony formation assays in H1975 (mEGFR) cells treated with cisplatin at the indicated doses in the presence of DMSO or GSK8612 (5 µM). (**J**) Colony formation assays in H1975 Tet-shTBK1 cells treated with cisplatin at the indicated doses, with Dox-/Dox+ to control TBK1 knockdown. (**K**) Flow cytometry analysis of Annexin V/PI-stained H1975 cells expressing shCtrl, shSTING, or shTBK1, treated with cisplatin (1 µM, 48 h). Right: Quantification of live (Annexin V−/PI−) and dead (Annexin V+ and/or PI + ) cell fractions. (**L**, **M**) CCK-8 assays measuring viability of H1975 (**L**) and HCC827 (**M**) cells treated with cisplatin (0.4 µM) and/or GSK8612 (5 µM). For bar graphs in (**L**, **M**), values were extracted from the indicated dose points in the corresponding dose–response experiments shown in (**N**). (**N**) Dose–response curves of cisplatin alone or combined with GSK8612 in H1975 and HCC827 cells. Co-treatment significantly decreased cisplatin IC50, suggesting enhanced sensitivity. (**O**) Dose–response curves of cisplatin in H1975 and HCC827 cells expressing control or STING-targeting shRNAs. Data in (**N**, **O**) are presented as mean ± SEM. Differences in logIC50 values were assessed by an extra sum-of-squares *F* test. Exact *P* values for (**N**, **O**) are indicated in the panels. Data in (**H**, **L**, **M**) are presented as mean ± SEM. Statistical significance in (**H**, **L**, **M**) was determined by one-way ANOVA with Tukey’s multiple comparisons test. Exact *P* values in (**H**, **L**, **M**) are indicated in the respective panels. Representative colony formation images in (**C**, **I**, **J**) are shown from two independent experiments with similar results. Unless otherwise indicated, *n*  =  3 independent biological experiments. Densitometric values are indicated below the bands and provided in the source data. Source data are available online for this figure.

Next, we performed transcriptional profiling in H1975 cells to assess the impact of TBK1 depletion on the transcriptional landscape. RNA-seq analyses showed substantial alterations in gene expression upon TBK1 depletion (Fig. 6A). KEGG enrichment analysis indicated significant enrichment for pathways related to p53 signaling, cell cycle regulation, and DNA replication (Fig. EV4A). Gene Set Enrichment Analysis (GSEA) further highlighted activation of TNF and IL-17 signaling, as well as other pathways associated with inflammation and immune responses. Intriguingly, a significant downregulation of DNA repair pathways was observed following TBK1 depletion, including base excision repair, homologous recombination repair, and mismatch repair (Fig. 6B,C). Consistent with GSEA findings, we observed a marked increase in apoptotic markers in TNFα-treated TBK1-deficient cells (Fig. EV4B). Colony formation assays also showed a substantial reduction in colony number when the TBK1 inhibitor GSK8612 or TBK1 depletion was combined with TNFα treatment (Fig. EV4C).

Cisplatin is a well-established clinical chemotherapeutic agent in NSCLC, particularly in cases without targetable mutations, ineligible for immunotherapy, or with targeted therapy resistance (Lu et al, 2022). Efficient DNA repair is a key determinant of cisplatin resistance (Kelland, 2007). We next examined how loss of TBK1 affects DNA damage response (DDR) after cisplatin treatment. In control H1975 cells, cisplatintriggered canonical DNA damage signaling and repair-associated responses, including induction of pATM and pBRCA1 and accumulation of Rad51 over time. We found that these responses were significantly blunted upon TBK1 depletion (Fig. 6D), consistent with impaired DNA damage response and repair-associated signaling. Cisplatin robustly increased Rad51 foci in EdU+ cells, whereas TBK1 knockdown markedly reduced the formation of these Rad51 foci (Fig. 6E,F), supporting a compromised homologous recombination-associated repair during S phase. In parallel, TBK1 loss exacerbated DNA damage and apoptosis markers after cisplatin treatment, including increased γH2A.X, PARP cleavage (Fig. 6G,H), and increased γH2A.X focus burden per nucleus (Fig. 6I,J). To strengthen the link between TBK1 and these DDR-associated phenotypes, we performed a rescue experiment using shRNA-resistant TBK1-2YE in shTBK1 H1975 cells. Upon cisplatin treatment, re-expression of TBK1-2YE partially restored Rad51 and partially attenuated the increases in γH2A.X and PARP cleavage observed upon TBK1 depletion at both 24 h and 48 h (Fig. EV4D). These results further support a functional role for TBK1 in DDR-associated responses in this setting.

Given that cisplatin tolerance requires effective DNA repair, we asked whether cisplatin engages the STING–TBK1 axis in this setting. Noticeably, we found that cisplatin robustly activated cGAS–STING–TBK1–IRF3 signaling in mEGFR-driven H1975 and HCC827 NSCLC cells (Fig. EV4E,F), as well as TBK1-dependent expression of interferon-stimulated genes (Fig. EV4H). Moreover, STING depletion reduced cisplatin-induced TBK1/IRF3 activation (Fig. EV4G), supporting that cisplatin-triggered signaling engages STING signaling.

Finally, we examined whether targeting TBK1 and STING sensitizes cells to cisplatin. Combinational treatment of cisplatin and TBK1 inhibition substantially and synergistically attenuated colony formation in H1975 cells (Fig. EV4I). Likewise, TBK1 deficiency induced by doxycycline-mediated shRNA rendered H1975 cells more sensitive to cisplatin-induced cytotoxicity (Fig. EV4J). Consistent with the clonogenic sensitization, Annexin V/PI analyses showed that depletion of STING or TBK1 increased cell death in H1975 cells upon low-dose cisplatin treatment (Fig. EV4K). In both H1975 and HCC827 cells, treatment with cisplatin or GSK8612 alone led to modest reductions in cell viability, whereas their combination produced a greater reduction in viability (Fig. EV4L,M). Dose–response curves were consistent with increased cisplatin sensitivity upon TBK1 inhibition (Fig. EV4N) or STING depletion (Fig. EV4O), although the magnitude of the effect varied across conditions. These data suggest that targeting STING–TBK1 signaling in mEGFR-driven NSCLC impairs DNA damage repair and sensitizes tumors to chemotherapeutic agents.

### Combining cisplatin and TBK1i diminishes TKI-resistant NSCLC PDOs and mEGFR-driven spontaneous NSCLC tumors

**Figure EV5:**
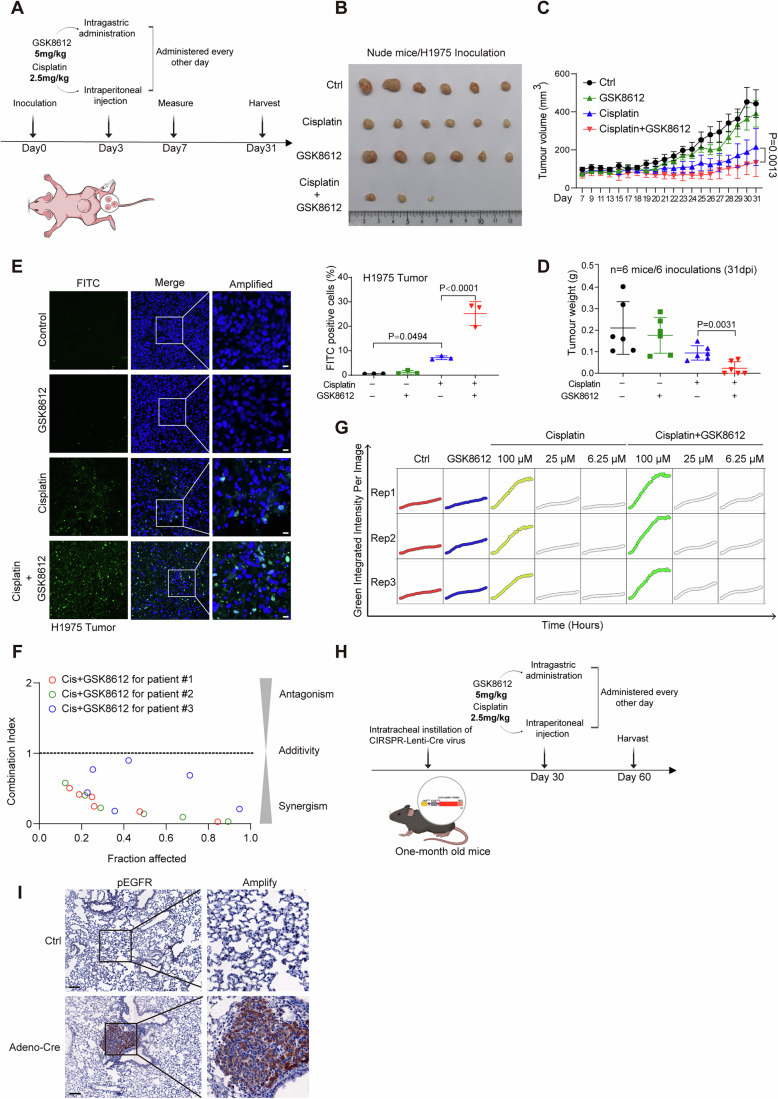
Combining cisplatin and TBK1i diminishes mEGFR-driven NSCLC PDOs and spontaneous tumors. (**A**) Schematic of the experimental design for the H1975 xenograft model. Nude mice were subcutaneously inoculated with H1975 NSCLC cells and treated every other day with GSK8612 (5 mg/kg, intragastric) and/or cisplatin (2.5 mg/kg, intraperitoneal), starting on day 3 post-inoculation. (**B**) Representative images of excised tumors at day 31 post-inoculation (dpi) (*n* = 6 mice per group). Mice receiving combination therapy (cisplatin + GSK8612) exhibited the most significant tumor suppression. (**C**, **D**) Quantification of tumor progression in xenografts. Tumor volume was measured at the indicated time points (**C**), and tumor weight was assessed at 31 dpi (**D**). Combination treatment significantly reduced both parameters compared to monotherapies (*n* = 6 mice per group). (**E**) TUNEL staining of xenograft tumor tissues showed markedly increased apoptosis in the combination group, as indicated by elevated FITC-positive cells (green). Scale bars, 10 µm. Quantification shown on the right. (**F**) Drug interaction analysis using CompuSyn software demonstrated synergism (combination index, CI < 1) between cisplatin and GSK8612 across a range of concentrations in EGFR-mutant NSCLC PDOs from three patients. (**G**) Quantification of caspase-3/7 activation over time in PDOs (Patient #1) treated with cisplatin (at indicated concentrations) and GSK8612 (5 μM). Data are shown from three biological replicates using a live-cell apoptosis detection assay. (**H**) Schematic of the treatment schedule in the spontaneous mEGFR NSCLC mouse model. One-month-old mice were intratracheally instilled with CRISPR-Lenti-Cre virus to induce lung-specific expression of EGFR L858R/T790M. On day 30, mice received GSK8612 and/or cisplatin every other day until sacrifice on day 60. (**I**) Immunohistochemistry (IHC) of lung tissues from control or Adeno-Cre-infected mice revealed strong pEGFR expression in tumor regions of the Cre-treated group. Scale bars, 100 µm. Data in (**C**–**E**) are presented as mean ± SEM. Statistical significance in (**C**) was determined by two-way ANOVA, and (**D**, **E**) were determined by one-way ANOVA with Tukey’s multiple comparisons test. Exact *P* values in (**C**–**E**) are indicated in the corresponding panel. Unless otherwise indicated, *n*  =  3 independent biological experiments. Source data are available online for this figure.

H1975 tumors engrafted in nude mice indicated that the TBK1 inhibitor GSK8612 substantially enhanced the effect of cisplatin, achieving complete elimination in half of the tumors after a month-long regimen (Fig. EV5A,B). Quantitative assessments of tumor volume and weight revealed a superior efficacy of the drug combination compared with monotherapy (Fig. EV5C,D). TUNEL staining showed a marked increase in the apoptosis ratio in tumor tissue (Fig. EV5E), suggesting an enhanced antitumor effect of the cisplatin and GSK8612 combination therapy in an artificial immunodeficient environment.

**Figure 7:**
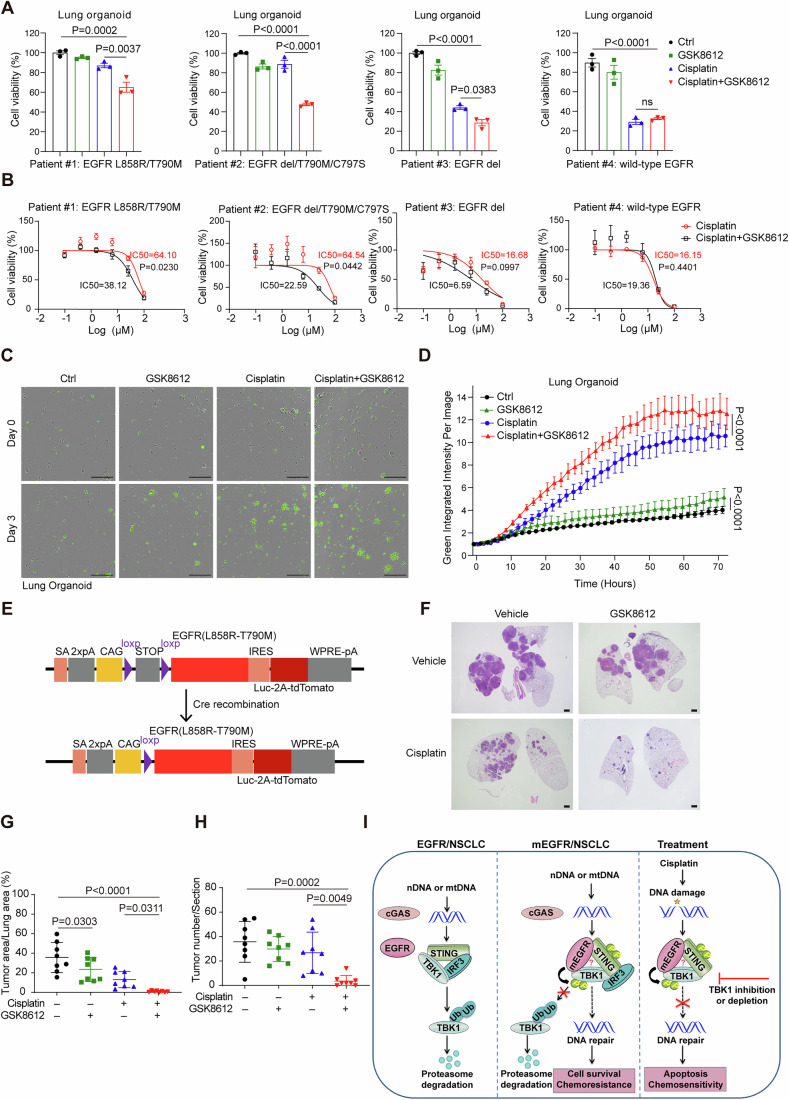
Combining cisplatin and TBK1i diminishes mEGFR-driven NSCLC PDO viability and spontaneous tumors. (**A**) Cell viability assays using CellTiter-Lumi™ were performed on patient-derived lung organoids (PDOs) from NSCLC patients bearing EGFR mutations (L858R/T790M, exon 19 del/T790M/C797S, or exon 19 del) or wild-type EGFR. Combination treatment with cisplatin (25 μM) and TBK1 inhibitor GSK8612 (5 μM) significantly reduced cell viability in PDOs with mutant EGFR, but not in wild-type EGFR organoids. ns not significant. (**A**) Shows the selected 25 μM cisplatin dose point from the patient-derived organoid dose–response experiments shown in (**B**). (**B**) Dose–response curves for cisplatin alone or combined with GSK8612 were generated for each PDO, and IC50 values are indicated. Dose–response curves were fitted by nonlinear regression. Data are presented as mean ± SEM. An extra sum-of-squares *F* test assessed differences in logIC50 between cisplatin and cisplatin + GSK8612. Exact *P* values are indicated in the respective panel. (**C**) Representative fluorescence images of PDOs (Patient #1) treated with indicated conditions for 72 h, stained using Caspase-3/7 Green ReadyProbes™ to detect apoptotic cells (green). Scale bars, 500 μm. (**D**) Quantification of caspase-3/7 fluorescence intensity over time in PDOs from Patient #1. Combined treatment with cisplatin and GSK8612 induced significantly higher apoptosis than either monotherapy. Data are presented as mean ± SEM. Statistical significance in (**D**) was determined by two-way ANOVA with Tukey’s multiple comparisons test. Exact *P* values are indicated in the panel. (**E**) Schematic of the Cre-inducible genetically engineered mouse model expressing EGFR L858R/T790M and luciferase-tdTomato reporter, allowing conditional activation of mEGFR-driven lung tumors upon Cre recombination. (**F**) Representative hematoxylin and eosin (H&E) staining of lung sections from mice treated with vehicle, GSK8612, cisplatin, or their combination. Scale bars, 100 μm. (**G**, **H**) Quantification of tumor burden in murine lungs. Combined treatment with cisplatin and GSK8612 significantly reduced tumor area relative to total lung area (**G**) and decreased tumor number per lung section (**H**), compared with either monotherapy. Data are shown as mean ± SEM (*n* = 8). (**I**) Proposed working model: mutant EGFR (mEGFR) engages and tyrosine-phosphorylates STING and TBK1, associated with STING signalosomes and sustaining TBK1 activation, which promotes chemoresistance and tumor survival. Therapeutically, co-targeting TBK1 with chemotherapy synergistically suppresses EGFR-driven NSCLC. A question mark is included to indicate that direct complex formation between mEGFR, STING, and TBK1 is not established by the current data and may involve indirect association. Data in (**A**, **G**, **H**) are presented as mean ± SEM, and statistical significance was determined by one-way ANOVA with Tukey’s multiple comparisons test. Exact *P* values in (**A**, **G**, **H**) are indicated in the respective panels. Unless otherwise indicated, *n*  =  3 independent biological experiments. Source data are available online for this figure.

Next, we explored a synergistic treatment of GSK8612 and cisplatin in patient-derived organoids (PDO) and spontaneous murine NSCLC models. Organoids derived from EGFR-mutant patients who had developed resistance to third-generation TKIs or from wild-type EGFR patients were established and evaluated for synergistic drug effects. Cell viability assays indicated that the combination of cisplatin and TBK1 inhibitor GSK8612 significantly reduced the viability of mEGFR PDOs but not PDO bearing wild-type EGFR (Fig. 7A). A substantial synergistic effect between cisplatin and GSK8612 was confirmed by Combination Index (CI) analysis (Fig. EV5F). Dose–response curves confirmed the enhanced cisplatin sensitivity in mEGFR-PDOs by GSK8612 (Fig. 7B), suggesting a specific synthetic lethal interaction between TBK1 inhibition and cisplatin in EGFR-mutant NSCLC. The real-time apoptotic response was measured in PDOs by caspase green apoptosis probe (CGAP) assays, monitoring a rapid and substantially increased caspase activity in organoids treated with the combination of cisplatin and GSK8612, compared to the monotherapy (Figs. 7C,D and EV5G), suggesting a synergistic effect in suppressing mEGFR-driven and TKI-resistant NSCLC carcinoma by cisplatin and GSK8612.

mEGFR-driven lung carcinogenesis in an immunocompetent and genetically engineered murine model was next employed, in which Rosa26^mEGFR^ transgenic mice developed spontaneous tumors upon Adeno-Cre virus instillation (Fig. 7E). Since most advanced NSCLC patients often developed p53 loss, these mice were subjected to CRISPR-LentiV2-Cre-mediated p53 knockout to expedite tumorigenesis and followed by a month-long combinational therapy regimen (Fig. EV5H). These immunocompetent mice developed mEGFR-driven carcinomas, and immunohistochemistry revealed robust intratumoral pEGFR signaling (Fig. EV5I). The combined therapy of cisplatin and GSK8612, surpassing cisplatin or GSK8612 monotherapy, markedly attenuated tumor progression, as evidenced by hematoxylin and eosin (HE) tissue staining (Fig. 7F) and the measurement of tumor size and tumor numbers (Fig. 7G,H), suggesting a critical role of TBK1 oncogenic signaling in controlling mEGFR-driven NSCLC. Therefore, exploiting this STING–TBK1 vulnerability in mEGFR-driven NSCLC can offer a promising strategy to overcome chemoresistance and suppress tumor burden in PDOs and preclinical murine NSCLC models.

## Discussion

Our study identifies a tumor cell-intrinsic mechanism by which oncogenic EGFR mutations in NSCLC exploit STING–TBK1 signaling for survival. We demonstrate that these activating EGFR mutations associate with STING signalosomes and form a self-reinforcing loop strengthened by EGFR-mediated STING phosphorylation. mEGFR noticeably modifies TBK1, inhibiting its degradation and extending its activation magnitude and duration. Crucially, TBK1 becomes indispensable for the survival of EGFR-mutant NSCLC cells and orchestrates a prosurvival signaling network involving improved DNA damage repair (Fig. 7I). Disrupting this aberrant EGFR-STING–TBK1 axis restores sensitivity to chemotherapy in both NSCLC cells and patient-derived NSCLC organoids. Therefore, this study offers a potential therapeutic vulnerability for overcoming chemoresistance in EGFR-mutant NSCLC.

### EGFR hotspot mutations associate with and facilitate cGAS–STING signaling

While EGFR mutations are well-established drivers of NSCLC progression and resistance, their interactions with cGAS–STING signaling remain largely unexplored. Emerging evidence suggests a dual role of cGAS–STING signaling in antitumor immunity (Kwon and Bakhoum, 2020; Chin et al, 2023). Chromosomal instability has been shown to drive metastasis in a STING-dependent manner, highlighting a context-dependent role of STING signaling in cancer progression (Bakhoum et al, 2018). STING activation can also sustain tumor survival by inducing IL-6-dependent pro-survival signaling in chromosomally unstable cancers (Hong et al, 2022) and by promoting a low-grade inflammatory microenvironment that restricts myeloid-derived suppressor cells (MDSCs) (Erdal et al, 2017; Liang et al, 2017). In tumors with low antigenicity, STING also fosters tumor growth by inducing indoleamine 2,3-dioxygenase (IDO)-mediated immune suppression (Lemos et al, 2016). These complexities highlight the necessity for a comprehensive understanding of the specific implications of the cGAS–STING pathway in distinct cancer contexts.

Our study uncovered an interplay between mEGFR and cGAS–STING in NSCLC cells. Specifically, mEGFR variants, including L858R/T790M, were found to associate with STING and facilitate activation of the STING–TBK1-IRF3 signaling pathway, as evidenced by increased phosphorylation of STING and TBK1 and elevated expression of interferon-stimulated genes (ISGs). Although cGAS–STING–TBK1 signaling is classically linked to type I interferon induction, their interferon, ISG, and cytokine expression profiles are cell-context-dependent in EGFR-mutant NSCLC cells, suggesting that STING–TBK1 signaling in this setting drives a broader, context-dependent transcriptional response.

A few mechanisms explain the selective recruitment of mEGFR into STING signalosomes. First, our data suggest that mEGFR-mediated tyrosine phosphorylation of STING may reinforce pre-existing association within the STING–TBK1 signaling complex, rather than initiating de novo assembly (Fig. 3K,L). Second, the elevated endocytosis rate of mEGFR compared to wild-type EGFR (Padrón et al, 2007) might increase its spatial association with STING in endosomal compartments. Immunofluorescence analyses further revealed distinct subcellular localization patterns of mEGFR and wild-type EGFR, even under basal conditions where spatial proximity facilitates interaction (Fig. EV2B). The ability of mEGFR, but not wild-type EGFR, to enhance STING signaling likely stems from its increased kinase activity. mEGFR harbors activating mutations, such as L858R and T790M, that potentiate its catalytic activity and alter its structural conformation (Roskoski, 2019), potentially exposing interaction domains critical for binding to STING and TBK1. However, acute EGF stimulation, despite robust EGFR phosphorylation and endocytic redistribution, does not further potentiate diABZI-driven TBK1/IRF3 activation and yields a redistribution pattern distinct from the punctate assemblies observed in the mEGFR context (Appendix Fig. S2A–C). These observations argue that mEGFR-STING/TBK1 coupling is not a generic consequence of ligand-induced EGFR activation, but reflects mutant-specific signaling behavior. Future investigations are required to define the structural and trafficking determinants that distinguish oncogenic EGFR mutants from ligand-activated wtEGFR.

We also note that our data show a basal association between mEGFR and STING/TBK1 is detectable by co-immunoprecipitation and spatial association analyses, whereas agonist treatment primarily makes these assemblies more prominent by inducing punctate STING/TBK1 organization. Within this framework, tyrosine phosphorylation of STING and TBK1 is likely to reinforce and stabilize pathway coupling, rather than initiating it de novo. However, the direct biochemical interaction between mEGFR and STING/TBK1 remains to be established in fully reconstituted systems using purified proteins. In addition, the detailed tyrosine-mutant analyses were performed in a HEK293T-based reconstitution system and will require further validation in endogenous EGFR-mutant NSCLC settings.

### mEGFR utilizes TBK1 kinase to support DDR-associated responses for chemoresistance

In NSCLC cells expressing mEGFR, TBK1 is markedly activated and stabilized within STING signalosomes, evidenced by enhanced phosphorylation of TBK1 at its activation sites and a prolonged half-life in mEGFR-driven cells (Fig. 4A). Our findings reveal a previously unrecognized mechanism by which mEGFR initiates and extends the lifespan of activated TBK1. Specifically, mEGFR phosphorylates TBK1 at tyrosine residues Y577 and Y677, which inhibits K48-linked polyubiquitination, the key signal for TBK1 proteasomal degradation. TBK1, the central kinase downstream of STING activation, has received substantial attention in cancer biology. Besides its recognized roles in antitumor immunity (Xiao et al, 2017; Kwon and Bakhoum, 2020), TBK1 can respond to cellular stress and contribute to survival and proliferative signals (Tooley et al, 2021; Xu et al, 2018; Cooper et al, 2017). As such, TBK1 prevents TNF-induced cell death by RIPK1 phosphorylation (Lafont et al, 2018) and serves as a synthetic lethal target in Kras-driven (Barbie et al, 2009) and VHL loss cancers (Hu et al, 2020). TBK1 facilitates tumor survival by promoting the formation of an immunosuppressive environment, which could be a potential therapeutic target (Sun et al, 2023; Zhu et al, 2019). However, the potential role of TBK1 in chemoresistance and DNA damage repair remains poorly understood, particularly in mEGFR-driven NSCLC.

We found that TBK1 plays a pivotal role in the survival of mEGFR-driven NSCLC cells, functioning as a key regulator of pro-survival signaling networks. Upon activation, TBK1 can orchestrate multiple oncogenic pathways, including PI3K-AKT/mTORC1 and ERK MAPK/RIPK1, which drive cell growth, proliferation, and survival, ultimately fostering tumor progression and chemoresistance (Ou et al, 2011; Bodur et al, 2018). Beyond its anticipated role in pro-survival signaling, our transcriptomic analysis unexpectedly reveals that TBK1 suppression impairs DDR-associated responses. TBK1 depletion significantly downregulates multiple DNA repair pathways, including base excision repair, homologous recombination, and mismatch repair (Fig. 6B,C). TBK1 depletion suppresses DNA repair programs and impairs cisplatin-induced DDR outputs, including pATM, pBRCA1, and Rad51, while increasing γH2A.X and apoptosis markers. These findings support a functional TBK1–DDR connection in mEGFR-driven NSCLC. However, the mechanisms underlying TBK1-promoted DNA damage repair are not fully understood and are likely to involve PARP9 (Wu et al, 2019b), ATM (Banerjee et al, 2021), ISG15 (Wardlaw and Petrini, 2022), and deoxyribonucleotide metabolism (Liu et al, 2021).

An additional point emerging from the RNA-seq analysis is the apparent enrichment of inflammatory gene sets after TBK1 depletion, which at first glance appears counterintuitive given the canonical role of TBK1 in STING-IRF3 signaling. Because TBK1 loss simultaneously suppresses DNA repair programs, we favor the possibility that this inflammatory signature reflects a secondary stress-associated transcriptional response caused by genome-maintenance defects, consistent with prior studies showing that STING signaling can engage broader inflammatory transcriptional programs and that DNA damage and cellular stress can trigger compensatory NF-κB-associated inflammatory signaling (McCool and Miyamoto, 2012; Abe et al, 2013; Bakhoum and Cantley, 2018). More broadly, our study focuses on a tumor cell-intrinsic function of the mEGFR-STING–TBK1 axis. In this context, the therapeutic benefit of TBK1 inhibition is explained by impairment of tumor-cell survival and DNA damage tolerance.

Therefore, TBK1 is uniquely positioned at the crossroads of antitumor immunity and cancer cell survival. TBK1 is essential for activating immune responses against pathogens and foreign nucleic acids (Sharma et al, 2003; Liu et al, 2015). However, in mEGFR-driven NSCLC, TBK1 is hijacked to sustain cancer cell survival and drive resistance to chemotherapy, transforming it from an immune mediator into an oncogenic driver. In our immunocompetent mEGFR-driven lung tumor model, we observe an immune-excluded phenotype with limited intratumoral T-cell infiltration, low PD-L1 staining in the tumor parenchyma, and reduced tumor-cell MHC-I expression (Appendix Fig. S3A,B), consistent with the poor clinical responsiveness of EGFR-mutant NSCLC to immune checkpoint blockade. Nevertheless, we cannot exclude that enhanced tumor cell death may secondarily influence antitumor immunity, nor can we rule out direct immune-cell effects of TBK1 inhibition in vivo (Appendix Fig. S4A,B). Together with recent work implicating TBK1 in immunotherapy response (Sun et al, 2023), our findings suggest that TBK1 integrates oncogenic, stress, and immune-relevant signals in a context-dependent manner.

Therefore, this study elucidates the unanticipated exploitation of STING–TBK1 signaling to contribute to therapeutic resistance in EGFR-mutated NSCLC. Beyond addressing the immediate clinical importance of overcoming EGFR-TKI resistance, these findings expand our understanding of cancer biology by revealing a paradoxical mechanism in which tumor cells co-opt antitumor immune pathways to their advantage. These findings suggest that context-dependent engagement of innate immune pathways may also be relevant to tumor evolution and therapeutic resistance. While highlighting TBK1 as a promising therapeutic target in combination with chemotherapy for mEGFR-mutated NSCLC, these findings also raise critical questions concerning the failure of STING activation to elicit antitumor immunity in this context and the potential risks of STING agonist application in mEGFR-driven NSCLC.

## Methods

**Table:** Reagents and tools table

Reagent/resource	Reference or source	Identifier or catalog number
**Experimental models**		
HEK293T	ATCC	Cat# CRL-3216
DLD1	ATCC	Cat# CCL-221
H1299	ATCC	Cat# CRL-5803
HCC827	ATCC	Cat# CRL-2868
H1975	Provided by Prof. Song Hai	N/A
Nude mice	Shanghai SLAC Laboratory Animal Co., Ltd.	N/A
C57BL/6J mice Rosa26-EGFR(L858R/T790M) *(M. musculus)*	Shanghai Biomodel Organism Science & Technology Development	N/A
**Recombinant DNA**		
pLenti-CD74-ROS1 nonmutant	Addgene	Cat# 183813
pRK5-Flag-EML4-ALK	Lab stored	N/A
gRNA	Lab stored	N/A
Cas9-2A-GFP	Lab stored	N/A
pRK5-HA-STING	Lab stored	N/A
pRK5-HA-Flag-MAVS	Lab stored	N/A
pRK5-Myc-IRF3 2SA	Lab stored	N/A
pRK5-HA-K48-Ub	Lab stored	N/A
pRK5-HA-K63-Ub	Lab stored	N/A
pIFNβ_Luc	Lab stored	N/A
p5xISRE_Luc	Lab stored	N/A
pRK5-Flag-TBK1	Lab stored	N/A
pRK5-Myc-TBK1	Lab stored	N/A
pRK5-HA-TBK1	Lab stored	N/A
pRK5-Myc-TBK1-Y406F	This study	N/A
pRK5-Myc-TBK1-Y406E	This study	N/A
pRK5-Myc-TBK1-Y577F	This study	N/A
pRK5-Myc-TBK1-Y577E	This study	N/A
pRK5-Myc-TBK1-Y677F	This study	N/A
pRK5-Myc-TBK1-Y677E	This study	N/A
pRK5-Myc-TBK1-Y(406 + 677)F	This study	N/A
pRK5-Myc-TBK1-Y(406 + 677)E	This study	N/A
pRK5-Myc-TBK1-Y(577 + 677)F	This study	N/A
pRK5-Myc-TBK1-Y(577 + 677)E	This study	N/A
pcDNA3.1-Flag-EGFR	This study	N/A
pcDNA3.1-Flag-EGFR L858R	This study	N/A
pcDNA3.1-Flag-EGFR L858R/T790M	This study	N/A
pcDNA3.1-Flag-EGFR L858R/T790M/C797S	This study	N/A
pcDNA3.1-Flag-EGFR delE746-A750	This study	N/A
Tet-pLKO.1-puro-shEGFR-1	This study	N/A
Tet-pLKO.1-puro-shEGFR-2	This study	N/A
Tet-pLKO.1-puro-shTBK1-1	This study	N/A
Tet-pLKO.1-puro-shTBK1-2	This study	N/A
Tet-pLKO.1-puro-shSTING-1	This study	N/A
Tet-pLKO.1-puro-shSTING-2	This study	N/A
pBobi-TBK1-Myc	This study	N/A
pBobi-TBK1-Y677E-Myc	This study	N/A
pBobi-TBK1-Y(577 + 677)E-Myc	This study	N/A
CMV-Flag-EGFR-Tet-On	This study	N/A
CMV-Flag-EGFR delE746-A750-Tet-On	This study	N/A
CMV-Flag-EGFR L858R/T790M-Tet-On	This study	N/A
**Antibodies**		
Anti-STING	Cell Signaling	Cat# 13647
Anti-STING (rodent preferred)	Cell Signaling	Cat# 50494
Anti-STING	Proteintech	Cat# 19851-1-AP
Anti-pSTING (S366)	Cell Signaling	Cat# 19781
Anti-TBK1	Cell Signaling	Cat# 3504
Anti-TBK1	Cell Signaling	Cat# 38066
Anti-pTBK1 (S172)	Cell Signaling	Cat# 5483
Anti-IRF3	Cell Signaling	Cat# 4302
Anti-pIRF3 (S396)	Cell Signaling	Cat# 4947
Anti-STAT1	Cell Signaling	Cat# 14994
Anti-pSTAT1 (S727)	Cell Signaling	Cat# 8826
Anti-EGFR	Cell Signaling	Cat# 4267
Anti-pEGFR (Y1068)	Cell Signaling	Cat# 3777
Anti-AKT1	Cell Signaling	Cat# 2938
Anti-pAKT1 (S473)	Cell Signaling	Cat# 4060
Anti-ERK1/2	Cell Signaling	Cat# 4695 T
Anti-pERK1/2 (T202/Y204)	Cell Signaling	Cat# 4370 T
Anti-pY100	Cell Signaling	Cat# 9411S
Anti-PARP	Cell Signaling	Cat# 9542S
Anti-cleaved PARP	Cell Signaling	Cat# 5625
Anti-K48-Ub	Cell Signaling	Cat# 8081
Anti-K63-Ub	Cell Signaling	Cat# 5621
Anti-Caspase-3	Cell Signaling	Cat# 9662S
Anti-γ-H2A.X	Cell Signaling	Cat# 9718
Anti-Rad51	Cell Signaling	Cat# 65653
Anti-ATM	Cell Signaling	Cat# 2873
Anti-pATM (S1981)	Cell Signaling	Cat# 5883
Anti-pBRCA1 (S1524)	Cell Signaling	Cat# 9009
Anti-GAPDH	Cell Signaling	Cat# 5174
Anti-Myc	Cell Signaling	Cat# 2276S
Anti-HA	Cell Signaling	Cat# 3724S
Anti-TBK1	Abcam	Cat# ab40676
Anti-IRF3	Abcam	Cat# ab68481
Anti-pIRF3 (S386)	Abcam	Cat# ab76493
Anti-EGFR	Abcam	Cat# ab52894
Anti-pY100	Abcam	Cat# ab179530
Anti-α-tubulin	Sigma	Cat# T6199
Anti-β-actin	Sigma	Cat# A5441
Anti-HA	Sigma	Cat# H9658
Anti-Flag (M2)	Sigma	Cat# F3165
Anti-ROS1	Diagbio	Cat# db15770
Anti-GM130	BD Biosciences	Cat# 610822
Anti-STING	Santa Cruz	Cat# sc-518172
Anti-Calnexin (AF18)	Santa Cruz	Cat# sc-23954
Anti-EEA1	Santa Cruz	Cat# sc-53939
Alexa Fluor 488-AffiniPure Donkey Anti-Rabbit IgG (H + L)	Jackson ImmunoResearch	Cat# 711-545-152
Alexa Fluor 594-AffiniPure Donkey Anti-Rabbit IgG (H + L)	Jackson ImmunoResearch	Cat# 711-585-152
Alexa Fluor 488-AffiniPure Goat Anti-Mouse IgG (H + L)	Jackson ImmunoResearch	Cat# 115-545-003
Alexa Fluor 594-AffiniPure Goat Anti-Mouse IgG (H + L)	Jackson ImmunoResearch	Cat# 115-585-003
**Oligonucleotides and other sequence-based reagents**		
Oligonucleotides and sgRNA/shRNA sequences	This study	Table EV3
**Chemicals, enzymes, and other reagents**		
Poly(I:C) LMW	Invivogen	Cat# tlrl-picw
poly(dA:dT)	Invivogen	Cat# tlrl-patn
HT-DNA	Sigma-Aldrich	Cat# D6898
cGAMP	Invivogen	Cat# tlrl-nacga23-02
diABZI	Selleck	Cat# S8796
Erlotinib	Selleck	Cat# S1023
Gefitinib	Selleck	Cat# S1025
WZ4002	Selleck	Cat# S1173
Osimertinib	Selleck	Cat# S7297
MK2206	Selleck	Cat# S1078
PD98059	Selleck	Cat# S1177
SH-4-54	Selleck	Cat# S7337
GSK8612	Selleck	Cat# S8872
CMC-Na	Selleck	Cat# S6703
Cycloheximide	Sigma-Aldrich	Cat# 239763-M
Doxycycline hydrochloride	Sangon Biotech	Cat# A600889
Puromycin Dihydrochloride	Yeasen	Cat# 60210ES25
G418 Sulfate (Geneticin)	Yeasen	Cat# 60220ES03
TRIeasy	Yeasen	Cat# 10606ES60
DAPI	Santa Cruz	Cat# sc-3598
EdU kit	RIBOBIO	Cat# C10310-1
Cell Counting Kit-8 (CCK-8)	Yeasen	Cat# 40203ES60
Luciferase assay kit	Promega	Cat# N1610
Annexin V-FITC Apoptosis Detection Kit	MultiSciences	Cat# AP101AVF
Polyethyleneimine	Polysciences	Cat# 24765
Q5® High-Fidelity 2X Master Mix	New England Biolabs	Cat# M0492L
KOD Hot Start DNA Polymerase	Merck Millipore	Cat# 71086
AxyPrep Multisource Total RNA Miniprep Kit	Axygen	Cat# AP-MN-MS-RNA-50
**Software**		
GraphPad Prism v8.0	GraphPad	https://www.graphpad.com/features
Fiji v2.16	ImageJ	https://imagej.net/software/fiji/
Imaris v9.9.0	Oxford Instruments	https://imaris.oxinst.com/
CompuSyn	ComboSyn, Inc.	https://www.combosyn.com/
**Other**		

### Expression plasmids, reagents, and antibodies

Expression plasmids encoding Flag-, Myc-, or HA-tagged wild-type proteins, mutants, or truncated forms of human MAVS, STING, IRF3-2SA, TBK1, IKKε, IRF3, K48-Ub, K63-Ub, and the 5xISRE_Luc reporters have been previously described (Zhang et al, 2017; Liu et al, 2017). Flag-tagged human EGFR and NSCLC-related mutants were cloned into the pcDNA3.1 mammalian expression vector. Flag-tagged truncations of human mEGFR were cloned into the mammalian expression vector pRK5. Site-directed mutagenesis of TBK1 Y406F/E, TBK1 Y577F/E, TBK1 Y677F/E, TBK1 Y(406 + 677)F/E, TBK1 Y(577 + 677)F/E, STING-Y245F/E, STING-Y314F/E, and STING-Y(245 + 314)F was generated by PCR-based cloning performed by a kit from Stratagene. EGFR, EGFR delE746-A750, and EGFR L858R/T790M were cloned into a lentiviral pCMV vector for the generation of Tet-On systems in multiple cell types. Myc-TBK1, Myc-TBK1-Y677E, and Myc-TBK1-Y(577 + 677)E were cloned into lentiviral pBobi vectors for the generation of stable H1299 and H1975 cell lines. All coding sequences were verified by DNA sequencing. Detailed information for these constructs is provided in Table EV1.

The following pharmacological reagents were purchased and used according to the manufacturers’ instructions: erlotinib, gefitinib, WZ4002, osimertinib, GSK8612, cisplatin, MK2206, PD98059, SH-4-54, diABZI, and CMC-Na (Selleck); doxycycline (Sangon Biotech), cycloheximide (CHX), and HT-DNA (Sigma); cGAMP, poly(I:C), and poly(dA:dT) (InvivoGen); and TRIeasy, puromycin, and G418 (Yeasen).

Detailed information on antibodies applied in immunoblotting, immunoprecipitation, and immunofluorescence was provided in Table EV2. The monoclonal anti-STING (50494, 1:2000), anti-TBK1 (3504, 1:3000), anti-TBK1 (38066, 1:5000), anti-pTBK1 (S172) (5483, 1:2000), anti-IRF3 (4302, 1:2000), anti-pIRF3 (S396) (4947, 1:2000), anti-STAT1 (14994, 1:1000), anti-pSTAT1 (S727) (8826, 1:1000), anti-EGFR (4267, 1:3000), anti-pEGFR (Y1068) (3777, 1:2000), anti-pEGFR (Y845) (2231, 1:2000), anti-AKT1 (2938, 1:2000), anti-pAKT1 (S473) (4060, 1:2000), anti-ERK1/2 (4695T, 1:2000), anti-pERK1/2 (T202/Y204) (4370T, 1:2000), anti-pY100 (9411S, 1:1000), anti-cleaved PARP (5625, 1:2000), anti-K48-Ub (8081, 1:2000), anti-K63-Ub (5621, 1:2000), anti-Caspase-3 (9662S, 1:2,000), anti-PARP (9542S, 1:2000), anti-γ-H2A.X (9718, 1:1000), anti-Rad51 (65653, 1:1000), anti-ATM (2873, 1:1000), anti-pATM (S1981) (5883, 1:1000), anti-pBRCA1 (S1524) (9009, 1:1000), anti-GAPDH (5174S, 1:5000), anti-Myc (2276S, 1:3000), and anti-HA (3724S, 1:5000) antibodies were purchased from Cell Signaling Technology. The anti-TBK1 (ab40676, 1:3000), anti-IRF3 (ab68481, 1:3000), anti-pIRF3 (S386) (ab76493, 1:2000), anti-EGFR (ab52894, 1:3000), and anti-pY100 (ab179530, 1:2000) were purchased from Abcam, and the anti-tubulin (T6199, 1:10,000), anti-β-actin (A5441, 1:10,000), anti-HA (H9658, 1:2000), and anti-Flag (M2) (F3165, 1:5,000) were purchased from Sigma. Anti-ROS1 (db15770, 1:1000) was purchased from Diagbio. The anti-STING (19851-1-AP, 1:300) was purchased from Proteintech. Anti-GM130 (1:1000) was purchased from BD Biosciences. The anti-STING (518172, 1:100), anti-EGFR (52894, 1:100), anti-Calnexin (23954, 1:50), anti-EEA1(53939, 1:100), anti-rabbit IgG, and anti-mouse IgG antibodies were purchased from Santa Cruz.

### Cell culture, transfection, and infection

H1299, HCC827, and H1975 cells were cultured in RPMI 1640 medium supplemented with 10% fetal bovine serum (FBS) at 37 °C in 5% CO_2_. HEK293T and DLD1 cells were maintained in DMEM medium with 10% fetal bovine serum (FBS) at 37 °C in 5% CO_2_. Inducible DLD1, H1299, and H1975 cells expressing EGFR L858R/T790M were generated by lentiviral infection using vectors harboring a Tet-On system and selected with G418 at 1500 μg ml^−1^ for DLD1 cells or 500 μg ml^−1^ for H1299 and H1975 cells for 1 week.

PEI was used for plasmid transfection, whereas Lipofectamine RNAiMAX was used for delivery of poly(dA:dT), HT-DNA, and poly(I:C), where indicated. Digitonin (Sigma) at 10 μg ml^−1^ was used to facilitate intracellular delivery of cGAMP. For lentiviral infection, the virus-containing medium was applied directly to the target cells and replaced with fresh medium containing 10% FBS after 6 h.

### Lung organoid culture

Written informed consent was obtained from all participants, and the study was approved by the Ethics Committee of Hangzhou Cancer Hospital (Approval No. HZCH-2022-016, dated September 30, 2022). Four clinical cases were included: Patient #1, EGFR L858R/T790M with resistance to almonertinib and furmonertinib; Patient #2, EGFR del19/T790M/C797S with resistance to osimertinib; Patient #3, EGFR del19 with resistance to almonertinib; Patient #4, EGFR wild-type, previously treated with pemetrexed, cisplatin, and sintilimab. Tumor samples were stored at 4 °C in preservation solution and processed within 48 h.

After removing excess tissue, samples were washed in DPBS-PS, minced to 0.5–1 mm³ in 200 µL of digestion solution I, and further digested in 4 mL of digestion solution I at 37 °C for 1 h. Digestion was stopped with 8 mL DPBS-PS, followed by centrifugation at 1500 rpm for 5 min. Pellets were resuspended in 2 mL of digestion solution II, incubated at 37 °C for 15 min, and stopped with 4 mL DPBS-PS, then centrifuged. Cells were filtered through a 100 µm strainer, centrifuged at 1200 rpm, counted, and resuspended in Matrigel at 5000–10,000 cells per 50 µL. Matrigel domes were plated in 24-well plates, solidified, and overlaid with 500 µL MasterAim® Lung Cancer Organoid Culture Medium. Organoids were cultured at 37 °C in 5% CO₂.

### Luciferase reporter assay

HEK293T cells were transfected with the indicated reporter plasmids encoding firefly luciferase, alongside the pRL-Luc encoding Renilla luciferase as an internal control, as well as the expression constructs indicated in the corresponding Results section. Briefly, after 12 h post-transfection, the cells were treated with the indicated inhibitors and lysed in Passive Lysis Buffer (Promega) 24 h after transfection. Luciferase activity was measured using the Dual-Luciferase Assay Kit (Promega), quantified with POLARstar Omega (BMG Labtech), and normalized to the internal Renilla luciferase activity.

### Quantitative RT-PCR

H1975 and HCC827 cells treated with diABZI and cisplatin were lysed, and total RNA was extracted using an RNA extraction kit (Axygen). Complementary DNA was generated using a one-step HiScript cDNA synthesis kit (Vazyme). qRT-PCR was performed using EvaGreen qPCR master mixes (Abcam) and a CFX96 real-time PCR system (Bio-Rad). Relative gene expression was calculated using the 2^^-ΔΔCt^ method, with L19 as the endogenous reference control. All primers used in this study are listed in Table EV3.

### Coimmunoprecipitation and immunoblotting

HEK293T, H1975, HCC827, and H1299 cells treated with cGAMP, diABZI, poly(I:C), poly(dA:dT), or HT-DNA, or transfected with the indicated plasmids encoding Myc-, FLAG-, or HA-tagged proteins, were lysed in a modified MLB lysis buffer (20 mM Tris-Cl, 200 mM NaCl, 10 mM NaF, 1 mM Na₃VO₄, 1% NP-40, 20 mM β-glycerophosphate, and protease inhibitor, pH 7.5) (Xu et al, 2014). Cell lysates were then subjected to immunoprecipitation using antibodies against FLAG (Sigma, F3165-5MG; 1:200), Myc (CST, 2276S; 1:200), or HA (Sigma, H9658; 1:200) for transfected proteins, or using anti-TBK1 (3504S, 1:200) or anti-pY100 (Abcam, ab179530; 1:200) antibodies for endogenous proteins. After 3–4 washes with cold MLB buffer, precipitated proteins were resolved by SDS-PAGE and subjected to immunoblotting with the indicated antibodies. Whole-cell lysates were also analyzed using SDS-PAGE to assess the protein abundance. For endogenous IP experiments using anti-EGFR, anti-TBK1, anti-Myc, or anti-phosphotyrosine antibodies, matched normal IgG IPs are included as the relevant negative controls. For epitope-tagged IP experiments in the HEK293T reconstitution system, the corresponding bait-negative conditions serve as the relevant specificity controls. Densitometric quantification of standard immunoblots was normalized to the indicated loading control (e.g., Tubulin or GAPDH), whereas signals from IP/co-IP experiments were normalized to the corresponding immunoprecipitated bait protein in the same lane.

### Immunofluorescence and microscopy

To visualize the spatial association of the induced mEGFR with endogenous STING/TBK1, DLD1 cells expressing the inducible proteins (including FLAG-tagged mEGFR or wtEGFR for 16 h) were treated as specified in the Results section, fixed in 4% paraformaldehyde, blocked in 2% BSA in PBS for 1 h, and incubated sequentially with primary antibodies (anti-FLAG, anti-STING, anti-EGFR, or anti-TBK1) and Alexa-labeled secondary antibodies (Jackson ImmunoResearch, 711-545-152, 711-585-152, 115-545-003, and 115-585-003; 1:500), with extensive washes. Slides were then mounted with Vectorshield and counterstained with DAPI. For 3D reconstruction, confocal z-stacks were acquired and rendered in Imaris (Oxford Instruments) using the indicated fluorescence channels, and representative orthogonal views or 3D renderings were exported for presentation.

Fluorescence images were analyzed in Fiji. For quantification of TBK1 and STING redistribution, cytoplasmic regions of interest were defined per cell, and fluorescence heterogeneity was quantified as the coefficient of variation (CV = SD/mean). Higher CV values were interpreted as a more punctate distribution. For spatial association analysis, single-cell Manders overlap coefficients were calculated in Fiji using identical analysis settings across experiments.

### In vitro kinase assay

For the in vitro kinase assay, HEK293T cells were transfected with plasmids encoding Flag-tagged mEGFR, STING, or TBK1 as indicated. Twenty-four hours after transfection, cells were lysed in modified MLB buffer. Flag-tagged proteins were immunoprecipitated using anti-Flag antibody, followed by two washes with MLB buffer and two washes with kinase assay buffer (20 mM Tris-HCl, 1 mM EGTA, 5 mM MgCl₂, 0.02% 2-mercaptoethanol, 0.03% Brij-35, 0.2 mg/mL BSA, and 20 μM ATP, pH 7.4). Immunopurified proteins from HEK293T cells were then incubated in kinase assay buffer at 30 °C for 60 min on a thermomixer with agitation. Reactions were terminated by addition of 2× SDS loading buffer, and samples were analyzed by SDS-PAGE and immunoblotting with the indicated antibodies.

### shRNA-mediated RNA interference

shRNA sequences were designed by GPP WEB PORTAL (https://portals.broadinstitute.org/gpp/public/) and listed in Table EV3. Oligonucleotides were cloned into pLKO.1 or Tet-pLKO.1 plasmid at the AgeI/EcoRI sites to generate constructs targeting mEGFR, TBK1, or STING. Lentiviral production was performed in HEK293T cells by co-transfection with packaging plasmid PLP1, PLP2, and VSV-G. Media containing lentivirus were collected 48 h after transfection. Infected cells were selected in RPMI 1640 medium containing 5 µg/mL puromycin for 2 days.

### CRISPR/Cas9-mediated generation of knockout cells

To generate knockout cell lines, guide RNA sequences targeting human EGFR were designed using the CRISPRdb database (https://crisprdb.org/) and cloned into a lentiviral vector. HEK293T cells were transfected with the lentiviral packaging plasmids PLP1, PLP2, and VSV-G to produce the virus. The collected viral supernatants were used to transduce target cells, which were selected 24 h post-infection using flow cytometry to isolate Cas9-GFP-positive cells. These cells were then treated with 5 µg/mL puromycin for 48 h to ensure selection, and the efficiency of gene knockout was confirmed by immunoblotting.

### Cell viability assay and FACS

To evaluate cell viability, H1975 and HCC827 cells transfected with control shRNA or shTBK1 were seeded at 3000 cells per well in 96-well plates. After attachment, cell viability was measured at the indicated time points using the CCK-8 assay. In total, 10 μL of CCK-8 solution was added to each well for the assay. The plates were then incubated for 1–2 h in the incubator, and the absorbance was measured at 450 nm using a microplate reader. Each experimental condition was performed in triplicate.

For patient-derived lung organoids, viability was evaluated using the CellTiter-Lumi™ Luminescent Cell Viability Assay. Organoids with mEGFR-driven NSCLC were seeded in 384-well plates, with ~2000 cells per well encapsulated in a matrix droplet. The following day, treatment was initiated with a range of cisplatin concentrations, starting at 100 µM and decreasing in a fourfold dilution series as indicated, combined with 5 µM of the TBK1 inhibitor GSK8612. After 72 h, 25 µL of CellTiter-Lumi™ reagent was added to each well, followed by a 2-min orbital shake and a 10-min incubation at room temperature before measurement using a multi-mode microplate reader. Dose–response curves were fitted by nonlinear regression, and statistical comparison between curves was performed using an extra sum-of-squares *F* test. Flow cytometric analysis of Annexin V-FITC/PI staining was performed on a BD FACSCalibur according to the manufacturer’s instructions.

### Caspase-3/7 green probe assay

To assess apoptosis in patient-derived lung organoids, organoids were cultured in Matrigel in 24-well plates until they reached 50–60 µm in size. Organoids were then harvested by gentle suspension in pre-cooled PBS and seeded into 96-well plates at approximately 1 × 10^4^ cells per well in 100 µL of lung cancer culture medium (Aime Medical, 10-100-010). Caspase-3/7 Green ReadyProbes™ Reagent (Thermo Fisher) was added at 2 drops per mL of medium. Treatment included cisplatin at a maximum concentration of 100 µM in a fourfold dilution series, alone or in combination with 5 µM of the TBK1 inhibitor GSK8612. Apoptotic activity was monitored over 72 h using the Incucyte® S3 Live-Cell Analysis System (Sartorius) to capture and analyze changes in green fluorescence indicative of caspase-3/7 activity.

### Colony formation assay

Colony formation assays were performed to evaluate the clonogenic capacity of non-small cell lung cancer (NSCLC) cell lines H1975 and H1299. Cells expressing inducible control shRNA or shTBK1/shSTING were seeded at a density of 3000 cells per well in six-well plates. Following seeding, cells were treated with designated drugs and/or doxycycline. The culture medium, inclusive of drugs, was replenished every three days throughout 8–12 days to maintain optimal growth conditions. The colonies were fixed with methanol and stained with 0.2% crystal violet for visualization. Colony formation was quantified as crystal violet-positive area using the ImageJ ColonyArea plugin. Technical replicates were averaged for each independent experiment. Statistical significance was determined by two-way ANOVA followed by Šídák’s multiple comparisons test.

### Histopathological analysis

For histological examination, mouse lung tissues were dissected, fixed in 4% paraformaldehyde at 4 °C for 12 h, and dehydrated in a graded ethanol series. Following dehydration, tissues were embedded in paraffin and sectioned at 10 µm thickness. Deparaffinization was achieved by xylene treatment, and rehydration was performed through descending ethanol concentrations to water. Sections were then stained with Hematoxylin and Eosin (H&E) for general histological examination. Antigen retrieval was performed using EDTA buffer with microwave heating for immunohistochemical analysis. Sections were permeabilized with 0.5% Triton-X100 and blocked with 3% BSA to reduce non-specific binding. Overnight incubation at 4 °C with the primary antibody pEGFR (CST, 1:500) was followed by incubation with secondary antibodies and DAB staining. Processed sections were imaged using an automated pathology scanner.

### Nano-liquid chromatography/tandem MS (Nano LC-MS/MS) analysis

Phoenix National Proteomics Core services performed Nano LC/tandem MS analysis for protein identification, characterization, and label-free quantification. Tryptic peptides were separated on a C18 column and analyzed by an LTQ-Orbitrap Velos mass spectrometer (Thermo Fisher Scientific). Proteins were identified using the National Center for Biotechnology Information search engine against the human or mouse RefSeq protein databases. Detailed information on the identified proteins and modifications is provided in Dataset EV1.

### RNA isolation and sequencing library preparation

Total RNA was extracted using TRIeasy according to the manufacturer’s instructions. RNA quantity and purity were assessed using a NanoDrop ND-1000 spectrophotometer, and RNA integrity was evaluated using a Bioanalyzer 2100. Samples with RNA concentration >50 ng/μL, RIN > 7.0, and total RNA > 1 μg were used for library preparation. Poly(A)+ mRNA was enriched using oligo(dT) magnetic beads, fragmented, and reverse-transcribed to generate first-strand cDNA, followed by second-strand synthesis with incorporation of dUTP for strand specificity. After end repair, adapter ligation, size selection, and PCR amplification, libraries with an average insert size of 300 ± 50 bp were sequenced in paired-end mode on an Illumina NovaSeq 6000 platform (LC Bio-Technology Co., Ltd., Hangzhou, China). Raw reads were aligned to the human genome (hg19) using TopHat v2.1.1, and gene expression was quantified as FPKM using Cufflinks v2.2.1. Genes with FPKM < 1 in all samples were excluded. KEGG and GSEA analyses were performed based on the RNA-seq dataset.

### Subcutaneous xenograft tumor model in nude mice

Nude mice were purchased from SLAC Laboratory Animal. H1975 cells (2 × 10⁶ viable cells) were resuspended in 50 μL of fresh RPMI 1640 medium and mixed with 50 μL of YEASEN Matrix. This cell suspension was then subcutaneously injected into six- to eight-week-old nude mice. Doxycycline (Dox) was administered in drinking water at a 1 mg/mL concentration at the indicated time points to induce the control shRNA or shTBK1 expression in nude mice.

For drug treatment, cisplatin (2.5 mg/kg body weight) was administered intraperitoneally every other day starting on day 3, while the TBK1 inhibitor GSK8612 (5 mg/kg body weight) was administered intragastrically (i.g.) evey other day starting on day 3. Tumor growth was monitored daily starting on day 7 or 8 after implantation. Tumor size was estimated from two perpendicular diameters using the standard caliper-based formula described in the Results section. According to ethical guidelines, mice were euthanized when the tumor diameter reached 15 mm. All animal procedures were approved by the Institutional Animal Care and Use Committee (IACUC) of Zhejiang University.

### In vivo study design

Rosa26^mEGFR^ transgenic mice with a C57BL/6J background were generated. Specifically, the SApolyA-CAG-LSL-EGFR(L858R-T790M)-IRES-luc-2A-tdTomato-WPRE-polyA expression cassette was precisely inserted into the Rosa26 locus via homologous recombination. Both male and female littermates were used for all the experiments. Adeno-CMV-Cre or Lenti-CRISPR-V2-Cre virus was delivered intratracheally to induce tumor formation in transgenic mice. Lungs were collected for analysis at the indicated time points.

One month after the Cre-virus infection, cisplatin (2.5 mg/kg) was administered intraperitoneally, and the TBK1 inhibitor GSK8612 (5 mg/kg) was administered intragastrically, either alone or in combination. Treatment was continued for 1 month, with cisplatin administered intraperitoneally and GSK8612 administered intragastrically every two days. All the mice were bred and maintained in a pathogen-free animal facility at the Zhejiang University laboratory animal center. All animal procedures were approved by the Committee on Animal Research at Zhejiang University and conducted in accordance with Zhejiang University guidelines.

### Statistics and reproducibility

Statistical analyses were performed using GraphPad Prism unless otherwise indicated. Quantitative data are presented as the mean ± standard error of the mean (SEM) from at least three independent experiments, unless otherwise indicated. When appropriate, statistically significant differences between multiple comparisons were analyzed using the one-way or two-way ANOVA with Bonferroni correction. Differences were considered significant at *P* < 0.05.

All properly preserved and processed samples were included in the analyses, and no samples or animals were excluded except in cases of conventional injection failure or technical damage. No statistical method was used to predetermine the sample size. Non-animal experiments were not randomized. Immunoblotting, reporter assays, and qRT-PCR experiments were independently repeated a minimum of three times to ensure reproducibility, unless otherwise stated.

## Supplementary information

Movie EV1

Table EV1

Table EV2

Table EV3

Appendix

Peer Review File

Dataset EV1

Source data Fig. 1

Source data Fig. 2

Source data Fig. 3

Source data Fig. 4

Source data Fig. 5

Source data Fig. 6

Source data Fig. 7

Appendix Figure Source Data

Figure EV1 Source Data

Figure EV2 Source Data

Figure EV3 Source Data

Figure EV4 Source Data

Figure EV5 Source Data

Expanded View Figures

## Data Availability

The RNA sequencing data generated in this study have been deposited in the European Nucleotide Archive (ENA) under accession number PRJEB109276. The mass spectrometry proteomics data generated in this study have been deposited in the ProteomeXchange Consortium via the PRIDE partner repository under the dataset identifier PXD074679. Source data are provided with this paper. All other data supporting the findings of this study are available from the corresponding author upon reasonable request. The source data of this paper are collected in the following database record: biostudies:S-SCDT-10_1038-S44318-026-00856-3.
