# Supplementary material for: Oncogenic EGFR rewires STING-TBK1 signalosomes to license DNA damage tolerance in NSCLC
Source: EMBO J. 2026 Jul 10;45(16):5759–92. doi: 10.1038/s44318-026-00856-3 (PMC13482819; doi:10.1038/s44318-026-00856-3)
Supplement: Supplementary file 1 [file 44318_2026_856_MOESM21_ESM.pdf]

Expanded View Figures

Figure EV1. EGFR hotspot mutations kinase-dependently facilitate cGAS–STING signaling.

(**A**) HEK293T cells were co-transfected with an IRF3-responsive ISRE-luciferase reporter, STING, and various EGFR constructs (L858R, L858R/T790M, L858R/T790M/C797S, or delE746-A750). Luciferase activity was measured 24 h post-transfection and presented as fold induction relative to the empty vector control. EGFR and mutant EGFR expression were confirmed by immunoblotting. (**B**) HEK293T ISRE-Luc cells were co-transfected with MAVS and the indicated EGFR mutants to evaluate their effect on MAVS-mediated IRF3 activation. Luciferase activity is shown as fold induction compared to the vector control. EGFR and mutant EGFR expression were validated by immunoblotting. (**C**) HEK293T ISRE-Luc cells were co-transfected with STING-HA and increasing amounts of EGFR L858R/T790M. Luciferase activity was quantified as a fold change relative to vector control. STING and EGFR expression were confirmed by immunoblotting. (**D**) HEK293T/5×ISRE-Luc cells were transfected with STING-HA alone or together with mEGFR-Flag, and were either left untreated or stimulated with diABZI, or co-transfected with cGAS-HA, as indicated. (**E**) Immunoblotting analysis of lysates from HEK293T cells transfected as in (**D**), confirming expression of STING-HA, mEGFR-Flag, and cGAS-HA. Tubulin is shown as a loading control. (**F**) HEK293T cells were co-transfected with an IRF3-responsive ISRE-luciferase reporter with STING-HA together with mEGFR-Flag (L858R/T790M), EML4-ALK-Flag, or CD74-ROS1-Flag, as indicated. Luciferase activity was measured 24 h post-transfection and is presented as fold activation relative to the vector control. (**G**) Immunoblotting analysis of lysates from HEK293T cells transfected as in (**F**) to confirm expression of STING-HA and the indicated oncogenic kinase constructs. pY100 was used as a readout of tyrosine phosphorylation. (**H**) H1975 NSCLC cells were treated with the STING agonist cGAMP (1 h), the dsRNA analog poly(I:C) (4 h), or vehicle, in the presence or absence of erlotinib or WZ4002. Phosphorylation and total levels of TBK1, IRF3, and EGFR were assessed by immunoblotting. (**I**) H1975 iCas9 cells transduced with control or EGFR-targeting sgRNAs were treated with diABZI (1 h) and subjected to immunoblotting. (**J**) HCC827 cells (EGFR delE746-A750) were treated with diABZI (1 h) with or without erlotinib or gefitinib (10 μM) and analyzed by immunoblotting as indicated. (**K**) H1299 (wtEGFR) cells expressing control (shCtrl) or EGFR-targeting shRNAs (shEGFR-1, shEGFR-2) were treated with diABZI for the indicated time points and analyzed by immunoblotting as indicated. (**L**) HCC827 cells were treated with diABZI (2 h), WZ4002, or the combination. mRNA levels of *ISG15*, *IL6*, *CXCL10*, and *IFIT1* were measured by quantitative RT-PCR. Data are presented as mean ± SEM. Statistical significance was determined by one-way ANOVA with Tukey's multiple comparisons test. Exact *P* values are indicated in the panel. Data in (**A–D**, **F**) are presented as mean ± SEM, and statistical significance was determined by one-way ANOVA with Dunnett's multiple comparisons test. Exact *P* values are indicated in the respective panels, ns, no significance. Unless otherwise indicated, $n = 3$ independent biological experiments. Densitometric values are indicated below the bands and provided in the source data. Source data are available online for this figure.

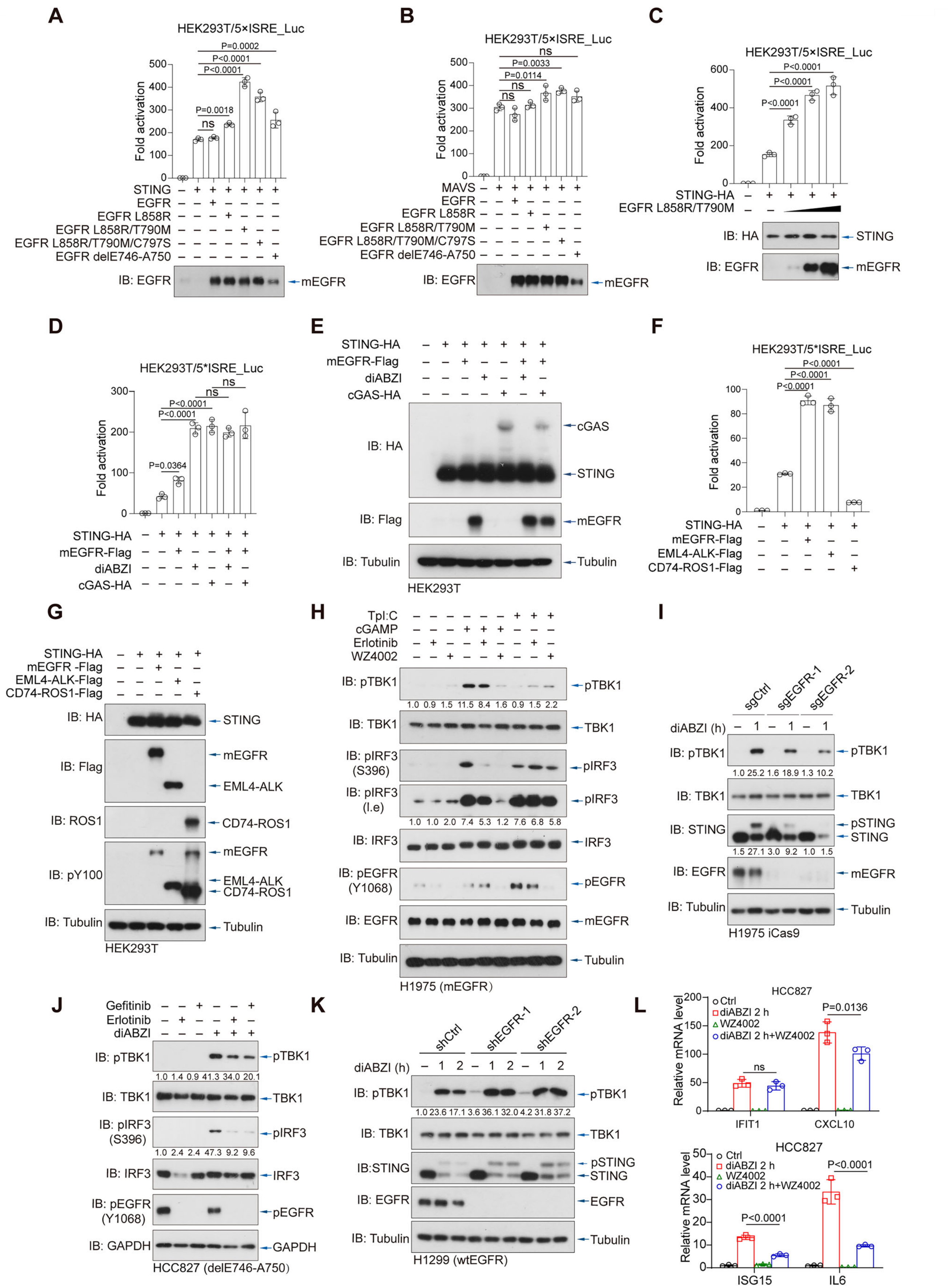

 © The Author(s)

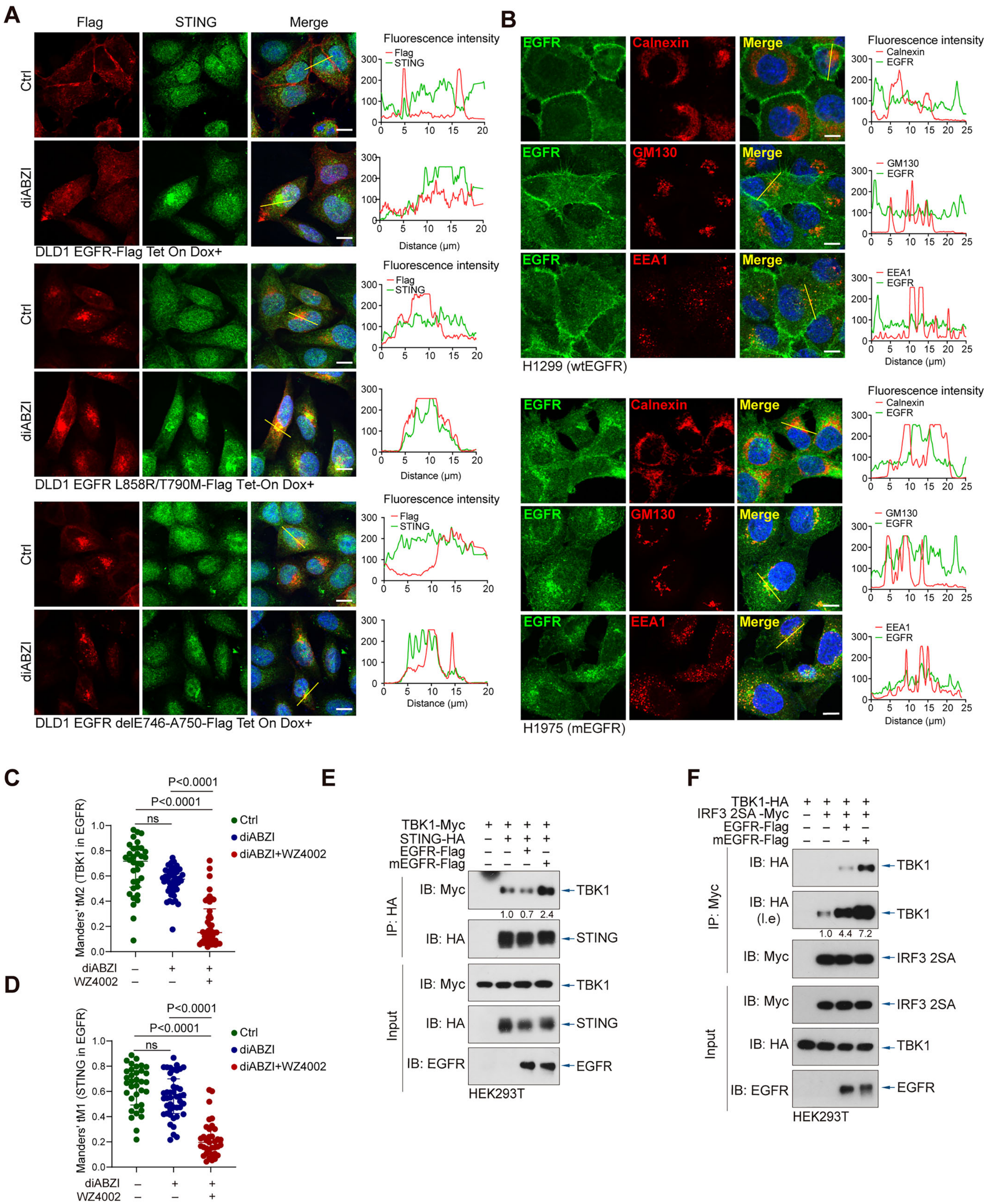
A
Flag
STING
Merge
Ctrl
diABZI
Fluorescence intensity
Flag
STING
Distance (μm)
DLD1 EGFR-Flag Tet On Dox+
DLD1 EGFR L858R/T790M-Flag Tet-On Dox+
DLD1 EGFR delE746-A750-Flag Tet On Dox+
B
EGFR
Calnexin
GM130
EEA1
Merge
H1299 (wtEGFR)
H1975 (mEGFR)
C
Manders' tM2 (TBK1 in EGFR)
Ctrl
diABZI
diABZI+WZ4002
ns
P<0.0001
P<0.0001
WZ4002
D
Manders' tM1 (STING in EGFR)
E
TBK1-Myc
STING-HA
EGFR-Flag
mEGFR-Flag
IP: HA
Input
IB: Myc
IB: HA
IB: EGFR
TBK1
STING
EGFR
1.0 0.7 2.4
HEK293T
F
TBK1-HA
IRF3 2SA -Myc
IP: Myc
IB: HA (l.e)
IRF3 2SA
1.0 4.4 7.2

Figure EV2. Mutant and wild-type EGFR differentially localize and interact with TBK1–STING signalosomes.

(**A**) DLD1 cells expressing the indicated EGFR variants were treated with diABZI (1 h) with or without WZ4002 (10 μM, 6 h), then fixed and stained with antibodies against Flag (red) and STING (green). Right, fluorescence intensity profiles along the indicated line scans illustrate co-enrichment of Flag and STING signals in clustered regions. Scale bars, 10 μm. (**B**) H1975 and H1299 cells were fixed and co-stained with antibodies against EGFR (green) and one of the following organelle markers, including calnexin (ER), GM130 (Golgi), or EEA1 (early endosomes) in red. Representative confocal images are shown. Right, fluorescence intensity profiles along the indicated line scans (yellow) in the merged images illustrate the degree of spatial overlap between EGFR and each organelle marker. Scale bars, 10 μm. (**C**, **D**) Quantification of spatial overlap in H1975 (mEGFR) cells treated with diABZI with or without WZ4002, based on Manders overlap coefficients measured from single-cell ROIs. (**C**) Manders' tM2 (TBK1 in EGFR). (**D**) Manders' tM1 (STING in EGFR). Each dot represents one cell. Data were from three independent experiments (total cell numbers: TBK1-EGFR, Ctrl $n = 34$, diABZI $n = 43$, diABZI + WZ4002 $n = 46$; STING-EGFR, Ctrl $n = 35$, diABZI $n = 43$, diABZI + WZ4002 $n = 35$). Data in (**C**, **D**) are presented as mean ± SEM and statistical significance was determined by the Kruskal–Wallis test with Dunn's multiple comparisons. Exact *P* values are indicated in the respective panels; ns, no significance. (**E**) HEK293T cells were co-transfected with Flag-tagged wild-type EGFR or mEGFR, HA-tagged STING, and Myc-tagged TBK1. Cell lysates were immunoprecipitated with anti-HA and analyzed by immunoblotting with anti-Myc, anti-HA, and anti-EGFR antibodies. (**F**) HEK293T cells were co-transfected with Flag-tagged wild-type EGFR or mEGFR, Myc-tagged IRF3 2SA mutant (S385A/S386A), and HA-tagged TBK1. Myc immunoprecipitates were analyzed by immunoblotting. l.e., longer exposure. Data information: unless otherwise indicated, $n = 3$ independent biological experiments. Densitometric values are indicated below the bands and provided in the source data. Source data are available online for this figure.

 © The Author(s)

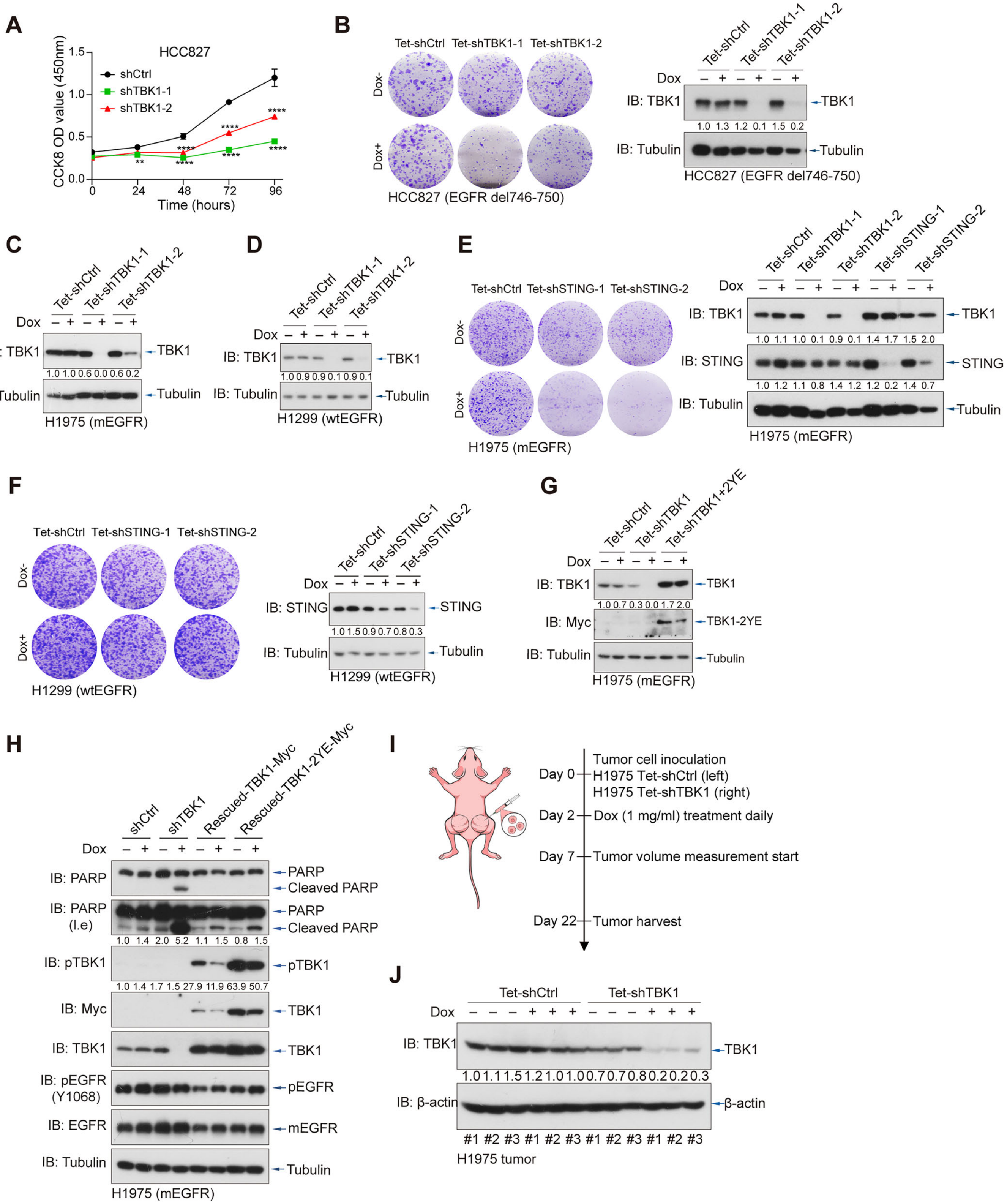
A
HCC827
CCK8 OD value (450nm)
shCtrl
shTBK1-1
shTBK1-2
Time (hours)
0 24 48 72 96
0.0 0.5 1.0 1.5
B
Tet-shCtrl Tet-shTBK1-1 Tet-shTBK1-2
Dox-
Dox+
HCC827 (EGFR del746-750)
Dox
IB: TBK1
TBK1
1.0 1.3 1.2 0.1 1.5 0.2
IB: Tubulin
Tubulin
HCC827 (EGFR del746-750)
C
Tet-shCtrl Tet-shTBK1-1 Tet-shTBK1-2
Dox
IB: TBK1
TBK1
1.0 1.0 0.6 0.0 0.6 0.2
IB: Tubulin
Tubulin
H1975 (mEGFR)
D
Tet-shCtrl Tet-shTBK1-1 Tet-shTBK1-2
Dox
IB: TBK1
TBK1
1.0 0.9 0.9 0.1 0.9 0.1
IB: Tubulin
Tubulin
H1299 (wtEGFR)
E
Tet-shCtrl Tet-shSTING-1 Tet-shSTING-2
Dox-
Dox+
H1975 (mEGFR)
Tet-shCtrl Tet-shTBK1-1 Tet-shTBK1-2 Tet-shSTING-1 Tet-shSTING-2
Dox
IB: TBK1
TBK1
1.0 1.1 1.0 0.1 0.9 0.1 1.4 1.7 1.5 2.0
IB: STING
STING
1.0 1.2 1.1 0.8 1.4 1.2 1.2 0.2 1.4 0.7
IB: Tubulin
Tubulin
H1975 (mEGFR)
F
Tet-shCtrl Tet-shSTING-1 Tet-shSTING-2
Dox-
Dox+
H1299 (wtEGFR)
Tet-shCtrl Tet-shSTING-1 Tet-shSTING-2
Dox
IB: STING
STING
1.0 1.5 0.9 0.7 0.8 0.3
IB: Tubulin
Tubulin
H1299 (wtEGFR)
G
Tet-shCtrl Tet-shTBK1 Tet-shTBK1+2YE
Dox
IB: TBK1
TBK1
1.0 0.7 0.3 0.0 1.7 2.0
IB: Myc
TBK1-2YE
IB: Tubulin
Tubulin
H1975 (mEGFR)
H
shCtrl shTBK1 Rescued-TBK1-Myc Rescued-TBK1-2YE-Myc
Dox
IB: PARP
PARP
Cleaved PARP
IB: PARP (l.e)
PARP
Cleaved PARP
1.0 1.4 2.0 5.2 1.1 1.5 0.8 1.5
IB: pTBK1
pTBK1
1.0 1.4 1.7 1.5 27.9 11.9 63.9 50.7
IB: Myc
TBK1
IB: TBK1
TBK1
IB: pEGFR (Y1068)
pEGFR
IB: EGFR
mEGFR
IB: Tubulin
Tubulin
H1975 (mEGFR)
I
Day 0 Tumor cell inoculation
H1975 Tet-shCtrl (left)
H1975 Tet-shTBK1 (right)
Day 2 Dox (1 mg/ml) treatment daily
Day 7 Tumor volume measurement start
Day 22 Tumor harvest
J
Tet-shCtrl Tet-shTBK1
Dox
IB: TBK1
TBK1
1.0 1.1 1.5 1.2 1.0 1.0 0.7 0.7 0.8 0.2 0.2 0.3
IB: β-actin
β-actin
#1 #2 #3 #1 #2 #3 #1 #2 #3 #1 #2 #3
H1975 tumor

Figure EV3. STING and TBK1 are indispensable for mEGFR-driven NSCLC survival.

(**A**) CCK-8 assays measured cell viability in mutant EGFR-driven HCC827 NSCLC cells with shCtrl or shTBK1. Data are presented as mean ± SEM. Statistical significance was determined by two-way ANOVA with Šídák's multiple comparisons test. $^{**}P < 0.01$, and $^{****}P < 0.0001$. Exact P values are provided in the source data. (**B**) Colony formation assays of HCC827 cells expressing inducible shCtrl or two independent shTBK1 constructs, with or without Dox treatment. (**C**, **D**) Knockdown efficiency of TBK1 in H1975 (**C**) and H1299 (**D**) was confirmed by immunoblotting. (**E**, **F**) Colony formation assays were performed as in Fig. 5E in doxycycline-inducible shRNA cell lines targeting STING in H1975 (**E**) and H1299 (**F**) cells. Two independent shRNAs were used, and knockdown efficiency was confirmed by immunoblotting (right). (**G**) Knockdown and rescue efficiency were confirmed by immunoblotting in H1975 cells. (**H**) Doxycycline-inducible TBK1 knockdown H1975 (mEGFR) cells were reconstituted with an shRNA-resistant TBK1-Myc (WT) or TBK1-2YE-Myc rescue construct, as indicated. Whole-cell lysates were analyzed by immunoblotting to assess PARP cleavage and TBK1 expression. (**I**) Schematic illustration of the in vivo experimental timeline for H1975 xenograft implantation and doxycycline treatment. (**J**) Immunoblotting analysis of TBK1 protein levels in individual H1975 tumor samples ($n = 3$ per group) with or without doxycycline treatment to induce shTBK1 expression. Representative colony formation images and corresponding immunoblots from two independent experiments with similar results are shown. Data information: unless otherwise indicated, $n = 3$ independent biological experiments. Densitometric values are indicated below the bands and provided in the source data. Source data are available online for this figure.

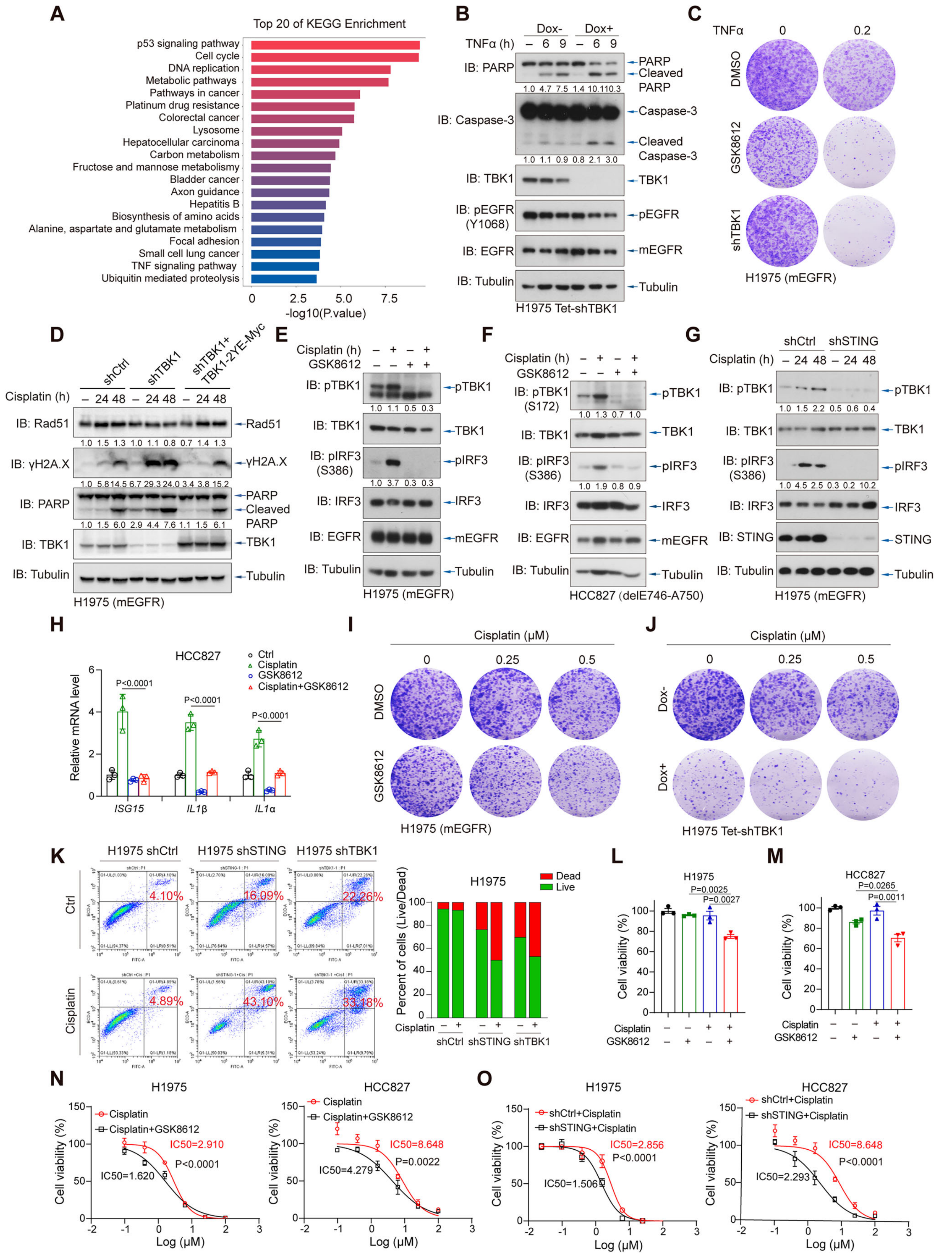
A
Top 20 of KEGG Enrichment
p53 signaling pathway
Cell cycle
DNA replication
Metabolic pathways
Pathways in cancer
Platinum drug resistance
Colorectal cancer
Lysosome
Hepatocellular carcinoma
Carbon metabolism
Fructose and mannose metabolismy
Bladder cancer
Axon guidance
Hepatitis B
Biosynthesis of amino acids
Alanine, aspartate and glutamate metabolism
Focal adhesion
Small cell lung cancer
TNF signaling pathway
Ubiquitin mediated proteolysis
0 2.5 5.0 7.5
-log10(P.value)
B
Dox- Dox+
TNFα (h) – 6 9 – 6 9
IB: PARP
PARP
Cleaved PARP
1.0 4.7 7.5 1.4 10.1 10.3
IB: Caspase-3
Caspase-3
Cleaved Caspase-3
1.0 1.1 0.9 0.8 2.1 3.0
IB: TBK1
TBK1
IB: pEGFR (Y1068)
pEGFR
IB: EGFR
mEGFR
IB: Tubulin
Tubulin
H1975 Tet-shTBK1
C
TNFα 0 0.2
DMSO
GSK8612
shTBK1
H1975 (mEGFR)
D
shCtrl
shTBK1
shTBK1+ TBK1-2YE-Myc
Cisplatin (h) – 24 48 – 24 48 – 24 48
IB: Rad51
Rad51
1.0 1.5 1.3 1.0 1.1 0.8 0.7 1.4 1.3
IB: γH2A.X
γH2A.X
1.0 5.8 14.5 6.7 29.3 24.0 3.4 3.8 15.2
IB: PARP
PARP
Cleaved PARP
1.0 1.5 6.0 2.9 4.4 7.6 1.1 1.5 6.1
IB: TBK1
TBK1
IB: Tubulin
Tubulin
H1975 (mEGFR)
E
Cisplatin (h) – + – +
GSK8612 – – + +
IB: pTBK1
pTBK1
1.0 1.1 0.5 0.3
IB: TBK1
TBK1
IB: pIRF3 (S386)
pIRF3
1.0 3.7 0.3 0.3
IB: IRF3
IRF3
IB: EGFR
mEGFR
IB: Tubulin
Tubulin
H1975 (mEGFR)
F
Cisplatin (h) – + – +
GSK8612 – – + +
IB: pTBK1 (S172)
pTBK1
1.0 1.3 0.7 1.0
IB: TBK1
TBK1
IB: pIRF3 (S386)
pIRF3
1.0 1.9 0.8 0.9
IB: IRF3
IRF3
IB: EGFR
mEGFR
IB: Tubulin
Tubulin
HCC827 (delE746-A750)
G
shCtrl shSTING
Cisplatin (h) – 24 48 – 24 48
IB: pTBK1
pTBK1
1.0 1.5 2.2 0.5 0.6 0.4
IB: TBK1
TBK1
IB: pIRF3 (S386)
pIRF3
1.0 4.5 2.5 0.3 0.2 10.2
IB: IRF3
IRF3
IB: STING
STING
IB: Tubulin
Tubulin
H1975 (mEGFR)
H
HCC827
Ctrl
Cisplatin
GSK8612
Cisplatin+GSK8612
Relative mRNA level
P<0.0001
P<0.0001
P<0.0001
ISG15
IL1β
IL1α
I
Cisplatin (μM)
0 0.25 0.5
DMSO
GSK8612
H1975 (mEGFR)
J
Cisplatin (μM)
0 0.25 0.5
Dox-
Dox+
H1975 Tet-shTBK1
K
H1975 shCtrl
H1975 shSTING
H1975 shTBK1
Ctrl
Cisplatin
4.10%
16.09%
22.26%
4.89%
43.10%
33.18%
H1975
Dead
Live
Percent of cells (Live/Dead)
Cisplatin – + – + – +
shCtrl shSTING shTBK1
L
H1975
P=0.0025
P=0.0027
Cell viability (%)
Cisplatin – – + +
GSK8612 – + – +
M
HCC827
P=0.0265
P=0.0011
Cell viability (%)
Cisplatin – – + +
GSK8612 – + – +
N
H1975
Cisplatin
Cisplatin+GSK8612
IC50=2.910
P<0.0001
IC50=1.620
Cell viability (%)
Log (μM)
HCC827
Cisplatin
Cisplatin+GSK8612
IC50=8.648
P=0.0022
IC50=4.279
Cell viability (%)
Log (μM)
O
H1975
shCtrl+Cisplatin
shSTING+Cisplatin
IC50=2.856
P<0.0001
IC50=1.506
Cell viability (%)
Log (μM)
HCC827
shCtrl+Cisplatin
shSTING+Cisplatin
IC50=8.648
P<0.0001
IC50=2.293
Cell viability (%)
Log (μM)

Figure EV4. TBK1 supports DDR-associated responses and survival in mEGFR-driven NSCLC.

(**A**) Transcriptomic analyses of KEGG-enriched pathways in H1975 cells with TBK1 depletion indicated suppression of oncogenic and survival pathways. *Y* axis: enriched pathways; *X* axis: enrichment scores; color intensity denotes *P* value significance. Enrichment significance was assessed using Fisher's exact test. (**B**) Immunoblotting of H1975 Tet-shTBK1 cells treated with TNFα in the presence or absence of Dox for the indicated time points. TBK1 depletion exacerbated TNFα-induced apoptosis, as evidenced by increased levels of cleaved PARP and cleaved caspase-3. (**C**) Colony formation assays in H1975 cells treated with TNFα and DMSO, GSK8612, or TBK1 shRNA. Both pharmacological inhibition and genetic depletion of TBK1 sensitized cells to TNFα-induced growth suppression. (**D**) H1975 cells expressing shCtrl or shTBK1, or shTBK1 together with shRNA-resistant TBK1-2YE–Myc were treated with cisplatin (8 μM) for 24 h or 48 h, followed by immunoblotting analysis as indicated. (**E**, **F**) Immunoblotting of H1975 (**E**) and HCC827 (**F**) cells treated with cisplatin (8 μM, 48 h), with or without GSK8612 (5 μM), to assess TBK1-IRF3 pathway activation. (**G**) Immunoblotting analysis of H1975 cells expressing shCtrl or shSTING and treated with cisplatin (8 μM) for the indicated time points, showing STING-dependent activation of TBK1 signaling upon cisplatin treatment. (**H**) Quantitative RT-PCR analysis of interferon-stimulated genes (*ISG15*, *IL1β*, and *IL1α*) in HCC827 cells treated with cisplatin (8 μM), GSK8612 (5 μM), or the combination. TBK1 inhibition suppressed cisplatin-induced ISG expression. (**I**) Colony formation assays in H1975 (mEGFR) cells treated with cisplatin at the indicated doses in the presence of DMSO or GSK8612 (5 μM). (**J**) Colony formation assays in H1975 Tet-shTBK1 cells treated with cisplatin at the indicated doses, with Dox-/Dox+ to control TBK1 knockdown. (**K**) Flow cytometry analysis of Annexin V/PI-stained H1975 cells expressing shCtrl, shSTING, or shTBK1, treated with cisplatin (1 μM, 48 h). Right: Quantification of live (Annexin V−/PI−) and dead (Annexin V+ and/or PI +) cell fractions. (**L**, **M**) CCK-8 assays measuring viability of H1975 (**L**) and HCC827 (**M**) cells treated with cisplatin (0.4 μM) and/or GSK8612 (5 μM). For bar graphs in (**L**, **M**), values were extracted from the indicated dose points in the corresponding dose–response experiments shown in (**N**). (**N**) Dose–response curves of cisplatin alone or combined with GSK8612 in H1975 and HCC827 cells. Co-treatment significantly decreased cisplatin IC50, suggesting enhanced sensitivity. (**O**) Dose–response curves of cisplatin in H1975 and HCC827 cells expressing control or STING-targeting shRNAs. Data in (**N**, **O**) are presented as mean ± SEM. Differences in logIC50 values were assessed by an extra sum-of-squares *F* test. Exact *P* values for (**N**, **O**) are indicated in the panels. Data in (**H**, **L**, **M**) are presented as mean ± SEM. Statistical significance in (**H**, **L**, **M**) was determined by one-way ANOVA with Tukey's multiple comparisons test. Exact *P* values in (**H**, **L**, **M**) are indicated in the respective panels. Representative colony formation images in (**C**, **I**, **J**) are shown from two independent experiments with similar results. Unless otherwise indicated, $n = 3$ independent biological experiments. Densitometric values are indicated below the bands and provided in the source data. Source data are available online for this figure.

 © The Author(s)

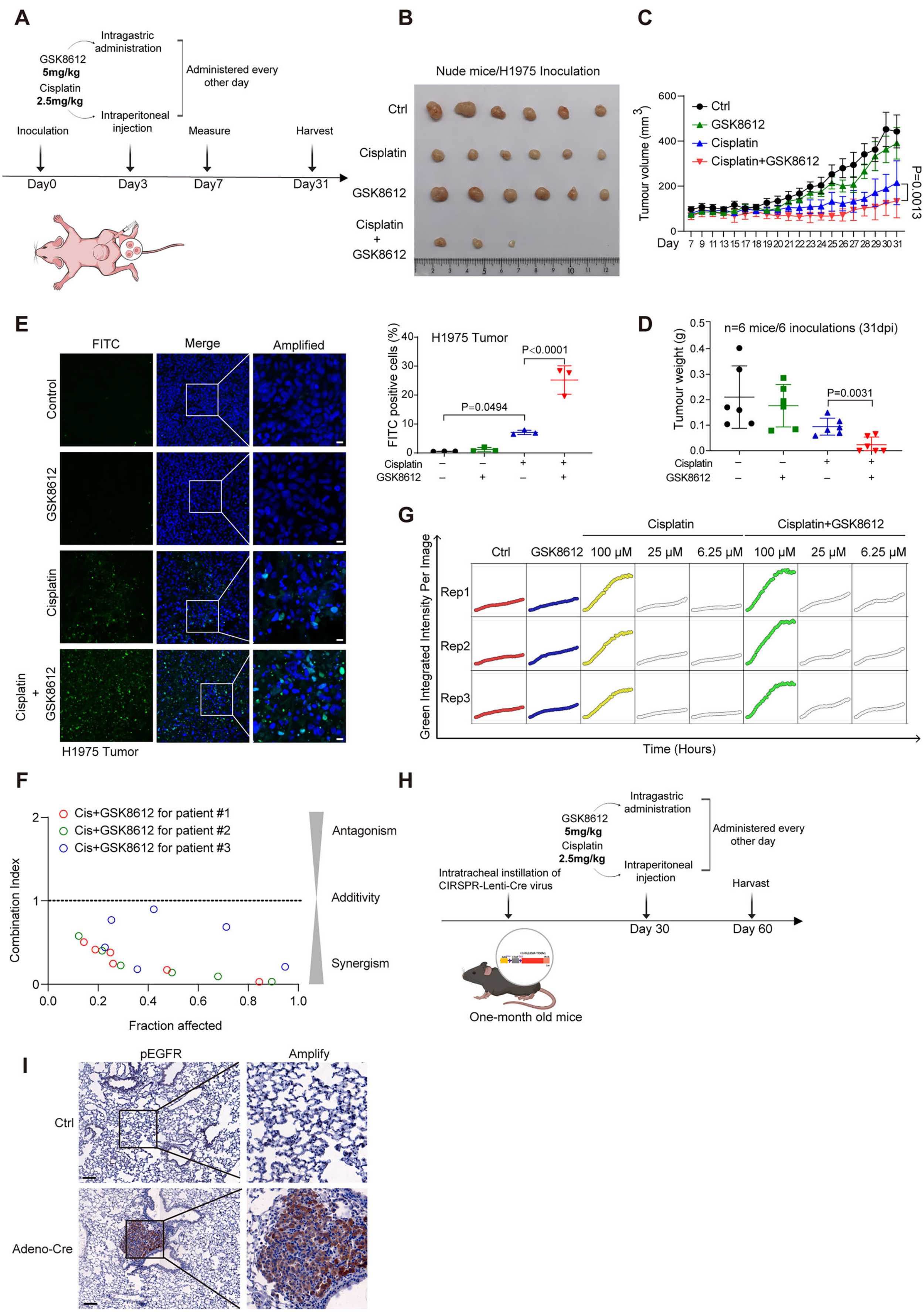
A
Intragastric administration
GSK8612 5mg/kg
Cisplatin 2.5mg/kg
Intraperitoneal injection
Administered every other day
Inoculation
Measure
Harvest
Day0
Day3
Day7
Day31
B
Nude mice/H1975 Inoculation
Ctrl
Cisplatin
GSK8612
Cisplatin + GSK8612
C
Ctrl
GSK8612
Cisplatin
Cisplatin+GSK8612
Tumour volume (mm3)
Day
P=0.0013
E
FITC
Merge
Amplified
Control
GSK8612
Cisplatin
Cisplatin + GSK8612
H1975 Tumor
FITC positive cells (%)
P<0.0001
P=0.0494
Cisplatin
GSK8612
D
n=6 mice/6 inoculations (31dpi)
Tumour weight (g)
P=0.0031
G
Cisplatin
Cisplatin+GSK8612
Ctrl
GSK8612
100 μM
25 μM
6.25 μM
Rep1
Rep2
Rep3
Green Integrated Intensity Per Image
Time (Hours)
F
Cis+GSK8612 for patient #1
Cis+GSK8612 for patient #2
Cis+GSK8612 for patient #3
Combination Index
Fraction affected
Antagonism
Additivity
Synergism
H
Intratracheal instillation of CIRSPR-Lenti-Cre virus
Day 30
Day 60
One-month old mice
I
pEGFR
Amplify
Ctrl
Adeno-Cre

Figure EV5. Combining cisplatin and TBK1i diminishes mEGFR-driven NSCLC PDOs and spontaneous tumors.

(**A**) Schematic of the experimental design for the H1975 xenograft model. Nude mice were subcutaneously inoculated with H1975 NSCLC cells and treated every other day with GSK8612 (5 mg/kg, intragastric) and/or cisplatin (2.5 mg/kg, intraperitoneal), starting on day 3 post-inoculation. (**B**) Representative images of excised tumors at day 31 post-inoculation (dpi) ($n = 6$ mice per group). Mice receiving combination therapy (cisplatin + GSK8612) exhibited the most significant tumor suppression. (**C**, **D**) Quantification of tumor progression in xenografts. Tumor volume was measured at the indicated time points (**C**), and tumor weight was assessed at 31 dpi (**D**). Combination treatment significantly reduced both parameters compared to monotherapies ($n = 6$ mice per group). (**E**) TUNEL staining of xenograft tumor tissues showed markedly increased apoptosis in the combination group, as indicated by elevated FITC-positive cells (green). Scale bars, 10 μm. Quantification shown on the right. (**F**) Drug interaction analysis using CompuSyn software demonstrated synergism (combination index, CI < 1) between cisplatin and GSK8612 across a range of concentrations in EGFR-mutant NSCLC PDOs from three patients. (**G**) Quantification of caspase-3/7 activation over time in PDOs (Patient #1) treated with cisplatin (at indicated concentrations) and GSK8612 (5 μM). Data are shown from three biological replicates using a live-cell apoptosis detection assay. (**H**) Schematic of the treatment schedule in the spontaneous mEGFR NSCLC mouse model. One-month-old mice were intratracheally instilled with CRISPR-Lenti-Cre virus to induce lung-specific expression of EGFR L858R/T790M. On day 30, mice received GSK8612 and/or cisplatin every other day until sacrifice on day 60. (**I**) Immunohistochemistry (IHC) of lung tissues from control or Adeno-Cre-infected mice revealed strong pEGFR expression in tumor regions of the Cre-treated group. Scale bars, 100 μm. Data in (**C–E**) are presented as mean ± SEM. Statistical significance in (**C**) was determined by two-way ANOVA, and (**D**, **E**) were determined by one-way ANOVA with Tukey's multiple comparisons test. Exact *P* values in (**C–E**) are indicated in the corresponding panel. Unless otherwise indicated, $n = 3$ independent biological experiments. Source data are available online for this figure.

© The Author(s)
