# Supplementary material for: Oncogenic EGFR rewires STING-TBK1 signalosomes to license DNA damage tolerance in NSCLC
Source: EMBO J. 2026 Jul 10;45(16):5759–92. doi: 10.1038/s44318-026-00856-3 (PMC13482819; doi:10.1038/s44318-026-00856-3)
Supplement: Supplementary file 2 [file 44318_2026_856_MOESM5_ESM.pdf]

Table of contents

Appendix – Table of Contents

Appendix Figure S1: mEGFR stabilizes the activated TBK1 1

Appendix Figure S2: EGF does not enhance diABZI-stimulated STING signaling 2

Appendix Figure S3: Immune-excluded phenotype and low MHC-I in mEGFR tumors 3

Appendix Figure S4: PDO immune subset profiling under TBK1 inhibition and αPD-1 4

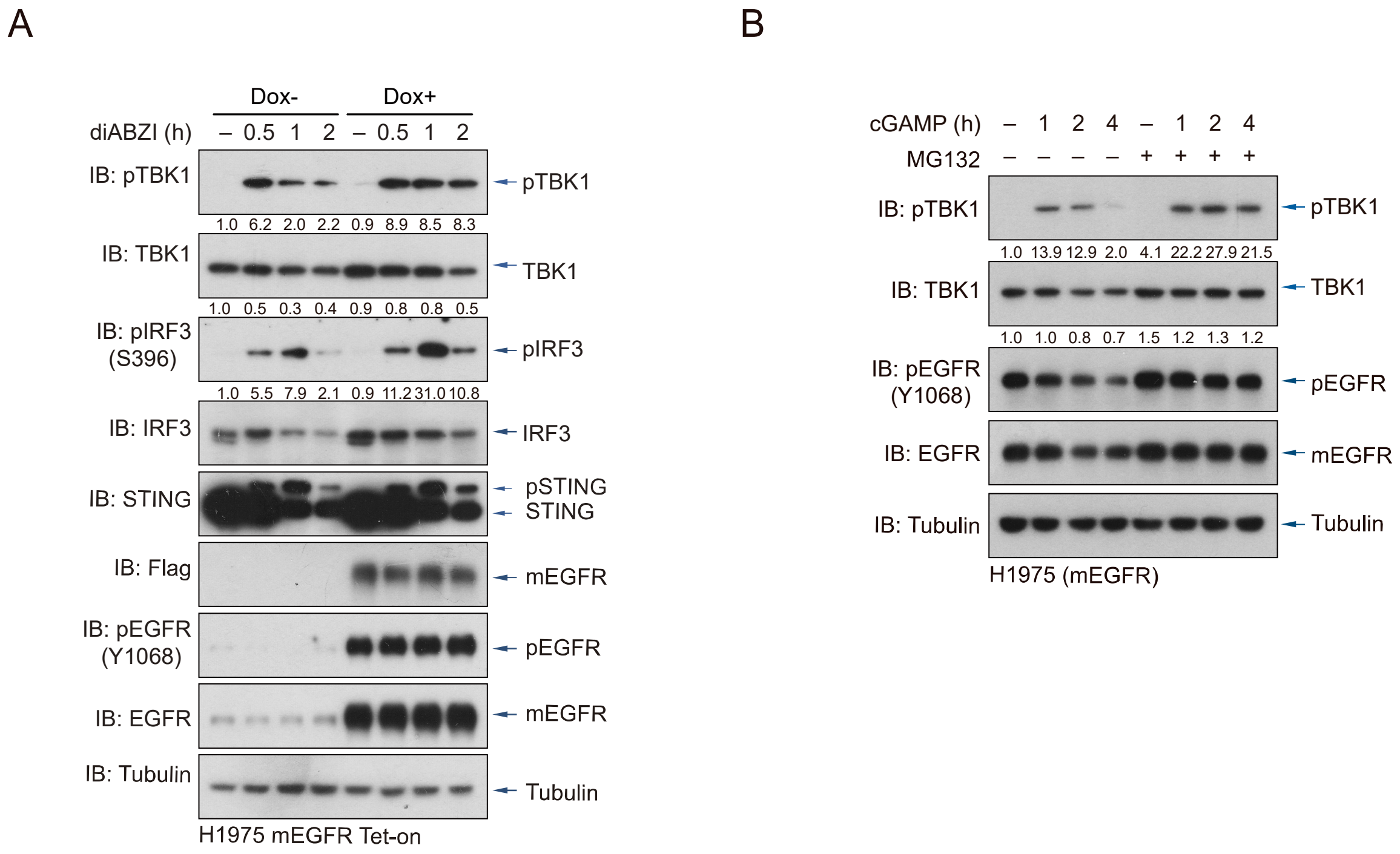

Appendix Figure S1. mEGFR attenuates the decline of activated TBK1 during STING pathway activation.

(A) H1975 mEGFR Tet-On cells were left untreated (Dox–) or induced with doxycycline (Dox+) and treated with diABZI for the indicated times, followed by immunoblotting analysis as indicated.
(B) H1975 cells were treated with cGAMP for the indicated times in the presence or absence of MG132, followed by immunoblotting analysis as indicated. Numbers indicate normalized densitometric quantification (see Materials and Methods).

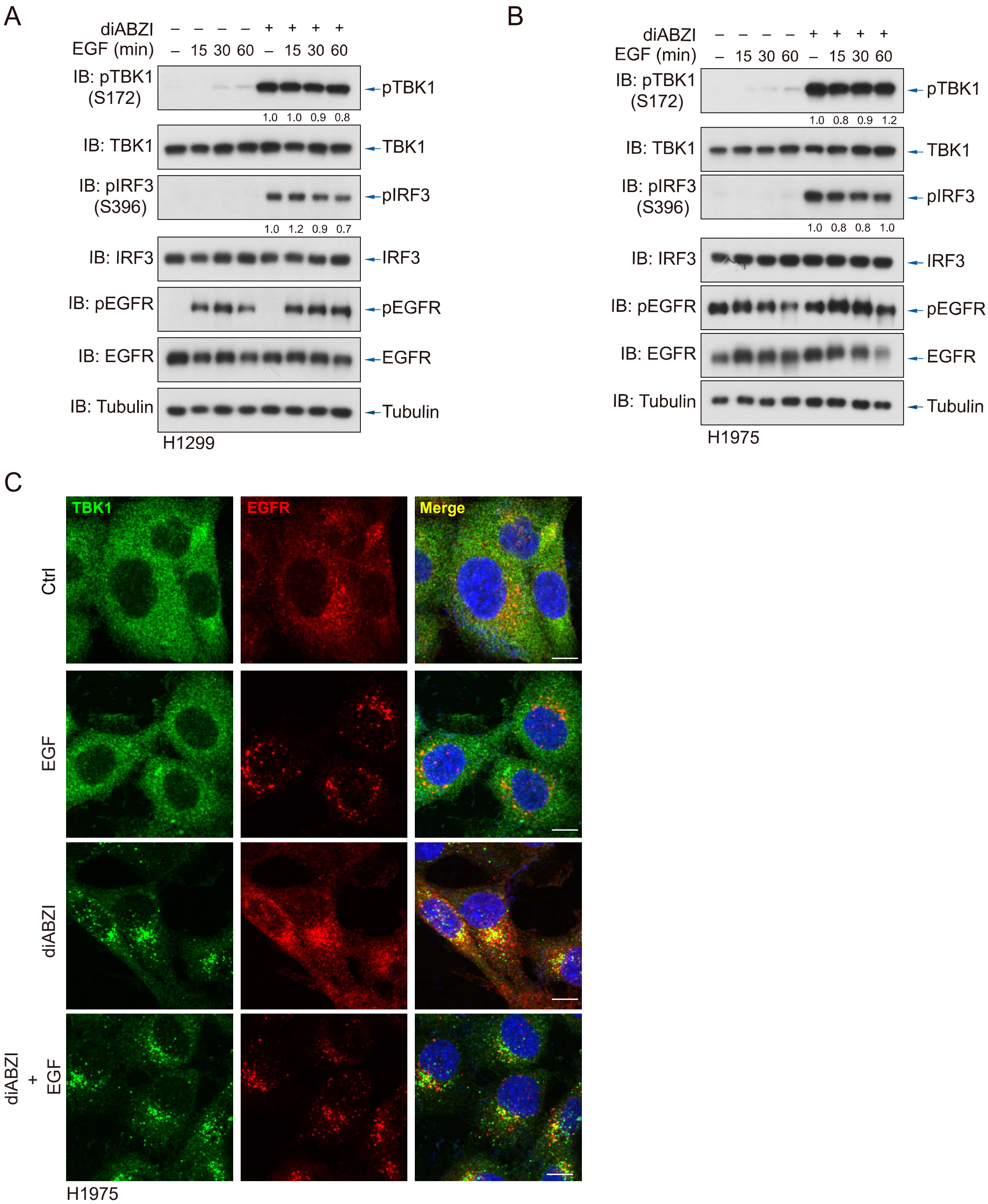

Appendix Figure S2. EGF stimulation does not potentiate diABZI-driven TBK1/IRF3 activation and induces a distinct EGFR redistribution pattern.

(A) H1299 (wtEGFR) and H1975 (mEGFR) cells were stimulated with EGF for the indicated times in the absence or presence of diABZI, followed by immunoblotting for pTBK1, pIRF3, and EGFR phosphorylation as indicated.
(B) Confocal imaging of H1975 cells stained for TBK1 and EGFR under Ctrl, EGF, diABZI, or diABZI+EGF conditions. Nuclei were counterstained with DAPI. Scale bars, 10 μm.

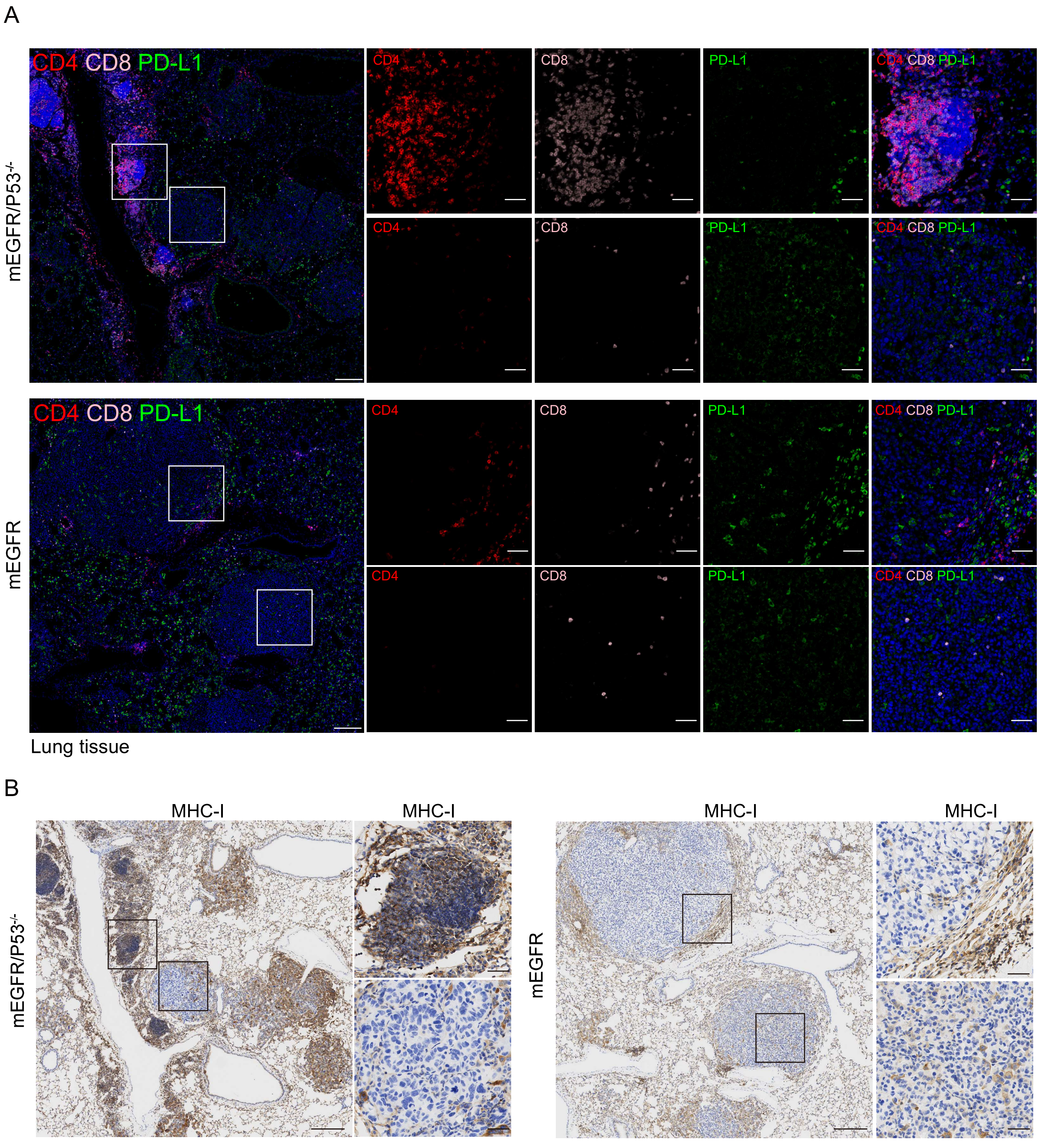

Appendix Figure S3. Immune-excluded phenotype and low MHC-I expression in an immunocompetent mEGFR-driven lung tumor model.

(A) Multiplex TSA staining of lung tissue sections showing CD4 (red), CD8 (grey/white), and PD-L1 (green), with nuclei counterstained with DAPI (blue), in mEGFR/Trp53−/− and mEGFR tumors as indicated. Boxed regions are shown at higher magnification in the adjacent panels. Scale bars: 100 µm (overview) and 50 µm (magnified views).
(B) Immunohistochemistry for MHC-I in lung tissue sections from mEGFR/Trp53−/− and mEGFR tumors as indicated. Boxed regions are shown at higher magnification. Scale bars: 50 µm (overview) and 40 µm (magnified views).

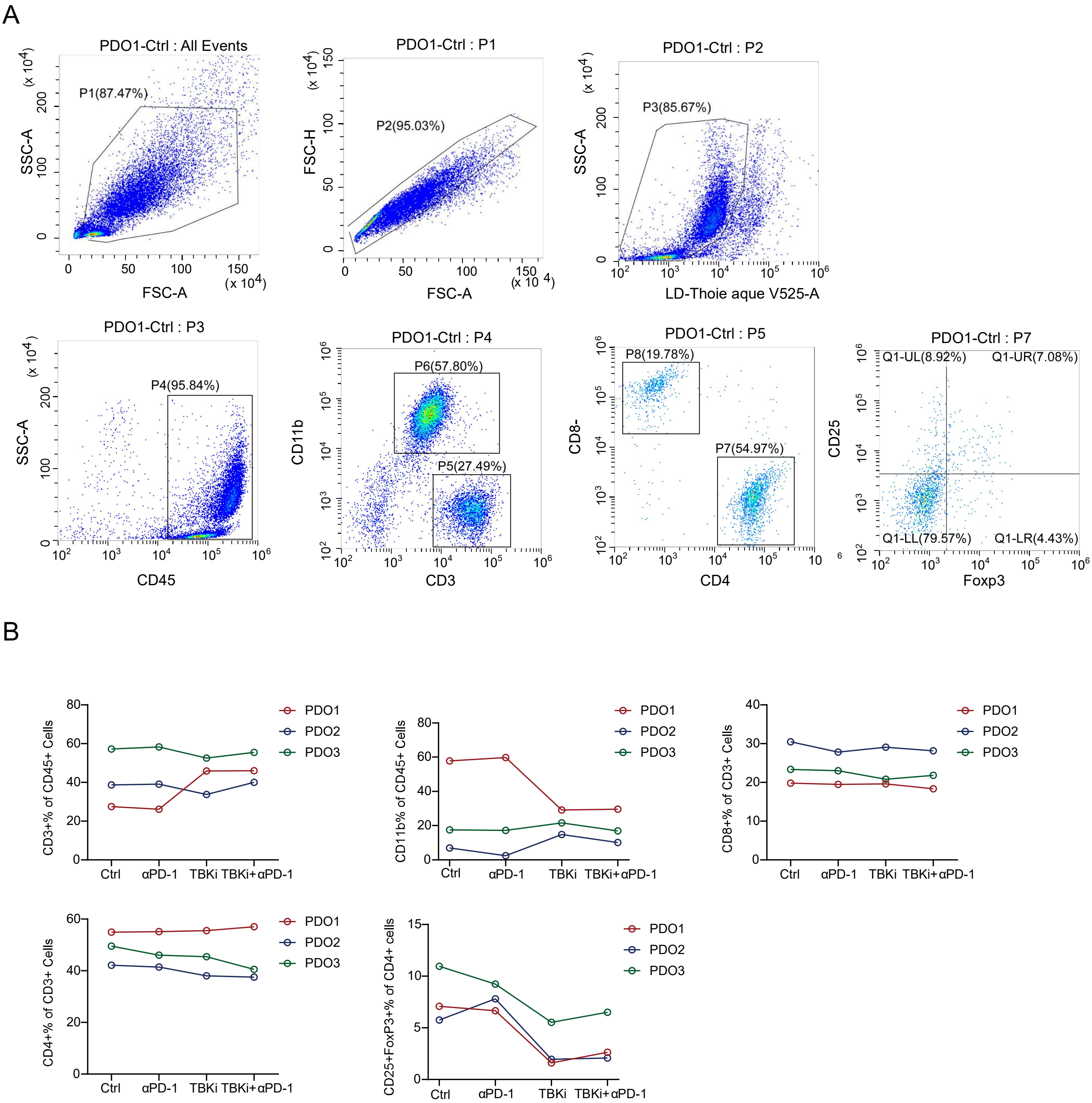

Appendix Figure S4. Flow cytometry profiling of immune subsets in effusion-derived organoid cultures under TBK inhibition and αPD-1 treatment.

(A) Representative gating strategy from PDO1 (Patient 1; EML4–ALK fusion) established from malignant pleural/ascitic effusion. Events were gated sequentially by FSC/SSC, singlets (FSC-A vs FSC-H), and live cells (LD-Thiol dye-negative), followed by identification of CD45+ leukocytes, CD3+ T cells, CD11b+ myeloid cells, CD4+ and CD8+ T-cell subsets, and Tregs defined as CD25+Foxp3+ cells within the CD4+ gate.
(B) Summary of immune subset frequencies across three patient effusion-derived organoid cultures (PDO1 = Patient 1, EML4–ALK fusion; PDO2 = Patient 2, EGFR exon 19 deletion; PDO3 = Patient 3, EGFR exon 19 deletion) under the indicated treatments (Ctrl, αPD-1, TBK inhibitor [TBKi], and TBKi + αPD-1). Plots show the percentage of CD3+ cells among CD45+ cells, CD11b+ cells among CD45+ cells, CD8+ cells among CD3+ cells, CD4+ cells among CD3+ cells, and CD25+Foxp3+ cells among CD4+ cells.
