# Supplementary material for: Oncogenic EGFR rewires STING-TBK1 signalosomes to license DNA damage tolerance in NSCLC
Source: EMBO J. 2026 Jul 10;45(16):5759–92. doi: 10.1038/s44318-026-00856-3 (PMC13482819; doi:10.1038/s44318-026-00856-3)
Supplement: Supplementary file 3 [file 44318_2026_856_MOESM6_ESM.pdf]

THE EMBO JOURNAL

# Oncogenic EGFR rewires STING-TBK1 signalosomes to license DNA damage tolerance in NSCLC

Qin Shen, Chen Mei, Yidan Chen, Qian Zhang, Qingzhe Wu, Xinyuan Yu, Fei Zhang, Shengduo Liu, Chen Chen, Cunqi Ye, Qi Zhang, Xin-Hua Feng, Hai Song, Tingbo Liang, Li Shen, Penghong Song, Bing Xia, and Pinglong Xu

*Corresponding authors: Pinglong Xu (xupl@zju.edu.cn) , Pinglong Xu (xupl@zju.edu.cn), Penghong Song (songpenghong@zju.edu.cn), Bing Xia (xiabing@hospital.westlake.edu.cn)*

## Review Timeline:

| | |
|---|---|
| Submission Date: | 19th Oct 25 |
| Editorial Decision: | 12th Dec 25 |
| Revision Received: | 15th Mar 26 |
| Editorial Decision: | 4th May 26 |
| Revision Received: | 26th Jun 26 |
| Accepted: | 1st Jul 26 |

*Editor: Daniel Klimmeck*

## Transaction Report:

(Note: With the exception of the correction of typographical or spelling errors that could be a source of ambiguity, letters and reports are not edited. Depending on transfer agreements, referee reports obtained elsewhere may or may not be included in this compilation. Referee reports are anonymous unless the Referee chooses to sign their reports.)

Dear Dr Xu,

Thank you again for the submission of your manuscript (EMBOJ-2025-122789) to The EMBO Journal, as well as providing us with a preliminary point-by-point response to the concerns raised by the referees. As mentioned, your study was assessed by three reviewers with expertise in cancer biology and immunity signaling, whose comments are enclosed below.

As you will see from their comments, the referees acknowledge the analysis and potential interest and value of your findings. However, they also express major concerns regarding the depth of insights provided on distinctive features enabling mutant EGFR to impact STING, TBK1, and downstream effectors, as well as the level of detail provided on related DNA repair aspects. They also raise substantial concerns regarding conclusive support for an endogenous relevance of the proposed signaling axis. Further, the reviewers raise a number of issues related to the presentation of the findings, clarity of the data presentation and plausibility of the claims from the data shown, that would need to be conclusively addressed to achieve the level of robustness and clarity needed for The EMBO Journal.

Given the overall interest stated and broader angle of your results, we are able to invite you to revise your manuscript experimentally to address the referees' comments, along the lines sketched in your outline. I need to stress though that we do require strong support from the referees on a revised version of the study in order to move on to publication of the work.

Please feel free to contact me if you have any questions or need further input on the referee comments.

We generally allow three months as standard revision time. As a matter of policy, competing manuscripts published during this period will not negatively impact on our assessment of the conceptual advance presented by your study. However, we request that you contact the editor as soon as possible upon publication of any related work, to discuss how to proceed. Should you foresee a problem in meeting this three-month deadline, please let us know in advance and we may be able to grant an extension.

When preparing your letter of response to the referees' comments, please bear in mind that this will form part of the Review Process File, and will therefore be available online to the community. For more details on our Transparent Editorial Process, please review our Editorial Policies: https://link.springer.com/partners/embo-press/editorial-policies

When submitting your revised manuscript, please carefully review the instructions below.

Please feel free to approach me any time should you have additional questions related to this.

Thank you for the opportunity to consider your work for publication.

I look forward to your revision.

Kind regards,

Daniel Klimmeck

Daniel Klimmeck, PhD
Senior Editor
The EMBO Journal

Instruction for the preparation of your revised manuscript:

Read our guidance for manuscript revisions and related editorial policies: https://link.springer.com/journal/44318/submission-guidelines#cms-Revised-submissions
1) a .docx formatted version of the manuscript text (including legends for main figures, EV figures and tables). Please make sure that the changes are highlighted to be clearly visible.

2) individual production quality figure files as .eps, .tif, .jpg (one file per figure).

3) a .docx formatted letter INCLUDING the reviewers' reports and your detailed point-by-point response to their comments. As part of the EMBO Press transparent editorial process, the point-by-point response is part of the Review Process File (RPF), which will be published alongside your paper.

4) a complete author checklist, which you can download from our author guidelines. Please insert information in the checklist that is also reflected in the manuscript. The completed author checklist will also be part of the RPF.

5) Please note that all corresponding authors are required to supply an ORCID ID for their name upon submission of a revised manuscript.

6) It is mandatory to include a 'Data Availability' section after the Materials and Methods. Before submitting your revision, primary datasets produced in this study need to be deposited in an appropriate public database, and the accession numbers and database listed under 'Data Availability'. Please remember to provide a reviewer password if the datasets are not yet public.
In case you have no data that requires deposition in a public database, please state so in this section. Note that the Data Availability Section is restricted to new primary data that are part of this study.
*** Note - All links should resolve to a page where the data can be accessed. ***

7) Our journal encourages inclusion of *data citations in the reference list* to directly cite datasets that were re-used and obtained from public databases. Data citations in the article text are distinct from normal bibliographical citations and should directly link to the database records from which the data can be accessed. In the main text, data citations are formatted as follows: "Data ref: Smith et al, 2001" or "Data ref: NCBI Sequence Read Archive PRJNA342805, 2017". In the Reference list, data citations must be labeled with "[DATASET]". A data reference must provide the database name, accession number/identifiers and a resolvable link to the landing page from which the data can be accessed at the end of the reference.

8) At EMBO Press we ask authors to provide source data for the main and EV figures. Our source data coordinator will contact you to discuss which figure panels we would need source data for and will also provide you with helpful tips on how to upload and organize the files.

Numerical data can be provided as individual .xls or .csv files (including a tab describing the data). For 'blots' or microscopy, uncropped images should be submitted (using a zip archive or a single pdf per main figure if multiple images need to be supplied for one panel.

9) We replaced Supplementary Information with Expanded View (EV) Figures and Tables that are collapsible/expandable online. A maximum of 5 EV Figures can be typeset. EV Figures should be cited as 'Figure EV1, Figure EV2" etc. in the text and their respective legends should be included in the main text after the legends of regular figures.

- For the figures that you do NOT wish to display as Expanded View figures, they should be bundled together with their legends in a single PDF file called *Appendix*, which should start with a short Table of Content. Appendix figures should be referred to in the main text as: "Appendix Figure S1, Appendix Figure S2" etc.

- Additional Tables/Datasets should be labelled and referred to as Table EV1, Dataset EV1, etc. Legends have to be provided in a separate tab in case of .xls files. Alternatively, the legend can be supplied as a separate text file (README) and zipped together with the Table/Dataset file.

10) When assembling figures, please refer to our figure preparation guideline in order to ensure proper formatting and readability in print as well as on screen:
https://media.springernature.com/original/springer-cms/rest/v1/content/27825798/data/v1

Please remember: Digital image enhancement is acceptable practice, as long as it accurately represents the original data and conforms to community standards. If a figure has been subjected to significant electronic manipulation, this must be noted in the figure legend or in the 'Materials and Methods' section. The editors reserve the right to request original versions of figures and the original images that were used to assemble the figure.

11) For data quantification: please specify the name of the statistical test used to generate error bars and P values, the number (n) of independent experiments (specify technical or biological replicates) underlying each data point and the test used to

calculate p-values in each figure legend. The figure legends should contain a basic description of n, P and the test applied. Graphs must include a description of the bars and the error bars (s.d., s.e.m.).

Please remember: Digital image enhancement is acceptable practice, as long as it accurately represents the original data and conforms to community standards. If a figure has been subjected to significant electronic manipulation, this must be noted in the figure legend or in the 'Materials and Methods' section. The editors reserve the right to request original versions of figures and the original images that were used to assemble the figure.

Further information is available in our Guide For Authors: https://link.springer.com/journal/44318/submission-guidelines#cms-Revised-submissions

Please remember: Digital image enhancement is acceptable practice, as long as it accurately represents the original data and conforms to community standards. If a figure has been subjected to significant electronic manipulation, this must be noted in the figure legend or in the 'Materials and Methods' section. The editors reserve the right to request original versions of figures and the original images that were used to assemble the figure.

We realize that it is difficult to revise to a specific deadline. In the interest of protecting the conceptual advance provided by the work, we recommend a revision within 3 months (12th Mar 2026). Please discuss the revision progress ahead of this time with the editor if you require more time to complete the revisions. Use the link below to submit your revision:

https://emboj.msubmit.net/cgi-bin/main.plex

EMBOJ-2025-122789; referee reports:

Referee #1:

The manuscript by Shen et al. addresses molecular mechanisms involved in mutant EGFR (mEGFR)-driven NSCLC. Specifically, they investigated the interplay between mEGFR and innate immune signaling pathways, focusing on the STING-TBK1-IRF3 pathway. Their goal was to identify therapeutic targets for overcoming resistance to TKI and chemotherapy in NSCLC.
They unexpectedly found that EGFR hotspot mutations hijack the STING-TBK1 pathway, which instead of a tumor-suppressive function, acts as a pro-survival driver of chemoresistance. These mEGFR also reinforce the DNA repair machinery. Importantly, targeting this aberrant mEGFR-STING-TBK1 axis restores chemosensitivity, which uncovers a novel framework for overcoming TKI and chemotherapy resistance in mEGFR-driven NSCLC.

The manuscript is well-written, and the experimental approach is sound and straightforward. Outstanding quality of the data, with orthogonal approaches used to test the different hypotheses and molecular mechanisms. A combination of in vitro and in vivo experiments provide strength to their conclusions, which are justified by the results. Overall, the data presented support the idea that TBK1 plays a critical role in oncogenic signaling by controlling mEGFR-driven NSCLC, challenging the conventional view of STING-TBK1-mediated antitumor immunity (tumor suppressor function), Thus, exploiting this STING-TBK1 vulnerability of mEGFR-driven NSCLC can offer a promising strategy against chemoresistance to eliminate tumor burden. Overall, major strengths are found summarized below, and only minor caveats.

Strengths:
• Demonstrate that EGFR hotspot mutations, but not wtEGFR, is indispensable for cGAS-STING activation in NSCLC cells. Strong data demonstrate that EGFR mutants that are hyperactivated in NSCLC (constitutive activation of EGFR tyrosine kinase) amplify STING signaling, but not MAVS. Activation of STING-TBK1-IRF3 signaling by diABZI (STING agonist) or cGAMP did not happen in the presence of wtEGFR, only in the presence of mEGFR, and inhibiting or depleting mEGFR activity abrogated diABZI- and cGAMP-induced STING signaling. Moreover, trafficking of STING and TBK1 to the Golgi or upregulation of inflammatory cytokines was hindered by inhibiting mEGFR activity.
• Strong evidence is presented for activating mEGFR mutants integrating physically into STING signalosomes. Elegant studies using inducible expression of mEGFR or wtEGFR and diABZI stimulation of STING show the presence of mEGFR/STING/TBK1 clusters/puncta, indicating that mEGFR participates in STING signalosomes. The presence of mEGFR at STING signalosome is abrogated by mEGFR inhibtion. IF also shows differences in localization: mEGFR at Golgi and endosomes, and wtEGFR mainly anchored to PM. Co-IPs validated the interaction of endogenous STING and TBK1 with mEGFR in a kinase-dependent manner. Importantly, mEGFR enhanced the association of TBK1 with STING and IRF3, suggesting a role in signalosome assembly. Using mEGFR protein truncation mutants found that mEGFR, but not wtEGFR, is an integral component of classical STING-TBK1-IRF3 signalosomes.
• Reveal that tyrosine phosphorylation of endogenous STING and TBK1 by mEGFR (abrogated by mEGFR kinase inhibitors)

solidifies STING clustering. By MS analysis and individual Tyr point mutations of TBK1, they identified Y577/Y677 as key sites for mEGFR-mediated TBK1 phosphorylation. Importantly, residue Y577 is a key interaction necessary for the integration of TBK1 into the STING signalosomes. For STING, Y245 and Y314 are the main sites of mEGFR-mediated phosphorylation. Phosphomimetic mutants of STING Y245E and Y314E bind strongly to mEGFR and TBK1, suggesting a tyrosine phosphorylation-based recruitment of mEGFR and TBK1 into STING signalosomes. These strong data indicate that mEGFR phosphorylates STING and TBK1, potentiating STING signalosome assembly.
• Strong evidence that TBK1 phosphorylation at Y577/Y677 stabilizes activated TBK1. Demonstrated that mEGFR-mediated phosphorylation of TBK1 at Y577/Y677 protected it from K48-Ub modification and proteasomal degradation, thus sustaining STING-TBK1 signaling.
• Propose that STING and TBK1 are indispensable for mEGFR-driven NSCLC survival. They state that depletion of either TBK1 or STING substantially inhibited H1975 growth to a level comparable to mEGFR depletion. In addition, TBK1 depletion potently impeded colony formation of mEGFR-driven NSCLC cells, while barely impacting colony formation in H1299 cells harboring wild-type EGFR, suggesting a unique dependency and vulnerability of mEGFR-driven cells on TBK1. They also used an in vivo immune-independent model to validate the proposed role of STING signalosomes in mEGFR-driven NSCLC viability. They demonstrate that depletion of TBK1 in tumors growing subcutaneously in mice significantly attenuated tumor growth and tumor weight.
• RNAseq analysis of NSCLC cells with mEGFR and upon depletion of TBK1 show upregulation of inflammatory signaling pathways and downregulation of DNA repair and replication pathways. These data suggest that TBK1 maintains genome stability in mEGFR-expressing NSCLC. Monitoring response of these cells to cisplatin found that mEGFR-expressing cells are more susceptible to DNA damage by cisplatin when TBK1 is depleted. Cisplatin leads to robust activation of cGAS-STING-TBK1-IRF3 signaling, as well as STING-dependent expression of ISGs. Co-IP shows that cisplatin facilitates the formation of EGFR-STING-TBK1 complex. Importantly, treatment with cisplatin and TBK1 inhibitor (or TBK1 depletion) substantially and synergistically attenuated colony formation. Similar results with STING depletion. All these data show that targeting STING and TBK1 signaling in mEGFR-driven NSCLC impairs DNA damage repair and sensitizes tumors to chemotherapy.
• Used various ex-vivo and in-vivo models of NSCLC tumorigenesis to test their findings in NSCLC cells in vitro. Combining cisplatin and TBK1 inhibitors eradicated TKI-resistant NSCLC in patient-derived organoids and mEGFR-driven spontaneous NSCLC tumors. Moreover, mEGFR-driven lung carcinogenesis in an immunocompetent mouse model was used to monitor the response to a month-long combinational therapy. Cisplatin and TBK1 inhibitor attenuated tumor progression more than each monotherapy.
Caveats:
• Given that they are studying the cGAS-STING-TBK1-IFN pathway, did they find increased expression of IFNs? Other inflammatory cytokines and ISGs are shown, but not IFNs. This should be discussed.
• Is there enough evidence that mEGFR directly phosphorylates STING and TBK1 on tyrosyines? Is it possible that another kinase acts as an intermediary?
• In Figure 5, the indispensable role of TBK1 on mEGFR-driven NSCLC survival in vitro and tumor growth in vivo is clearly demonstrated, but not so much the role of STING. The impact of STING depletion is not evaluated. Only in Figures 5A and 5C they monitor proliferation and apoptosis upon depletion of STING, and it does not seem that STING depletion has a major effect on cell survival, or at least is much less pronounced than TBK1 depletion.

Referee #2:

In the manuscript, entitled "Oncogenic EGFR rewires STING-TBK1 immune machinery by tyrosine phosphorylation to license DNA damage tolerance," Qui Shen and collaborators present a potentially important observation relevant to cancer biology. The authors report a previously unknown mechanism by which mutant EGFR (mEGFR), a key oncogenic driver in non-small cell lung cancer (NSCLC), hijacks the innate immune cGAS-STING pathway to promote tumor survival and drug resistance.

The core finding is that mEGFR (e.g., L858R/T790M), but not wild-type EGFR, physically integrates into the STING signalosome. It then directly phosphorylates STING and TBK1 on specific tyrosine residues. These tyrosine phosphorylation events serve two functions:

• Stabilizing the signalosome by enhancing the assembly and stability of the STING-TBK1 complex.
• Stabilizing TBK1 by means of inhibiting K48-linked ubiquitination of TBK1, thereby preventing its proteasomal degradation and leading to sustained kinase activation.

Critical comments

1) Line 107: In the final paragraph of the Introduction, the authors frame their study by stating that prior research has largely characterized cGAS-STING signaling as a tumor-suppressive pathway, thereby highlighting their own findings as unexpected. However, this framing does not accurately reflect the current state of the field. Several studies over the past decade, for example, a comprehensive and mechanistically detailed report by Lv et al., in Cell Chemical Biology (2023), have already

demonstrated that cancer cell-autonomous activation of cGAS-STING can promote tumor progression and therapeutic resistance. Therefore, the authors' assertion that cGAS-STING has been "traditionally" considered solely antitumor is an oversimplification, and invoking this narrative to underscore novelty is misleading.

2) In sFigure 1, the authors employed HEK293T cells to evaluate the effects of EGFR hotspot mutations on STING or MAVS-induced IRF3 transactivation. However, the manuscript does not provide any rationale for choosing this cell line, even though HEK293T cells are widely known to lack functional endogenous cGAS-STING signaling and possess attenuated MAVS pathway activity. Without this clarification, readers may be left uncertain about why this particular cell line was chosen and what the specific experimental rationale is.

3) In Figure 1B, the authors present immunofluorescence images for STING, TBK1, and the corresponding MERGE panels. However, the DAPI (nuclear) channel is not shown anywhere in the figure, despite being required to generate the MERGE images.

4) The authors begin by presenting the results for Figure 1, which is intended to establish the central premise of the manuscript-namely, that EGFR hotspot mutations enhance cGAS-STING-TBK1-IRF3 signaling. However, the immunoblots in Figure 1 uniformly lack any assessment of STING or phospho-STING, which are the core components of this pathway. As a result, although the authors show in Figure 1 that IRF3 transactivation is stimulated by STING rather than MAVS, the current experiments do not distinguish whether EGFR mutations enhance STING-dependent signaling or instead amplify STING-independent TBK1/IRF3 activation. This represents a fundamental mechanistic ambiguity that undermines the central claim of the manuscript. The authors need to include direct measurements of STING and phospho-STING in Figure 1 (and related figures), as well as clarify whether the observed effects are genuinely STING-dependent.

5) In Figure 2, the authors conclude that mEGFR "integrates into STING-TBK1 signalosomes" based largely on 2D confocal images showing apparent overlap between STING/TBK1 puncta and EGFR. However, given the crowded perinuclear environment and the proximity of ER, Golgi, and endosomal membranes, 2D projections can be misleading and do not definitively demonstrate that these proteins occupy the same structures in three dimensions. A 3D reconstruction would confirm this result.

6) The manuscript shows how mEGFR interacts with STING/TBK1, but the fundamental reason why mEGFR, and not wtEGFR, gains this ability is insufficiently explored. The authors suggest increased kinase activity and altered subcellular localization (more endosomal) as potential mechanisms, but a deeper biochemical or biophysical explanation is still lacking.

7) The DNA repair link remains indirect: While the transcriptomic data (RNA-seq and GSEA) showing downregulation of DNA repair pathways upon TBK1 depletion is compelling, direct mechanistic links between TBK1 and the DNA repair machinery are not fully elucidated. The authors mention potential players, such as PARP, ATM and ISG15, but do not investigate them. How exactly sustained TBK1 activation transcriptionally or post-translationally boosts DNA repair remains uncharacterized.

8) The study rightly focuses on the cancer cell-intrinsic, non-canonical role of STING-TBK1. However, the title and introduction frame it as rewiring "immune machinery." A discussion on how this tumor-cell-intrinsic hijacking of STING might influence the broader tumor immune microenvironment (e.g., affecting T-cell infiltration or function) would strengthen the study. Exploring the consequences in the tumor microenvironment (TME), using syngeneic or genetically engineered immunocompetent models would dissect how tumor-intrinsic mEGFR-STING-TBK1 signaling shapes the immune landscape.

9) Does the uncovered signaling axis lead to the secretion of specific cytokines and chemokines? Similarly, does it alter the ratio of CD8+ T cells to Tregs or myeloid-derived suppressor cells (MDSCs)?

10) The authors need to address the following question: does TBK1 inhibition, by killing tumor cells, indirectly boost anti-tumor immunity or it induces a direct immunosuppressive effect?

11) Specificity to mEGFR: While the data support the role in mEGFR-driven NSCLC, it would be important to know if other oncogenic kinases (e.g., ALK and ROS1 fusions) can similarly hijack the STING pathway, or if this is a unique feature of mEGFR.

Referee #3:

In their article 'Oncogenic EGFR rewires STING-TBK1 immune machinery by tyrosine phosphorylation to license DNA damage tolerance', Shen et al. describe a new intersection between STING-dependent signalling and mutant EGFR. They provide evidence that mEGFR associates with both STING and TBK1, and that it can support TBK1-dependent signalling. Some of the data also suggest that mEGFR is required for STING/TBK1-dependent signalling, and that it may rewire the transcription

outputs. Ultimately, they suggest that in mEGFR cell lines, TBK1 becomes important for cell proliferation, and that loss of TBK1, or its inhibition can synergise with cisplatin treatment, in cell lines as well as in murine models.
Whilst the data are intriguing and abundant, several key points are not clear. The mechanistic basis is not described in sufficient detail, and some discrepancies in the data exist that would need reconciliation. The manuscript also relies a bit too much on the (over?)expression of exogenous proteins, rather than analysis of endogenous proteins.

Major points
1. It is unclear how mEGFR intersects with STING/TBK1. In some experiments it looks like it supports signalling following STING agonist treatment, but in other experiments, it seems like there is strong baseline signalling in the absence of other stimuli. For example, the microscopy (Fig. 2A-C) suggests that diABZI addition is required for the co-localisation between mEGFR and STING, but then in the IPs (Fig. 2D for example), no stimulation is required. In Fig. 1G, diABZI addition is required to see an impact of mEGFR on TBK1ph, but in Fig. 3B,E,H, this is not the case. In Fig. 4C, no stimulation is required for mEGFR to regulate TBK1 stability. In the colony formation/viability experiments, no external cGAS or STING stimuli are added. Furthermore, it seems like in mEGFR cell lines, mEGFR is required for STING-dependent signalling (for example, Fig. 2B, EGFR inhibition prevents STING relocalisation to the Golgi), raising the question, how STING can signal in cells that do not express mEGFR. So overall, it isn't clear to me what the actual message is. Is it that mEGFR promotes STING signalling? Or that it somehow is required for STING signalling? Finally, the authors usually only look at one time point, but all that might occur in mEGFR cells is that kinetics are modified.
2. The molecular mechanism by which the authors propose that mEGFR regulates STING/TBK1 (direct interaction) is not proven by the experiments, which rely on pulldowns with overexpressed proteins. These types of experiments to do not report on direct molecular interactions. To validate their model, recombinant proteins would be needed.
3. In many experiments, the readouts are not benchmarked against bona fide cGAS-STING stimuli. For example, the initial HEK293T experiments (Fig. S1AB) look at STING expression in HEK293T cells, which leads to weak autoactivation, which is stimulated by mEGFR. However, the output is not benchmarked against what could be achieved by co-expressing cGAS with STING, whilst also providing a DNA stimulus, or -alternatively, what could be achieved by addition of diABZI. This kind of control is important, as it allows the true level of impact of mEGFR to be assessed.
4. The microscopy experiments (Fig. 1B and 2A-C and Fig. S2) need to be quantified. The authors also make too sweeping statements about the localisation patterns. For example, in Fig. 1B, the differences of with and without WZ4002 are extremely subtle, especially for TBK1, but are treated as highly different in the text. Also, with regards to the relocalisation of mEGFR following diABZI addition, does this depend on STING?
5. Quantifications should also be added for other experiments (basically all, but just to name a few, Fig. 4A,C,F).
6. There are some inconsistencies in the data, and some data that do not seem to support the authors' conclusions. For example, (a) in Fig. 1F, I do not see any obvious difference between cells with and without mEGFR as far as pTBK1 and pIRF3 are concerned, especially once differential loading is accounted for. (b) I am not convinced that the data as shown support the idea that TBK1 levels are regulated by mEGFR (see for example Fig. 4A). (c), the impact of TBK1 mutations on expression levels seen in Fig. 4J is not recapitulated in the HEK system (Fig. 3C and 3H and S3A) and also not in the H1975 system (Fig. 4A). (d) Fig. 5I and Fig. 5J seem to show different effects for Tet-shCtrl Dox+.
7. With regards to the viability impact of TBK1 loss on mEGFR cell lines, DepMap does not seem to show these effects. Could the authors do a wider analysis of DepMap data, cross-referencing with EGFR status to gain broader support of their hypothesis? Similarly, could TCGA data be analysed? The prediction would be that there should be strong selection to maintain cGAS/STING/TBK1 in mEGFR cancers.
8. None of the IP experiments contain negative controls (proteins that shouldn't be pulled down such as actin or tubulin), which can be used to assess how clean the pulldowns are. The IPs are also a bit confusing for other reasons. STING is degraded upon activation, and it is therefore difficult to see how a stable complex between mEGFR, STING and TBK1 could persist for prolonged periods of time. Furthermore, Fig. 2F suggests interactions with both STING and TBK1, but then in Fig. 2G interactions with TBK1 are observed in the absence of STING. Is the idea that mEGFR interacts with TBK1, and this bridges to STING? But again, such claims would have to be backed up by more detailed biochemistry.
9. In general, the manuscript relies too much on overexpression of transgenes, which is utilised in almost every experiment. Obviously for introducing mEGFR in EGFR wt cells, this is acceptable, but I don't quite understand why impact on wt TBK1 and STING usually is not assayed with the endogenous proteins but with tagged exogenously expressed ones (for example, Fig. S4).
10. In most shRNA experiments, rescue experiments are not performed.
11. The mRNAseq analysis is rather confusing. Why are inflammatory genes up when TBK1 is lost? This is the opposite of what one would expect.
12. The dose-response curves (Fig. S6H-K and Fig. 7B) do not have statistical tests.

Minor points
1. In Fig. 1D, the use of poly(dA:dT) as a cGAS stimulant is odd, as this was indicated to trigger Rig-I dependent signalling (PMID 19609254). The authors should use plasmid DNA or herring testes DNA instead.
2. The text describing Fig. 1H and S1E says that interferon levels were analysed but these are not shown in the figures.
3. Fig. 6IJ and and 7A: Please show the full y axis!
4. Please keep colours consistent between Fig. 2A and 2B/C.
5. Lines 205-206: 'mEGFR coexpression revealed a notable reduction in pY100 signals in TBK1 Y577F, Y677F, or Y577F/Y677F mutants' is poorly expressed and ambiguous in its meaning.

6. I cannot find the data for Lines 301-303 ('Coimmunoprecipitation assays indicated that cisplatin facilitated the formation of EGFR-STING-TBK1 tripartite complexes, thus potentially linking mEGFR to STING signalosome activation and drug resistance.')
7. Please avoid words like "eradicate" and "impressive" when describing effects.

**Response to the reviewers (EMBOJ-2025-122789):**

We sincerely thank the editors and reviewers for their outstanding efforts, thoughtful evaluation, and constructive feedback on our manuscript. Guided by the reviewers' suggestions, we conducted a range of experiments during revision that addressed all concerns and expanded on the innovative idea of oncogenic EGFR rewires STING-TBK1 machinery by tyrosine phosphorylation to license DNA damage tolerance .

The major additions and clarifications are summarized below: (1) we strengthened the central mechanistic claim by adding new biochemical evidence for mEGFR-associated phosphorylation of STING and TBK1, including an in vitro kinase reaction, while revising the text to adopt a more cautious interpretation of these findings; (2) expanded the DDR studies, including observed TBK1-regulated DDR components, phenotypes, and rescue experiments, to strengthen the link between TBK1 and cisplatin-associated DDR and HR phenotypes, and we revised the interpretation of the transcriptomic and GSEA data accordingly; (3) reinforced the evidence that mEGFR associates with STING and TBK1 assemblies in cells by adding 3D reconstruction, orthogonal views, and further proximity and colocalization quantification; (4) clarified the dynamics of this coupling by performing finer time-course analyses, allowing us to better distinguish basal association from stimulus-enhanced activation and to define effects on pathway amplitude versus persistence; (5) extended the immune-context analyses and clarified the scope of the manuscript by further characterizing the immunocompetent mEGFR-driven model, analyzing immune-exclusion features, and adding exploratory immune profiling in PDO-based settings; (6) extended the analysis beyond mEGFR by performing additional exploratory studies with other oncogenic RTKs, including EML4-ALK and CD74-ROS1, to better define the scope of this phenomenon; (7) we refined the functional hierarchy within the pathway through additional analyses of the relative contributions of STING and TBK1 to tumor-cell phenotypes; (8) added further pathway-specificity and mechanistic support, including direct STING and pSTING analyses, alternative DNA-stimulation controls, and additional association and dependency controls for the EGFR-STING-TBK1 complex; (9) included exploratory TCGA-based clinical-context analyses; (10) improved statistical rigor and data transparency by expanding figure quantification, source-data support, and dose-response analyses across the revised figures.

These substantial sets of new or updated data are now presented in **Figures 1B-E, 1G, 1H, 2A-D, 3F, 4A, 4K-L, 4H, 5D, 6D-F, 6H-J, Expanded View Figures 1E, 1G, 1I, 2A, 2C-H, 3B-E, 4D, and Appendix Figures S1A-B, S2A, S3A-C, S4A-B, S5A-B, S6A, S7A-C, S8A-B** in the revised manuscript. Meanwhile, we have restructured the data sets and carefully revised the text, making necessary corrections and adding more precise descriptions. We believe we have addressed all concerns/questions raised by the reviewers, both experimentally and in the literature, and that the revised manuscript has been substantially strengthened.

Below, we provide a detailed point-by-point response to each comment:

**Reviewer #1:**

The manuscript by Shen et al. addresses molecular mechanisms involved in mutant EGFR (mEGFR)-driven NSCLC. Specifically, they investigated the interplay between mEGFR and innate immune signaling pathways, focusing on the STING-TBK1-IRF3 pathway. Their goal was to identify therapeutic targets for overcoming resistance to TKI and chemotherapy in NSCLC.

They unexpectedly found that EGFR hotspot mutations hijack the STING-TBK1 pathway, which, instead of a tumor-suppressive function, acts as a prosurvival driver of chemoresistance. These mEGFR also reinforce the DNA repair machinery. Importantly, targeting this aberrant mEGFR-STING-TBK1 axis restores chemosensitivity, which uncovers a novel framework for overcoming TKI and chemotherapy resistance in mEGFR-driven NSCLC.

The manuscript is well-written, and the experimental approach is sound and straightforward. Outstanding quality of the data, with orthogonal approaches used to test the different hypotheses and molecular mechanisms. A combination of in vitro and in vivo experiments provides strength to their conclusions, which are justified by the results. Overall, the data presented support the idea that TBK1 plays a critical role in oncogenic signaling by controlling mEGFR-driven NSCLC, challenging the conventional view of STING-TBK1-mediated antitumor immunity (tumor suppressor function). Thus, exploiting this STING-TBK1 vulnerability of mEGFR-driven NSCLC can offer a promising strategy against chemoresistance to eliminate tumor burden. Overall, major strengths are found summarized below, and only minor caveats.

Strengths:

• Demonstrate that EGFR hotspot mutations, but not wtEGFR, is indispensable for cGAS-STING activation in NSCLC cells. Strong data demonstrate that EGFR mutants that are hyperactivated in NSCLC (constitutive activation of EGFR tyrosine kinase) amplify STING signaling, but not MAVS. Activation of STING-TBK1-IRF3 signaling by diABZl (STING agonist) or cGAMP did not happen in the presence of wtEGFR, only in the presence of mEGFR, and inhibiting or depleting mEGFR activity abrogated diABZl- and cGAMP-induced STING signaling. Moreover, trafficking of STING and TBK1 to the Golgi or upregulation of inflammatory cytokines was hindered by inhibiting mEGFR activity.

• Strong evidence is presented for activating mEGFR mutants integrating physically into STING signalosomes. Elegant studies using inducible expression of mEGFR or wtEGFR and diABZl stimulation of STING show the presence of mEGFR/STING/TBK1 clusters/puncta, indicating that mEGFR participates in STING signalosomes. The presence of mEGFR at STING signalosome is abrogated by mEGFR inhibtion. IF also

shows differences in localization: mEGFR at Golgi and endosomes, and wtEGFR mainly anchored to PM. Co-IPs validated the interaction of endogenous STING and TBK1 with mEGFR in a kinase-dependent manner. Importantly, mEGFR enhanced the association of TBK1 with STING and IRF3, suggesting a role in signalosome assembly. Using mEGFR protein truncation mutants found that mEGFR, but not wtEGFR, is an integral component of classical STING-TBK1-IRF3 signalosomes.

• Reveal that tyrosine phosphorylation of endogenous STING and TBK1 by mEGFR (abrogated by mEGFR kinase inhibitors) solidifies STING clustering. By MS analysis and individual Tyr point mutations of TBK1, they identified Y577/Y677 as key sites for mEGFR-mediated TBK1 phosphorylation. Importantly, residue Y577 is a key interaction necessary for the integration of TBK1 into the STING signalosomes. For STING, Y245 and Y314 are the main sites of mEGFR-mediated phosphorylation. Phosphomimetic mutants of STING Y245E and Y314E bind strongly to mEGFR and TBK1, suggesting a tyrosine phosphorylation-based recruitment of mEGFR and TBK1 into STING signalosomes. These strong data indicate that mEGFR phosphorylates STING and TBK1, potentiating STING signalosome assembly.

• Strong evidence that TBK1 phosphorylation at Y577/Y677 stabilizes activated TBK1. Demonstrated that mEGFR-mediated phosphorylation of TBK1 at Y577/Y677 protected it from K48-Ub modification and proteasomal degradation, thus sustaining STING-TBK1 signaling.

• Propose that STING and TBK1 are indispensable for mEGFR-driven NSCLC survival. They state that depletion of either TBK1 or STING substantially inhibited H1975 growth to a level comparable to mEGFR depletion. In addition, TBK1 depletion potently impeded colony formation of mEGFR-driven NSCLC cells, while barely impacting colony formation in H1299 cells harboring wild-type EGFR, suggesting a unique dependency and vulnerability of mEGFR-driven cells on TBK1. They also used an in vivo immune-independent model to validate the proposed role of STING signalosomes in mEGFR-driven NSCLC viability. They demonstrate that depletion of TBK1 in tumors growing subcutaneously in mice significantly attenuated tumor growth and tumor weight.

• RNAseq analysis of NSCLC cells with mEGFR and upon depletion of TBK1 shows upregulation of inflammatory signaling pathways and downregulation of DNA repair and replication pathways. These data suggest that TBK1 maintains genome stability in mEGFR-expressing NSCLC. Monitoring the response of these cells to cisplatin found that mEGFR-expressing cells are more susceptible to DNA damage by cisplatin when TBK1 is depleted. Cisplatin leads to robust activation of cGAS-STING-TBK1-IRF3 signaling, as well as STING-dependent expression of ISGs. Co-IP shows that cisplatin facilitates the formation of the EGFR-STING-TBK1 complex. Importantly, treatment

with cisplatin and TBK1 inhibitor (or TBK1 depletion) substantially and synergistically attenuated colony formation. Similar results with STING depletion. All these data show that targeting STING and TBK1 signaling in mEGFR-driven NSCLC impairs DNA damage repair and sensitizes tumors to chemotherapy.

- Used various ex-vivo and in-vivo models of NSCLC tumorigenesis to test their findings in NSCLC cells in vitro. Combining cisplatin and TBK1 inhibitors eradicated TKI-resistant NSCLC in patient-derived organoids and mEGFR-driven spontaneous NSCLC tumors. Moreover, mEGFR-driven lung carcinogenesis in an immunocompetent mouse model was used to monitor the response to a month-long combinational therapy. Cisplatin and TBK1 inhibitor attenuated tumor progression more than each monotherapy.

We very much appreciate the reviewer for your positive and constructive evaluation and for highlighting the novelty and conceptual advances of our manuscript. We have further addressed the caveats raised below by complementing experiments, incorporating supporting data into the Expanded View, clarifying the text, and adding discussion where appropriate.

Caveats:

**Comment 1:** Given that they are studying the cGAS-STING-TBK1-IFN pathway, did they find increased expression of IFNs? Other inflammatory cytokines and ISGs are shown, but not IFNs. This should be discussed.

We thank the reviewer for this important comment. We agree that IFN induction should be addressed explicitly. In EGFR-mutant NSCLC cells, activation of the pathway induces interferon output that is cell-context dependent. In H1975 cells, IFNβ mRNA is robustly induced upon diABZI stimulation and this induction is strongly suppressed by the mEGFR inhibitor WZ4002 **(Fig. 1J)**. In HCC827 cells, IFNβ was not significantly upregulated under the same conditions, whereas multiple interferon-stimulated genes (ISGs), including ISG15 and CXCL10, were readily induced **(Fig. EV1J)**. We therefore interpret these data as supporting enhanced STING-pathway output in EGFR-mutant NSCLC cells, while avoiding overstatement regarding a uniform interferon response across models.

We agree that this finding requires discussion, because type I interferons and ISGs are often associated with antitumor immunity. In our model, however, the net effect of this pathway appears not to be tumor-suppressive. Rather, our data suggest that, in mutant EGFR-driven NSCLC, tumor-intrinsic STING-TBK1 signaling is rewired, with its protumor and prosurvival functions predominating over any potential antitumor immune effects. Consistent with this interpretation, inhibition of mutant EGFR or attenuation of STING-TBK1 signaling significantly reduced tumor phenotypes in vitro and in vivo. We have therefore revised the Results section and expanded the discussion to emphasize the context-dependent nature of interferons.

**Comment 2:** Is there enough evidence that mEGFR directly phosphorylates STING and TBK1 on tyrosyines? Is it possible that another kinase acts as an intermediary?

We agree that the original version relied mainly on cell-based phosphorylation analyses and therefore did not fully exclude the possibility that an intermediary kinase contributes to the tyrosine phosphorylation of STING and TBK1. To strengthen this point, we have now added an *in vitro* kinase assay using immunopurified Flag-tagged mEGFR, STING, and TBK1 from HEK293T cells. In this assay, pY100 signal was detected on both STING and TBK1 only in the presence of mEGFR **(Fig. 3F)**. While this assay does not constitute a fully reconstituted system with purified recombinant components and therefore does not formally exclude the contribution of a tightly associated kinase, it does provide biochemical support that mEGFR-associated kinase activity can directly phosphorylate STING and TBK1 *in vitro*. These results are consistent with our endogenous pY100 immunoprecipitation data and mutagenesis analyses, and together strengthen the conclusion that mEGFR directly promotes tyrosine phosphorylation of STING and TBK1.

**Comment 3:** In Figure 5, the indispensable role of TBK1 on mEGFR-driven NSCLC survival in vitro and tumor growth in vivo is clearly demonstrated, but not so much the role of STING. The impact of STING depletion is not evaluated. Only in Figures 5A and 5C they monitor proliferation and apoptosis upon depletion of STING, and it does not seem that STING depletion has a major effect on cell survival, or at least is much less pronounced than TBK1 depletion.

We thank the reviewer for this important point and agree that the role of STING, relative to TBK1, should be more clearly defined. Our data show that TBK1 depletion consistently has a stronger effect on tumor cell survival, as expected given its role as a central signaling node. Our interaction data also support this hierarchical relationship. EGFR immunoprecipitation revealed that TBK1 depletion reduced EGFR-STING association, whereas EGFR-TBK1 association remained detectable upon STING depletion **(Appendix Fig. S8B)**. Moreover, besides the findings that STING contributes to mEGFR-driven phenotypes **(Fig. 5A, 5C)**, we have added new Expanded View data showing that STING depletion reduces clonogenic growth in H1975 cells **(Fig. EV3C)**, while having a much smaller effect in wtEGFR H1299 cells **(Fig. EV3D)**, consistent with a mEGFR context-dependent role for STING. We have therefore revised the Results section to separate the effects of STING and TBK1 perturbation and to present STING as a contributing component of this pathway in our model.

**Reviewer #2:**

In the manuscript, entitled "Oncogenic EGFR rewires STING-TBK1 immune machinery

by tyrosine phosphorylation to license DNA damage tolerance," Qui Shen and collaborators present a potentially important observation relevant to cancer biology. The authors report a previously unknown mechanism by which mutant EGFR (mEGFR), a key oncogenic driver in non-small cell lung cancer (NSCLC), hijacks the innate immune cGAS-STING pathway to promote tumor survival and drug resistance.

The core finding is that mEGFR (e.g., L858R/T790M), but not wild-type EGFR, physically integrates into the STING signalosome. It then directly phosphorylates STING and TBK1 on specific tyrosine residues. These tyrosine phosphorylation events serve two functions:

- Stabilizing the signalosome by enhancing the assembly and stability of the STING-TBK1 complex.
- Stabilizing TBK1 by means of inhibiting K48-linked ubiquitination of TBK1, thereby preventing its proteasomal degradation and leading to sustained kinase activation.

Critical comments：

We very much appreciate the reviewer's positive and constructive evaluation of our study. We have further addressed the concerns raised below through an array of additional experiments and by incorporating supporting data to solidify our findings.

**Comment 1:** Line 107: In the final paragraph of the Introduction, the authors frame their study by stating that prior research has largely characterized cGAS-STING signaling as a tumor-suppressive pathway, thereby highlighting their own findings as unexpected. However, this framing does not accurately reflect the current state of the field. Several studies over the past decade, for example, a comprehensive and mechanistically detailed report by Lv et al., in Cell Chemical Biology (2023), have already demonstrated that cancer cell-autonomous activation of cGAS-STING can promote tumor progression and therapeutic resistance. Therefore, the authors' assertion that cGAS-STING has been "traditionally" considered solely antitumor is an oversimplification, and invoking this narrative to underscore novelty is misleading.

We agree that the field has advanced substantially and that cGAS-STING is now appreciated as a context-dependent pathway with both tumor-suppressive and tumor-promoting functions. In particular, several studies have shown that cancer cell-autonomous cGAS-STING signaling can support tumor progression, inflammatory adaptation, and therapeutic resistance. We have therefore revised the Introduction to remove the oversimplified framing that cGAS-STING has been viewed as exclusively antitumor, and we now specifically cite prior studies demonstrating pro-tumorigenic, tumor-intrinsic cGAS-STING signaling (Lv *et al*, 2023; Hong *et al*, 2022).

We also clarified the conceptual distinction of our study. Our work does not, in general, claim that tumor-intrinsic STING signaling can be pro-tumorigenic. Rather, we identify a specific oncogene-driven rewiring mechanism in which a hotspot mutation in a key

cancer driver, mutant EGFR, engages and exploits the STING-TBK1 machinery to support NSCLC cell survival. Mechanistically, we show that mEGFR physically associates with STING and TBK1, promotes their tyrosine phosphorylation, stabilizes activated TBK1 by suppressing K48-linked ubiquitination, and functionally couples this signaling axis to DNA damage repair and cisplatin tolerance in EGFR-mutant NSCLC. We believe that the novelty of this study, therefore, lies in demonstrating how an oncogenic driver mutation can hijack innate immune signaling machinery and redirect it toward tumor maintenance and chemoresistance.

**Comment 2:** In sFigure 1, the authors employed HEK293T cells to evaluate the effects of EGFR hotspot mutations on STING or MAVS-induced IRF3 transactivation. However, the manuscript does not provide any rationale for choosing this cell line, even though HEK293T cells are widely known to lack functional endogenous cGAS-STING signaling and possess attenuated MAVS pathway activity. Without this clarification, readers may be left uncertain about why this particular cell line was chosen and what the specific experimental rationale is.

We thank the reviewer for raising this critical point. We agree that HEK293T cells are not well-suited for evaluating endogenous cGAS-STING or MAVS pathway activity, owing to their limited baseline competence in several innate sensing pathways. In this context, however, that feature is advantageous for our experimental design. We used HEK293T cells as a reductionist reconstitution system in which defined signaling components were ectopically introduced, allowing us to compare the effects of EGFR hotspot mutants on STING- versus MAVS-driven IRF3 transactivation in a controlled background with minimal endogenous pathway interference. Specifically, by co-expressing STING or MAVS together with the IRF3-responsive ISRE reporter, we were able to assess pathway selectivity more directly than would be possible in a background with substantial endogenous signaling noise. This reconstitution strategy is also commonly employed to dissect the mechanistic pathways of STING-TBK1 and MAVS signaling modules. In particular, the conclusions from these assays are not presented in isolation but are supported throughout the manuscript by experiments in EGFR-mutant NSCLC models using endogenous signaling readouts, as well as genetic and pharmacological perturbations. We have now clarified the rationale for using HEK293T cells in the revised manuscript and added the relevant references (Zhang *et al*, 2019; Zhao *et al*, 2019).

**Comment 3:** In Figure 1B, the authors present immunofluorescence images for STING, TBK1, and the corresponding MERGE panels. However, the DAPI (nuclear) channel is not shown anywhere in the figure, despite being required to generate the MERGE images.

We thank the reviewer for noting this issue. We have revised former Fig. 1B (now **Fig.**

**1B and Fig. 1D**) to include the DAPI channel. For other immunofluorescence figures, although DAPI is not always shown as a separate panel because of space limitations, it is included in the merged images, and the corresponding multichannel source images are provided as source data.

**Comment 4:** The authors begin by presenting the results for Figure 1, which is intended to establish the central premise of the manuscript-namely, that EGFR hotspot mutations enhance cGAS-STING-TBK1-IRF3 signaling. However, the immunoblots in Figure 1 uniformly lack any assessment of STING or phospho-STING, which are the core components of this pathway. As a result, although the authors show in Figure 1 that IRF3 transactivation is stimulated by STING rather than MAVS, the current experiments do not distinguish whether EGFR mutations enhance STING-dependent signaling or instead amplify STING-independent TBK1/IRF3 activation. This represents a fundamental mechanistic ambiguity that undermines the central claim of the manuscript. The authors need to include direct measurements of STING and phospho-STING in Figure 1 (and related figures), as well as clarify whether the observed effects are genuinely STING-dependent.

We thank the reviewer for raising this critical point and agree that direct assessment of STING activation is necessary to resolve this ambiguity. In the revised manuscript, we have added STING and phospho-STING immunoblotting to the key experiments supporting our central premise. Specifically, we now include STING and/or phospho-STING readouts together with TBK1 and IRF3 phosphorylation in EGFR-mutant NSCLC cells and wtEGFR H1299 cells under EGFR perturbation **(Fig. 1F, Fig. 1G, Fig. EV1G, and Fig. EV1I)**. Besides, we also provide STING/pSTING immunoblotting in the relevant mechanistic context **(Fig. 4A)**. Across these conditions, mEGFR depletion reduced pSTING in parallel with pTBK1 and pIRF3 in EGFR-mutant NSCLC cells, supporting the conclusion that the observed effects reflect modulation of STING-dependent signaling rather than STING-independent TBK1/IRF3 activation.

**Comment 5:** In Figure 2, the authors conclude that mEGFR "integrates into STING-TBK1 signalosomes" based largely on 2D confocal images showing apparent overlap between STING/TBK1 puncta and EGFR. However, given the crowded perinuclear environment and the proximity of ER, Golgi, and endosomal membranes, 2D projections can be misleading and do not definitively demonstrate that these proteins occupy the same structures in three dimensions. A 3D reconstruction would confirm this result.

We agree that 2D projections in the crowded perinuclear region can be misleading. To address this concern, we performed 3D reconstructions from confocal z-stacks and added orthogonal views (XY, XZ, and YZ), which evidently show that mEGFR overlaps with, or is closely apposed to, STING- and TBK1-positive assemblies in three

dimensions following diABZI stimulation **(Fig. 2D)**. We have also provided the corresponding 3D rendering movies as **Movie EV1**. These additions strengthen the conclusion and reduce the possibility that the observed overlap reflects a projection artifact. Besides, this interpretation is also supported by complementary non-imaging approaches. Co-immunoprecipitation and single-cell proximity ligation assays (PLA) both support mEGFR association/proximity with STING and TBK1, and these interactions are reduced by EGFR kinase inhibition **(Fig. 2E-F; Fig. EV2E-H)**, consistent with a bona fide association with the STING-TBK1 signalosome.

**Comment 6:** The manuscript shows how mEGFR interacts with STING/TBK1, but the fundamental reason why mEGFR, and not wtEGFR, gains this ability is insufficiently explored. The authors suggest increased kinase activity and altered subcellular localization (more endosomal) as potential mechanisms, but a deeper biochemical or biophysical explanation is still lacking.

We thank the reviewer for this insightful comment. We agree that the precise, live-cell-based biochemical basis by which oncogenic EGFR mutants, but not ligand-activated wtEGFR, acquire stable coupling to STING and TBK1 remains to be fully defined, including dedicated future studies of reconstituted systems and quantitative analyses of trafficking and interaction dynamics. In the current study, our data support a working model in which sustained kinase activity, prolonged intracellular residence, and mutant-specific signaling properties enable mEGFR to engage and stabilize STING/TBK1 assemblies, whereas transient ligand-activated wtEGFR does not.

Consistent with this model, immunostaining with organelle markers showed that mEGFR displays increased intracellular localization, with partial association with the Golgi and endosomal compartments compared with wtEGFR **(Fig. EV2B)**, which may increase its dwell time in compartments permissive for STING-TBK1 coupling. In addition, our data suggest that mEGFR-dependent phosphorylation of STING and TBK1 promotes their assembly **(Fig. 3K--L)**, supporting the idea that mEGFR not only localizes to these compartments but also actively stabilizes signaling-competent STING-TBK1 complexes, consistent with a mutant-specific rewiring of EGFR trafficking and interaction properties.

To test whether acute ligand activation and endocytosis of wtEGFR are sufficient to mimic this effect, we stimulated cells with EGF and examined TBK1 and IRF3 activation in the presence or absence of diABZI. Although EGF robustly induced EGFR phosphorylation, it did not further enhance diABZI-induced TBK1 and IRF3 activation **(Appendix Fig. S3A)**. Confocal imaging further showed that EGF induces a redistribution pattern distinct from the prominent cytoplasmic punctate assemblies observed in the mEGFR setting after diABZI stimulation **(Appendix Fig. S3B)**. Together, these findings suggest that coupling of mEGFR to the STING/TBK1 machinery is not simply a consequence of generic ligand-induced EGFR activation or endocytosis, but

instead reflects mutant-specific biochemical and trafficking properties that favor stable assembly of this pathway. We have revised the Discussion accordingly and now explicitly note that the structural, trafficking, and assembly-promoting determinants distinguishing mEGFR from ligand-activated wtEGFR remain to be defined.

**Comment 7:** The DNA repair link remains indirect: While the transcriptomic data (RNA-seq and GSEA) showing downregulation of DNA repair pathways upon TBK1 depletion is compelling, direct mechanistic links between TBK1 and the DNA repair machinery are not fully elucidated. The authors mention potential players, such as PARP, ATM and ISG15, but do not investigate them. How exactly sustained TBK1 activation transcriptionally or post-translationally boosts DNA repair remains uncharacterized.

We thank the reviewer for this insightful comment. In response, we complemented the transcriptomic analyses with direct DNA damage response and homologous recombination readouts in EGFR-mutant NSCLC cells. Upon cisplatin-induced genotoxic stress, TBK1 depletion reduced canonical DDR signaling markers (including pATM(S1981) and pBRCA1(S1524)) and decreased Rad51 accumulation **(Fig. 6D)**. Intriguingly, we observed that at the single-cell level, TBK1 loss markedly reduced Rad51 foci formation in EdU+ cells **(Fig. 6E-F)** and increased DNA damage burden, as reflected by elevated γH2A.X and PARP cleavage **(Fig. 6G-J)**. Importantly, reconstitution of TBK1 in the shTBK1 background restored Rad51 and reduced cisplatin-induced γH2A.X and PARP cleavage **(Fig. EV4D)**, supporting an on-target requirement for TBK1 in these phenotypes. Thus, while our original evidence was largely transcriptomic, the revised data now establish a functional link between TBK1 and DDR/HR competence under cisplatin challenge. We agree, however, that the precisely transcriptional and post-translational effectors connecting TBK1 to the DNA repair machinery remain to be identified and will require further investigations. We have therefore revised the text to avoid overstatement and now present candidates such as PARP, ATM, and ISG15 as plausible future directions rather than mechanistic conclusions of the present study.

**Comment 8:** The study rightly focuses on the cancer cell-intrinsic, non-canonical role of STING-TBK1. However, the title and introduction frame it as rewiring "immune machinery." A discussion on how this tumor-cell-intrinsic hijacking of STING might influence the broader tumor immune microenvironment (e.g., affecting T-cell infiltration or function) would strengthen the study. Exploring the consequences in the tumor microenvironment (TME), using syngeneic or genetically engineered immunocompetent models would dissect how tumor-intrinsic mEGFR-STING-TBK1 signaling shapes the immune landscape.

We thank the reviewer for this constructive suggestion. We agree that describing STING-TBK1 as "immune machinery" could imply a broader analysis of tumor microenvironmental consequences than is addressed in the current study. We have

therefore revised the title and Introduction to more clearly emphasize the tumor cell-intrinsic focus of our work, and we have added a Discussion section outlining the potential implications for the tumor immune microenvironment.

To provide a relevant in vivo context, we performed multiplex immunostaining (TSA) **(Appendix Fig. S4A)** and MHC-I immunohistochemistry **(Appendix Fig. S4B)** in our immunocompetent mEGFR-driven lung tumor model. We found that CD4+ and CD8+ T cells were largely restricted to peribronchial regions, with limited intratumoral infiltration, while PD-L1 staining in the tumor parenchyma was low and tumor-cell MHC-I expression was reduced. This immune-excluded phenotype, which is more evident in mEGFR-only tumors than in mEGFR; Trp53-deficient tumors, is consistent with the known poor responsiveness of EGFR-mutant NSCLC to immune checkpoint blockade. These features also limit the extent to which deeper immune-mechanistic dissection can be meaningfully pursued within the scope of the present study. We now state this limitation and highlight that future studies using syngeneic or genetically engineered immunocompetent models with a more inflamed microenvironment will be important to define how tumor-intrinsic mEGFR-STING-TBK1 signaling shapes the broader immune landscape.

**Comment 9:** Does the uncovered signaling axis lead to the secretion of specific cytokines and chemokines? Similarly, does it alter the ratio of CD8+ T cells to Tregs or myeloid-derived suppressor cells (MDSCs)?

We thank the reviewer for this constructive comment. Our data indicate that activation of the mEGFR-STING-TBK1 axis can induce a canonical STING-associated transcriptional program in tumor cells, including IFNβ in H1975 cells and induction of CXCL10, ISG15, IFITs, and inflammatory transcripts such as IL6 **(Fig. 1J; Fig. EV1J)**. These findings support the idea that this pathway engages cytokine- and chemokine-related programs at the gene-expression level. However, we have not yet performed systematic protein-level secretome profiling, such as multiplex cytokine measurements in conditioned media, across conditions. We have therefore added a discussion of this point as an important future direction.

To begin addressing whether TBK1 perturbation is associated with altered immune-cell composition, we performed flow cytometric profiling in three early-passage air-liquid interface patient-derived organoid (ALI-PDO) cultures with preserved native immune cells (Patient 1: EML4-ALK fusion; Patients 2-3: EGFR exon 19 deletion) under Ctrl, αPD-1, TBK inhibitor (TBKi), or TBKi+αPD-1 treatment. Using the gating strategy shown in **Appendix Fig. S5A**, we quantified major immune subsets within the detectable CD45+ fraction, including T cells (CD3+, CD4+, CD8+), CD11b+ myeloid cells, and Tregs (CD25+Foxp3+ within CD4+). Across the three PDO cultures, TBKi treatment was associated with a reduction in the Treg fraction, whereas the overall CD4/CD8 distribution among CD3+ T cells remained broadly stable; CD11b+ fractions showed

variable changes across PDOs **(Appendix Fig. S5B)**. We note that our current antibody panel identifies CD11b+ myeloid cells but does not resolve canonical MDSC subsets, which would require additional markers.

Together, these data suggest that the mEGFR-STING-TBK1 axis is linked to inflammatory signaling in tumor cells and may also influence local immune composition, although a more complete analysis of secreted factors and myeloid subsets will require future study.

**Comment 10:** The authors need to address the following question: does TBK1 inhibition, by killing tumor cells, indirectly boost antitumor immunity or it induces a direct immunosuppressive effect?

We thank the reviewer for raising this point. We agree that it is important to distinguish the tumor cell-intrinsic effects of TBK1 inhibition from its potential impact on antitumor immunity. In the present study, our data primarily support a tumor cell-intrinsic mode of action: genetic or pharmacological inhibition of TBK1 directly impairs the fitness of EGFR-mutant NSCLC cells, compromises DNA damage tolerance, and enhances cisplatin sensitivity, with increased apoptosis observed both in vitro and in vivo. These findings indicate that the therapeutic effects observed in our models can be explained, to a substantial extent, by direct tumor cell killing and chemosensitization.

At the same time, we agree that increased tumor cell death could secondarily enhance antitumor immunity, for example, through the release of tumor antigens or the generation of damage-associated signals. Conversely, TBK1 inhibition may also exert direct effects on immune cells, which can be immunostimulatory or immunosuppressive depending on the context. A clear distinction between these possibilities will require dedicated immune profiling and cell-type-specific perturbation strategies. We have therefore added a brief Discussion paragraph to specifically acknowledge this point, place our findings in the context of recent work linking TBK1 to immunotherapy response(Sun *et al*, 2023), and outline this as an important direction for future study.

**Comment 11:** Specificity to mEGFR: While the data support the role in mEGFR-driven NSCLC, it would be important to know if other oncogenic kinases (e.g., ALK and ROS1 fusions) can similarly hijack the STING pathway, or if this is a unique feature of mEGFR.

We thank the reviewer for this intriguing point, which we are assessing to determine whether this phenomenon extends beyond mEGFR. To address this, we examined two additional oncogenic RTK fusions, EML4-ALK and CD74-ROS1, using the ISRE-luciferase reconstitution system as in Fig. **EV1A-C**. Intriguingly, EML4-ALK with STING significantly enhanced STING-dependent ISRE reporter activity, to a degree comparable to that

observed with mEGFR, whereas CD74-ROS1 did not enhance STING-driven reporter activation under our assay conditions **(Fig. EV1D)**. Expression of STING and the oncogenic kinases was confirmed by immunoblotting **(Fig. EV1E)**. These findings suggest that engagement of the STING-TBK1 axis by tyrosine kinases is not unique to mEGFR, but may be shared by a subset of oncogenic RTKs with similar cellular compartments, while not representing a universal property of all RTK fusions.

**Reviewer #3:**

In their article 'Oncogenic EGFR rewires STING-TBK1 immune machinery by tyrosine phosphorylation to license DNA damage tolerance', Shen et al. describe a new intersection between STING-dependent signalling and mutant EGFR. They provide evidence that mEGFR associates with both STING and TBK1, and that it can support TBK1-dependent signalling. Some of the data also suggest that mEGFR is required for STING/TBK1-dependent signalling, and that it may rewire the transcription outputs. Ultimately, they suggest that in mEGFR cell lines, TBK1 becomes important for cell proliferation, and that loss of TBK1, or its inhibition can synergise with cisplatin treatment, in cell lines as well as in murine models.

Whilst the data are intriguing and abundant, several key points are not clear. The mechanistic basis is not described in sufficient detail, and some discrepancies in the data exist that would need reconciliation. The manuscript also relies a bit too much on the expression of exogenous proteins, rather than analysis of endogenous proteins.

We sincerely thank the reviewer for the thoughtful and constructive evaluation of our work. We appreciate the reviewer's recognition of the intriguing link between mutant EGFR and STING-TBK1 signaling, as well as the potential significance of TBK1 dependency in mEGFR-driven tumors. We also appreciate the concerns regarding mechanistic depth, data reconciliation, and the reliance on exogenous expression systems. In the revised manuscript, we have addressed these points through substantial additional experiments and analyses, which have strengthened the mechanistic basis of the study and improved support for its physiological relevance.

Major points

**Comment 1:** It is unclear how mEGFR intersects with STING/TBK1. In some experiments it looks like it supports signalling following STING agonist treatment, but in other experiments, it seems like there is strong baseline signalling in the absence of other stimuli. For example, the microscopy (Fig. 2A-C) suggests that diABZI addition is required for the co-localisation between mEGFR and STING, but then in the IPs (Fig. 2D for example), no stimulation is required. In Fig. 1G, diABZI addition is required to see an impact of mEGFR on TBK1ph, but in Fig. 3B,E,H, this is not the case. In Fig. 4C, no stimulation is required for mEGFR to regulate TBK1 stability. In the colony formation/viability experiments, no external cGAS or STING stimuli are added.

Furthermore, it seems like in mEGFR cell lines, mEGFR is required for STING-dependent signalling (for example, Fig. 2B, EGFR inhibition prevents STING relocalisation to the Golgi), raising the question, how STING can signal in cells that do not express mEGFR. So overall, it isn't clear to me what the actual message is. Is it that mEGFR promotes STING signalling? Or that it somehow is required for STING signalling? Finally, the authors usually only look at one time point, but all that might occur in mEGFR cells is that kinetics are modified.

We thank the reviewer for this important comment. We agree that the original manuscript did not clearly distinguish basal association from stimulus-induced activation, nor did it sufficiently clarify whether mEGFR promotes STING signaling or is required for it. We have revised the text accordingly and added additional time-course analyses to address these points.

**(1)** Microscopy appears to suggest that diABZI is "required" for mEGFR co-localisation with STING/TBK1, whereas co-IP detects interaction without stimulation.

These observations reflect different readouts. Co-immunoprecipitation detects baseline physical association between mEGFR and STING/TBK1 in unstimulated EGFR-mutant cells **(Fig. 2E-F)**, whereas confocal microscopy primarily visualizes punctate assemblies that become prominent after STING agonism. Thus, diABZI enhances visible signalosome assembly rather than generating a de novo interaction. Consistently, our single-cell proximity analyses support detectable basal EGFR-TBK1 and EGFR-STING association and show that mEGFR kinase inhibition (WZ4002) reduces this proximity **(Fig. 2B-C; Fig. EV2C-D; Fig. EV2E-H)**. To avoid misinterpretation from 2D projections, we also added 3D reconstructions with orthogonal views and a corresponding movie **(Fig. 2D; Movie EV1)**.

**(2)** Why is STING agonism required to see an impact of mEGFR on pTBK1 in some experiments (e.g., Fig. 1), but not in others (e.g., Fig. 3/4)?

We agree that the use or absence of exogenous stimulation can appear inconsistent across figures, and we have clarified this in the revised text. The key issue is that different panels use different systems and activation modes. In NSCLC cells (Fig. 1), we use defined STING/cGAS agonists (diABZI/cGAMP/DNA) to create an inducible signaling window for assessing STING-dependent pathway activation (pTBK1/pIRF3). In the HEK293T reconstitution assays **(Fig. 3B, H)**, TBK1 overexpression itself is sufficient to generate an activated state, functionally analogous to agonist-induced activation. Separately, the pY100-based experiments **(Fig. 3E)** assess mEGFR-dependent tyrosine phosphorylation of TBK1/STING and therefore detect a basal modification signal in H1975 cells that is reduced by mEGFR inhibition and is weak or absent in wtEGFR H1299 cells. We now state more clearly that mEGFR-dependent coupling is detectable at baseline, whereas its effect on signaling output is most evident under activated conditions.

**(3)** Why do colony formation/viability assays show TBK1/STING dependence without

adding exogenous cGAS/STING stimuli?

We agree that this required clearer explanation, and we have revised the text to distinguish two contexts in which we interrogate the pathway. In one context, we use defined STING/cGAS agonists to assess acute canonical STING activation (pSTING-pTBK1-pIRF3 and ISG/IFN outputs). In the colony formation/viability assays, however, we are probing a different biological output: the tumor cell-intrinsic pro-survival and DNA damage tolerance functions of the mEGFR-STING-TBK1 axis. These phenotypes do not require exogenous agonist and become particularly evident under cisplatin-induced genotoxic stress, where TBK1 supports DNA damage repair capacity and survival.

**(4)** Is mEGFR required for STING signaling, or that it promotes/potentiates it? How can STING signal in cells without mEGFR?

We do not intend to imply that STING intrinsically requires mEGFR to function. Rather, our data support that oncogenic EGFR acts as a context-specific amplifier and stabilizer of the STING-TBK1 axis in EGFR-mutant NSCLC. Consistent with prior work showing that wtEGFR can influence activated STING trafficking and tune downstream IRF3/IFN outputs without acting as a universal on/off switch (Wang et al., EMBO J, 2020). In our setting, however, oncogenic EGFR strengthens STING/TBK1 association and stabilizes activated TBK1, thereby enhancing the magnitude and duration of pathway output under activated conditions.

**(5)** Could the phenotype reflect altered kinetics rather than absolute differences?

We agree that kinetics are important. We therefore included finer time-course analyses **(Fig. 4A; Appendix Fig. S2A).** Across these time courses, the difference between mEGFR Dox- and Dox+ conditions is most pronounced at approximately 1 h after diABZI stimulation, which is why this time point was used for several mechanistic assays to maximize dynamic range. Importantly, our data support a model in which mEGFR affects both the amplitude and the decay phase of pathway output: mEGFR shows detectable basal association with STING/TBK1, and upon STING activation, mEGFR-dependent tyrosine phosphorylation strengthens pathway coupling and stabilizes activated TBK1 by limiting K48-linked ubiquitination and proteasomal turnover. Consistent with this stabilization model, the impact of mEGFR becomes particularly evident as TBK1 would otherwise decline, resulting in stronger and more sustained pathway output.

Taken together, the data support a model in which mEGFR establishes baseline coupling to STING/TBK1 and, upon agonist stimulation or genotoxic stress, facilitates signalosome organization and sustains TBK1 activity by stabilizing activated TBK1. STING can signal without mEGFR, but mEGFR provides a tumor-specific input that increases the magnitude and duration of pathway output and supports tumor-cell fitness. The corresponding Results and Discussion text have been revised accordingly; we also added 3D reconstruction/Movie EV1 and finer time-course analyses **(Fig. 2D;**

**Movie EV1; Fig. 4A; Appendix Fig. S2A)**.

**Comment 2:** The molecular mechanism by which the authors propose that mEGFR regulates STING/TBK1 (direct interaction) is not proven by the experiments, which rely on pulldowns with overexpressed proteins. These types of experiments to do not report on direct molecular interactions. To validate their model, recombinant proteins would be needed.

We agree that pulldown assays with overexpressed proteins do not establish a direct molecular interaction between mEGFR and STING/TBK1. To address this point, we attempted to develop recombinant protein-based biochemical assays using purified mEGFR kinase domain together with full-length and truncated TBK1 proteins. Although the mEGFR kinase domains could be obtained, we were unable within the revision period to produce TBK1 proteins of sufficient quality to yield a rigorous direct binding analysis. We therefore agree that definitive demonstration of direct binding will require additional future work with purified reconstituted systems. Besides, we have performed PLA-based proximity analysis **(Fig. EV2E-H)** and colocalization analysis using 3D imaging **(Fig. 2D)** . These complementary cellular approaches, together with IF assays and co-immunoprecipitations, all support the association and functional interaction between mEGFR and the STING signalosome. We therefore have toned down our original wording and, in the revised manuscript, interpret the data more cautiously and conclude that mEGFR associates with, or is coupled to, the STING-TBK1 complex in cells rather than binding it directly.

**Comment 3:** In many experiments, the readouts are not benchmarked against bona fide cGAS-STING stimuli. For example, the initial HEK293T experiments (Fig. S1AB) look at STING expression in HEK293T cells, which leads to weak autoactivation, which is stimulated by mEGFR. However, the output is not benchmarked against what could be achieved by co-expressing cGAS with STING, whilst also providing a DNA stimulus, or - alternatively, what could be achieved by addition of diABZI. This kind of control is important, as it allows the true level of impact of mEGFR to be assessed.

We thank the reviewer for this helpful suggestion and agree that benchmarking the HEK293T reconstitution assays against bona fide cGAS-STING activation is important for interpreting the magnitude of the mEGFR effect. We therefore added benchmarking controls in 5×ISRE-luc reporter assays comparing STING expression alone, STING plus mEGFR, and canonical pathway activation induced by cGAS coexpression or by established STING stimuli, including diABZI, HT-DNA, and the mtDNA-release-inducing AQS cocktail. As shown in **Appendix Fig. S1A-B**, mEGFR significantly enhances STING-driven reporter activity above baseline autoactivation, comparable to the dynamic range defined by these canonical activation conditions. These controls provide an appropriate reference for effect size and support our

conclusion that mEGFR functions as an amplifier of STING-dependent signaling.

**Comment 4：** The microscopy experiments (Fig. 1B and 2A-C and Fig. S2) need to be quantified. The authors also make too sweeping statements about the localisation patterns. For example, in Fig. 1B, the differences of with and without WZ4002 are extremely subtle, especially for TBK1, but are treated as highly different in the text. Also, with regards to the relocalisation of mEGFR following diABZI addition, does this depend on STING?

Thanks. In the revised manuscript, we have added quantitative analyses for the relevant imaging panels. For the H1975 immunofluorescence experiments in Fig. 1, we now quantify the single-cell fluorescence heterogeneity of TBK1 and STING using the coefficient of variation (CV), which captures the shift from diffuse to punctate distributions **(Fig. 1C and 1E)**. For the colocalization analyses in Fig. 2, we now provide single-cell Manders overlap measurements together with summary statistics **(Fig. 2B-C; Fig. EV2C-D)**. We have also revised the text throughout to avoid overstatement. In particular, the effect of WZ4002 in Fig. 1 is now described more cautiously as reducing punctate organization and increasing diffuseness, rather than implying a strong binary relocalization event.

Regarding whether mEGFR relocalization after diABZI depends on STING, we agree that this is an important mechanistic question and performed an additional imaging experiment in H1975 shCtrl and H1975 shSTING cells. In this setting, the punctate intracellular redistribution of EGFR observed after diABZI treatment in shCtrl cells appeared less evident in STING-depleted cells **(Appendix Fig. S6)**, consistent with the possibility that efficient mEGFR enrichment in these assemblies is facilitated by STING. Because this experiment was exploratory and not pursued quantitatively, we present it as supportive rather than definitive evidence. Accordingly, we have revised the manuscript to avoid claiming that diABZI-induced mEGFR relocalization is formally proven to be STING-dependent, and instead state more conservatively that mEGFR becomes enriched in STING/TBK1-positive assemblies upon agonist stimulation.

**Comment 5：** Quantifications should also be added for other experiments (basically all, but just to name a few, Fig. 4A,C,F)

In the revised manuscript, we have added densitometric quantification for essentially all immunoblot-based experiments, including the examples noted by the reviewer (Fig. 4A, 4C, and 4F), as well as the corresponding WB and IP/co-IP panels throughout the study. For IP/co-IP experiments, the co-immunoprecipitated signal was normalized to the corresponding immunoprecipitated bait protein in the same lane. We now state this normalization strategy specifically in the Materials and Methods.

**Comment 6:** There are some inconsistencies in the data, and some data that do not seem to support the authors' conclusions. For example, (a) in Fig. 1F, I do not see any obvious difference between cells with and without mEGFR as far as pTBK1 and pIRF3 are concerned, especially once differential loading is accounted for. (b) I am not convinced that the data as shown support the idea that TBK1 levels are regulated by mEGFR (see for example Fig. 4A). (c), the impact of TBK1 mutations on expression levels seen in Fig. 4J is not recapitulated in the HEK system (Fig. 3C and 3H and S3A) and also not in the H1975 system (Fig. 4A). (d) Fig. 5I and Fig. 5J seem to show different effects for Tet-shCtrl Dox+.

We thank the reviewer for these observations and agree that several conclusions required clearer presentation and more precise wording. We have therefore revised both the figures and the text accordingly.

**(a)** in Fig. 1F, I do not see any obvious difference between cells with and without mEGFR as far as pTBK1 and pIRF3 are concerned, especially once differential loading is accounted for.

We agree that the original Fig. 1F did not show the effect clearly, especially at early time points. Our conclusion is not that mEGFR is absolutely required for STING signaling, but that it enhances pathway output. To clarify this more directly, we repeated the experiment using HT-DNA stimulation and an extended time course. In the revised **Fig. 1G**, EGFR depletion reduces pSTING, pTBK1, and pIRF3 more clearly, especially at 4 h. We also added a diABZI validation experiment in Fig. EV1G.

**(b)** I am not convinced that the data as shown support the idea that TBK1 levels are regulated by mEGFR (see for example Fig. 4A).

We thank the reviewer for this point and agree that our original wording was too broad. Our intended conclusion is not that mEGFR broadly elevates basal TBK1 abundance under all conditions, but rather that mEGFR stabilizes activated TBK1 during the turnover phase.

In the revised manuscript, we therefore interpret **Fig. 4A** more specifically as showing that, following STING agonist stimulation, TBK1 undergoes a time-dependent decline and that induction of mEGFR attenuates this decline while maintaining stronger pathway output. We have also added a finer early time course **(Appendix Fig. S2A)**, which supports the idea that the key difference emerges during the decay of the activated pool, consistent with the cycloheximide chase, ubiquitination, and mutant data in Fig. 4.

**(c)** the impact of TBK1 mutations on expression levels seen in Fig. 4J is not recapitulated in the HEK system (Fig. 3C and 3H and S3A) and also not in the H1975 system (Fig. 4A).

TBK1 mutant behavior reflects distinct experimental purposes. Y→F mutants were used in HEK293T phosphorylation mapping assays to interrogate phosphorylation-site

dependence, whereas the Y→E (2YE) mutant was used in rescue/stability experiments as a phosphomimetic to assess phosphorylation-dependent stabilization. We have revised the text to clearly distinguish these purposes and ensure that these assays are not interpreted as expecting identical expression-level phenotypes across systems.

**(d)** Fig. 5I and Fig. 5J seem to show different effects for Tet-shCtrl Dox+.

We agree that the Tet-shCtrl Dox+ group shows a modest reduction in tumor growth curve (Fig. 5I) that is less evident in endpoint tumor weight (Fig. 5J). We interpret this as reflecting the different nature and resolution of the readouts, with tumor volume estimated longitudinally by caliper measurement and tumor weight as a direct endpoint. A minor effect of doxycycline cannot be excluded. To address this, we added the Tet-shCtrl group to the in vitro apoptosis analysis **(Fig. 5D)**, where doxycycline alone did not induce substantial apoptosis, whereas TBK1 depletion did.

**Comment 7:** With regards to the viability impact of TBK1 loss on mEGFR cell lines, DepMap does not seem to show these effects. Could the authors do a wider analysis of DepMap data, cross-referencing with EGFR status to gain broader support of their hypothesis? Similarly, could TCGA data be analysed? The prediction would be that there should be strong selection to maintain cGAS/STING/TBK1 in mEGFR cancers.

We thank the reviewer for this valuable suggestion. We examined publicly available DepMap data with attention to EGFR status. However, within the limited set of relevant NSCLC cell lines available for this analysis, we did not observe a consistent association between EGFR genotype and TBK1 dependency. We therefore consider the current DepMap dataset insufficient to draw a firm conclusion on this point and have not used it as a primary line of evidence.

To provide a complementary clinical context, we added an exploratory analysis of the TCGA PanCancer Atlas. Across 1,053 NSCLC cases (566 LUAD and 487 LUSC), homozygous deletion of CGAS, STING1, or TBK1 was uncommon among tumors harboring EGFR hotspot mutations **(Appendix Fig. S8A)**. Although descriptive, this is consistent with retention of the cGAS-STING-TBK1 axis in mEGFR-associated NSCLC.

We also included non-EGFR-stratified TCGA-LUAD analyses showing that TBK1 expression is elevated in tumors relative to normal lung and that higher TBK1 expression is associated with poorer survival **(Appendix Fig. S8B-C)**. We note, however, that these analyses provide a broader clinical context rather than direct evidence for EGFR genotype-specific dependency.

**Comment 8:** None of the IP experiments contain negative controls (proteins that shouldn't be pulled down such as actin or tubulin), which can be used to assess how clean the pulldowns are. The IPs are also a bit confusing for other reasons. STING is

degraded upon activation, and it is therefore difficult to see how a stable complex between mEGFR, STING and TBK1 could persist for prolonged periods of time. Furthermore, Fig. 2F suggests interactions with both STING and TBK1, but then in Fig. 2G interactions with TBK1 are observed in the absence of STING. Is the idea that mEGFR interacts with TBK1, and this bridges to STING? But again, such claims would have to be backed up by more detailed biochemistry.

To address the first point, we added EGFR immunoprecipitation control in H1975 cells using proteins not expected to co-precipitate with EGFR. In this experiment, TBK1 and STING were specifically detected in the EGFR IP, whereas cGAS and GAPDH were not **(Appendix Fig. S8A)**, supporting the specificity of the pulldown.

We also agree that our original wording may have implied an overly stable mEGFR-STING-TBK1 complex. However, we do not propose that a single invariant trimeric complex persists throughout STING activation. Rather, our data support a more dynamic model in which mEGFR associates with this pathway at baseline and facilitates coupling to STING and TBK1-containing signalosomes, whose composition likely changes during activation, trafficking, and turnover.

To further clarify the relationship between EGFR, STING, and TBK1, we added a dependency analysis in H1975 cells. EGFR-TBK1 association remained detectable after STING depletion, whereas EGFR-STING association was reduced after TBK1 depletion **(Appendix Fig. S8B)**. We interpret these data cautiously as consistent with TBK1 serving as a central coupling node in this pathway, while avoiding a stronger claim that TBK1 definitively acts as a simple linear bridge. Accordingly, we have revised the text to distinguish the experimental observations from mechanistic inference and to avoid overstatement regarding direct or stable complex formation.

**Comment 9:** In general, the manuscript relies too much on overexpression of transgenes, which is utilised in almost every experiment. Obviously for introducing mEGFR in EGFR wt cells, this is acceptable, but I don't quite understand why impact on wt TBK1 and STING usually is not assayed with the endogenous proteins but with tagged exogenously expressed ones (for example, Fig. S4).

Thanks for the suggestions. Our intention for the cellular reconstitution system was not to base the main conclusions on transgene overexpression. Rather, these experiments were used primarily as mechanistic tools in tractable systems, for example, to map tyrosine sites, compare phospho-deficient and phosphomimetic mutants, or test reporter responses in a low-background setting.

The central conclusions of the study are instead supported in EGFR-mutant NSCLC models using endogenous or semi-endogenous settings. Specifically, the effects on pathway activation, complex formation, and TBK1 regulation are supported by analyses of endogenous pSTING/pTBK1/pIRF3, endogenous co-immunoprecipitation,

knockdown/rescue experiments, and functional studies in NSCLC cells, patient-derived PDOs, and in vivo murine models. In this context, the HEK293T overexpression assays primarily served to generate and refine mechanistic hypotheses, which were subsequently examined in disease-relevant systems.

We agree, however, that this distinction was not sufficiently clear in the original manuscript. We have therefore revised the text and figure legends to more explicitly distinguish overexpression-based mechanistic experiments from endogenous validation experiments, and we have toned down the wording where appropriate to avoid over-interpreting observations made in transfected systems.

**Comment 10:** In most shRNA experiments, rescue experiments are not performed.

In the revised manuscript, we added two independent rescue experiments to support the specificity of the shTBK1 phenotypes. First, in the apoptosis setting, re-expression of shRNA-resistant TBK1-Myc in shTBK1 H1975 cells attenuated the increase in cleaved PARP induced by TBK1 knockdown **(Fig. EV3E)**. Second, in the DNA damage setting, reconstitution of TBK1-Myc in shTBK1 H1975 cells restored RAD51 and reduced cisplatin-induced γH2AX accumulation and PARP cleavage **(Fig. EV4D)**. Together, these rescue experiments support that the major phenotypes observed after TBK1 knockdown are on-target. We note that rescue was not performed for every individual shRNA experiment. However, these added datasets address specificity at the two main functional levels emphasized in the manuscript, namely tumor-cell survival and DNA damage repair.

**Comment 11:** The mRNAseq analysis is rather confusing. Why are inflammatory genes up when TBK1 is lost? This is the opposite of what one would expect.

Thanks for the suggestion. We do not interpret the upregulation of inflammatory gene sets after TBK1 loss as evidence of enhanced canonical STING-TBK1-IRF3 signaling. Rather, we believe these changes most likely reflect secondary stress-associated transcriptional responses induced by TBK1 depletion. In our dataset, the enriched programs are broader inflammatory signatures rather than a selective increase in the canonical IRF3-dependent branch(Abe *et al*, 2013; Zhang *et al*, 2022). One plausible explanation is that loss of TBK1 impairs genome-maintenance and survival pathways, thereby increasing cellular stress and triggering compensatory inflammatory transcriptional programs(McCool & Miyamoto, 2012; D *et al*, 2014; Bakhoum & Cantley, 2018). We agree that the precise mechanism underlying this shift is not resolved in the present study. We have therefore revised the text to discuss these findings more cautiously and to avoid over-interpreting them as a direct signaling consequence of TBK1 loss.

**Comment 12:** The dose-response curves (Fig. S6H-K and Fig. 7B) do not have statistical

tests.

We thank the reviewer for this helpful suggestion. In the revised manuscript, the dose-response curves in **Fig. EV4N-O** and **Fig. 7B** were fitted by nonlinear regression and compared using an extra sum-of-squares F test. The corresponding statistical method is now indicated in the figure legends and Methods, and the resulting *P* values have been added to the revised figures.

**Minor points:**

**Comment 13:** In Fig. 1D, the use of poly(dA:dT) as a cGAS stimulant is odd, as this was indicated to trigger Rig-I dependent signalling (PMID 19609254). The authors should use plasmid DNA or herring testes DNA instead.

Though Poly(dA:dT) transfection is a documented stimulation of cGAS, it may also engage additional nucleic acid-sensing pathways beyond cGAS-STING. To address this concern, we repeated the key experiment using herring testes DNA (HT-DNA) as an alternative DNA stimulus. As shown in the revised **Fig. 1G**, HT-DNA induced STING pathway activation, and this response was reduced by EGFR depletion, consistent with our conclusion that mEGFR enhances the STING branch.

**Comment 14:** The text describing Fig. 1H and S1E says that interferon levels were analysed but these are not shown in the figures.

Thanks. In the revised manuscript, we now state more precisely that IFNB1 induction was analyzed and shown in H1975 cells **(Fig. 1J)**, whereas the HCC827 data primarily show induction of ISGs and inflammatory transcripts, including ISG15, IFIT1, CXCL10, and IL6 **(Fig. EV1J)**, probably through IFNα response. We have revised the text accordingly.

**Comment 15:** Fig. 6I/J and and 7A: Please show the full y axis!

We have revised the affected plots to display the full y-axis range. In the revised manuscript, this change has been applied to Fig. EV4L-M (formerly Fig. 6I/J) and Fig. 7A.

**Comment 16:** Please keep colours consistent between Fig. 2A and 2B/C.

We thank the reviewer for this suggestion. To ensure consistent channel assignment and color presentation across the figure, we re-acquired **Fig. 2A** using the same spinning-disk confocal imaging platform as for **Fig. 2B-C** and updated the figure layout accordingly.

**Comment 17:** Lines 205-206: 'mEGFR coexpression revealed a notable reduction in pY100 signals in TBK1 Y577F, Y677F, or Y577F/Y677F mutants' is poorly expressed and ambiguous in its meaning.

Thanks. Our intended meaning was that, compared with WT TBK1, the Y577F, Y677F, and Y577F/Y677F mutants exhibited reduced pY100 reactivity when co-expressed with mEGFR, consistent with Y577 and Y677 contributing to mEGFR-dependent tyrosine phosphorylation of TBK1. We have revised the sentence accordingly for clarity.

**Comment 18:** I cannot find the data for Lines 301-303 ('Co-immunoprecipitation assays indicated that cisplatin facilitated the formation of EGFR-STING-TBK1 tripartite complexes, thus potentially linking mEGFR to STING signalosome activation and drug resistance.')

We thank the reviewer for identifying this inconsistency. During figure reorganization in revision, the co-immunoprecipitation experiment related to cisplatin-enhanced EGFR-STING-TBK1 association was not retained in the revised figure set, but the corresponding statement was inadvertently left in the Results text. We apologize for this oversight. To avoid unsupported interpretation, we have removed this sentence from the revised manuscript and ensured that the text is fully aligned with the data presented.

**Comment 19:** Please avoid words like "eradicate" and "impressive" when describing effects.

We thank the reviewer for this suggestion and have revised the manuscript throughout to replace subjective terms such as “eradicate” and “impressive” with more measured, data-based descriptions, as appropriate.

**References in the Response:**

Abe T, Harashima A, Xia T, Konno H, Konno K, Morales A, Ahn J, Gutman D & Barber GN (2013) STING recognition of cytoplasmic DNA instigates cellular defense. *Mol Cell* 50: 5-15

Bakhoum SF & Cantley LC (2018) The Multifaceted Role of Chromosomal Instability in Cancer and Its Microenvironment. *Cell* 174: 1347-1360

D K, Wj C, J C, Ka E, Pm G, Al B & Jb S (2014) Interplay between DNA repair and inflammation, and the link to cancer. *Crit Rev Biochem Mol Biol* 49

Hong C, Schubert M, Tijhuis AE, Requesens M, Roorda M, van den Brink A, Ruiz LA, Bakker PL, van der Sluis T, Pieters W, *et al* (2022) cGAS-STING drives the IL-6-

dependent survival of chromosomally instable cancers. *Nature* 607: 366-373

McCool KW & Miyamoto S (2012) DNA damage-dependent NF-κB activation: NEMO turns nuclear signaling inside out. *Immunol Rev* 246: 311-326

Sun Y, Revach O-Y, Anderson S, Kessler EA, Wolfe CH, Jenney A, Mills CE, Robitschek EJ, Davis TGR, Kim S, *et al* (2023) Targeting TBK1 to overcome resistance to cancer immunotherapy. *Nature* 615: 158-167

Zhang C, Shang G, Gui X, Zhang X, Bai X-C & Chen ZJ (2019) Structural basis of STING binding with and phosphorylation by TBK1. *Nature* 567: 394-398

Zhang D, Liu Y, Zhu Y, Zhang Q, Guan H, Liu S, Chen S, Mei C, Chen C, Liao Z, *et al* (2022) A non-canonical cGAS-STING-PERK pathway facilitates the translational program critical for senescence and organ fibrosis. *Nat Cell Biol* 24: 766-782

Zhao B, Du F, Xu P, Shu C, Sankaran B, Bell SL, Liu M, Lei Y, Gao X, Fu X, *et al* (2019) A conserved PLPLRT/SD motif of STING mediates the recruitment and activation of TBK1. *Nature* 569: 718-722

## 1st Revision - Editorial Decision 4th May 2026

Dear Dr Xu,

Thank you for submitting your manuscript EMBOJ-2025-122789R to The EMBO Journal, as well as for your patience with our feedback at this time of the year. Your manuscript was sent back to the three reviewers, and we have now received rereports from two of them, which I enclose below. Please note that while referee #2 was not able to look back into the amended work at this time, we have assessed your response to the critique raised and found it to be reasonably addressed. The other two experts are supportive of the revised study and encourage publication, pending satisfactory further amendments.

Given the referees' overall positive recommendations, I would like to invite you to submit a final, revised version of the manuscript, addressing the remaining comments of reviewer #3 regarding additional experimental controls required (pts. 2,4,8), experimental data statistics annotation (pt.1) as well as adequate revision of the wording and claims made (pts. 3,5,9).

Please submit the revised version of the manuscript using the link enclosed below, addressing the advisor's comments.

Thank you again for giving us the chance to consider your manuscript for The EMBO Journal, I look forward to hearing from you and receiving your final revised version of the manuscript.

Kind regards,

Daniel Klimmeck

Daniel Klimmeck PhD
Senior Editor
The EMBO Journal

Please remember: Digital image enhancement is acceptable practice, as long as it accurately represents the original data and conforms to community standards. If a figure has been subjected to significant electronic manipulation, this must be noted in the figure legend or in the 'Materials and Methods' section. The editors reserve the right to request original versions of figures and the original images that were used to assemble the figure.

We realize that it is difficult to revise to a specific deadline. In the interest of protecting the conceptual advance provided by the work, we recommend a revision within 3 months (2nd Aug 2026). Please discuss the revision progress ahead of this time with the editor if you require more time to complete the revisions.

------------------------------------------------

Referee #2:

The revised form of the manuscript satisfies my previous concerns and it is now ready for publication in the Journal.

Referee #3:

In their revised article 'Oncogenic EGFR rewires STING-TBK1 immune machinery by tyrosine phosphorylation to license DNA damage tolerance', Shen et al. provide extra evidence to support their hypothesis of mEGFR regulation of STING/TBK1. As before, whilst the data are very abundant, and the authors did a commendable amount of work, the manuscript still suffers from some important weaknesses, as outlined below. Where possible, I will give the authors the option to address the points by re-phrasing aspects of the manuscript, or removing data, rather than performing extra experiments.

Major points
1. I had previously requested quantifications of experiments, which the authors interpreted as giving numerical values for individual experiments. What was also implied was that the authors should be more transparent about how often experiments were repeated, and how reproducible the results were. I cannot find any indication on the figures or figure legends for how often

experiments were performed. Especially for experiments that are analysed by WBs, and colony formation assays is this necessary. Please provide this information, and ideally also provide bar graphs showing quantifications from multiple experiments.
2. With regards to my previous Major Point 8 (lack of negative controls in IP experiments), the authors just performed one experiment with added controls (Fig. S8A, not referenced in the text). This is obviously not sufficient. Every IP experiment needs to have negative controls, otherwise their value cannot be assessed.
3. Regarding the immunofluorescence analysis of co-localisation (Fig. 2A-D; Fig. EV2AB): I had previously assumed that these images represent single confocal slices, but the authors wording in the revised manuscript (lines 200-201,'We reconstructed confocal z-stacks in three dimensions (3D) to exclude possible artefacts in 2D projections.') make it sound like projections were used. This is obviously not valid, and the two 3D reconstructed images shown in Fig. 2D are not sufficient to resolve this. Overall, however, I do not think that extra experiments are required. I would accept it if the authors changed all mentions of "colocalization" to 'the two proteins occupying similar intracellular areas, with a potential of partial co-localisation', as this describes the data more accurately.
4. Furthermore, with regards to co-localisation, the PLA experiments lack the fundamental controls in which PLA is performed with samples in which first one of the two antibodies, and then the other, has been replaced with IgG of the correct isotype/species. PLA is a notoriously artefact-prone procedure, and without this control, specificity cannot be assessed. I would, however, accept it if the authors removed the PLA data instead of adding extra experiments.
5. The wording and interpretations by the authors still is frequently somewhat disconnected to the actual data, and should be toned down. (a) In the abstract, lines 50-52, and the results section (lines 379-383), the appearance is given that TBK1 loss/inhibition has a dramatic and statistically significant effect. But in actuality, the data are more nuanced, with large variability (especially between organoids, Fig. 7AB, but also between cell lines, Fig. EV4L-O), and generally mild effects (max 2-3 fold sensitivity difference). Furthermore, given that DepMap does not show a generality of mEGFR cell lines being sensitive to TBK1 loss also indicates that caution is the appropriate way forward. (b) In the Discussion, lines 467-469, again the language seems to be too grand when the data from Fig. 3K-L are somewhat subtle. (c) In the Discussion (line 569), the authors describe their findings as a paradigm shift. Please remove this.
6. The benchmarking of mEGFR effects on STING dependent signalling (lines 139-142) need to include the condition of mEGFR and STING co-expression together with diABZI or mEGFR expression together with STING/cGAS co-expression. Also, this data cannot be buried in the supplement, as it is important to assess the actual impact of mEGFR (which appears to be consistent and therefore likely to be important despite appearing to be relatively mild - Fig. S1A).
7. Whilst I appreciate that generally, HEK293 experiments are followed up with more relevant cell lines, there is still a set of data that is only performed in HEK293 cells, relating to the more detailed analysis of the tyrosine mutants (Fig. 3H-L).
8. The findings on TBK1 regulating DNA damage processing are very unexpected, and more evidence is required therefore. In my previous review, I had requested rescues of shRNA experiments, but the authors only partially did this (compare the assays in Fig. 6D-J with the single rescue experiment, Fig. EV4D). Furthermore, that single rescue experiment actually shows only subtle phenotypes of TBK1 loss in contrast to the experiments of the main figure). Therefore, the authors would either need to perform rescues of the other assays as well, or remove the DNA response aspect from the manuscript. Their claims on TBK1 impacting cisplatin would obviously have to be much more subtle in that case (which they should be in any case, see Major Point 5).
9. The model figure must indicate that the interpretation of a direct complex between TBK1, STING and mEGFR is no proven by the data (none of the experiments shows a direct interaction, all the interactions the authors observed could be from bridging proteins. Direct interaction could only be inferred by recombinantly expressed and biochemically purified proteins.) At the very least the authors need to add something like a question mark next to the words 'complex formation'. In light of this, the words in the Discussion (lines 458-459) "integrate into STING signalosomes" should also be replaced with "associate with STING".
10. Fig. S7 should be removed. Fig. S7A seems to be misleading, as only a few selected patient samples from over a 1000 in total are shown. Fig. S7BC do not show data stratified by mEGFR expression, and the impact of TBK1 expression could therefore be mediated by completely different mechanisms than the ones the authors are studying.

Minor point
1. The wording of line 446-448 makes it seem like there is something special about breast cancer with regards to cGAS-STING, but this has not been established. It just happened that the studies the authors cite used breast cancer cell lines. But they did not show that the effect is limited or generally applicable to breast cancer.

**Response to the reviewers (EMBOJ-2025-122789):**

We thank the editor and reviewers for their continued efforts in evaluating our manuscript and appreciate the opportunity to submit a final revised version that addresses the remaining comments.

In this revision, we have focused on the issues specifically highlighted by the editor and reviewer #3, including additional experimental controls, clarification of experimental repetition and statistics, and further revision of wording and claims. Accordingly, we have added negative controls for key IP experiments, benchmarking controls for mEGFR effects on STING signaling, and an additional rescue experiment for DDR-associated readouts, revised figure legends and associated annotations to improve transparency regarding reproducibility, and added a reproducibility statement. Furthermore, we toned down statements whose interpretations previously exceeded the data, particularly regarding DDR rescue and interpretation. Per the reviewer's advice, we have also removed the PLA data and Appendix Figure S7 and have adjusted the model figure and Discussion accordingly. We believe that the revision has now addressed the remaining concerns and is significantly improved. Detailed point-by-point responses are provided below.

Referee #3:

In their revised article 'Oncogenic EGFR rewires STING-TBK1 immune machinery by tyrosine phosphorylation to license DNA damage tolerance', Shen et al. provide extra evidence to support their hypothesis of mEGFR regulation of STING/TBK1. As before, whilst the data are very abundant, and the authors did a commendable amount of work, the manuscript still suffers from some important weaknesses, as outlined below. Where possible, I will give the authors the option to address the points by rephrasing aspects of the manuscript, or removing data, rather than performing extra experiments.

We thank the reviewer for the careful re-evaluation of our revised manuscript and for recognizing the additional evidence and substantial work included in the revision. We also appreciate the constructive guidance that several remaining concerns could be addressed by clarification, rephrasing, or removal of insufficiently supported data. In this final revision, we have closely followed this guidance by adding and clarifying key controls, improving the reporting of experimental repetition and quantification, removing data for which the current controls were insufficient, and further toning down the wording and interpretation throughout the manuscript. Detailed point-by-point responses are provided below.

Major points

1. I had previously requested quantifications of experiments, which the authors

interpreted as giving numerical values for individual experiments. What was also implied was that the authors should be more transparent about how often experiments were repeated, and how reproducible the results were. I cannot find any indication on the figures or figure legends for how often experiments were performed. Especially for experiments that are analyzed by WBs, and colony formation assays is this necessary. Please provide this information, and ideally also provide bar graphs showing quantifications from multiple experiments.

We agree that experimental reproducibility should be clearly stated. We have therefore added the number of independent repeats to the relevant figure legends and source data. Key immunoblot experiments, including EGFR-dependent STING/TBK1 phosphorylation, STING tyrosine phosphorylation, and DDR-associated readouts, were independently repeated with similar results. We also provide densitometric quantification for the principal immunoblot signals in the figures, expanded-view figures, or source data, as appropriate.

For colony-formation assays, the key experiments were independently repeated with similar trends; the number of independent repeats is now stated in the corresponding legends. We have added bar-graph quantification for the key clonogenic experiments **(Figure 5E-G**). Specifically, we now provide quantification for key conclusion-supporting immunoblots, bar-graph quantification for the colony-formation assays, and the number of independent experimental repeats for all immunoblotting experiments.

2. With regards to my previous Major Point 8 (lack of negative controls in IP experiments), the authors just performed one experiment with added controls (Fig. S8A, not referenced in the text). This is obviously not sufficient. Every IP experiment needs to have negative controls, otherwise their value cannot be assessed.

We agree with the reviewer that appropriate negative controls are essential for interpreting co-immunoprecipitation experiments. We apologize that, in the previous revision, these controls were not sufficiently visible or clearly described, and that the corresponding control dataset was not specifically referenced in the text. In the revised manuscript, we have ensured that each IP experiment is presented with, or specifically linked to, the appropriate negative control in the figure legends, Methods, and source data. For endogenous IP experiments using anti-EGFR, anti-TBK1, or anti-phosphotyrosine antibodies, matched normal IgG IPs are included as the relevant negative controls. For epitope-tagged reconstitution experiments, the corresponding bait-negative or tag-only conditions serve as specificity controls.

In addition, to further strengthen specificity in a key endogenous co-IP setting, we added cGAS and GAPDH immunoblotting as unrelated-protein controls to the endogenous EGFR IP experiment and moved this dataset from the appendix into the main figure **(Figure 2E)**. These controls show that EGFR IP does not nonspecifically recover unrelated proteins under these conditions, while preserving selective recovery

of STING and TBK1. We have also revised the text and model to describe these data as evidence for an association between EGFR and STING/TBK1-containing complexes, without implying that the current IP experiments establish direct biochemical interactions.

3. Regarding the immunofluorescence analysis of co-localisation (Fig. 2A-D; Fig. EV2AB): I had previously assumed that these images represent single confocal slices, but the authors wording in the revised manuscript (lines 200-201,'We reconstructed confocal z-stacks in three dimensions (3D) to exclude possible artefacts in 2D projections.') make it sound like projections were used. This is obviously not valid, and the two 3D reconstructed images shown in Fig. 2D are not sufficient to resolve this. Overall, however, I do not think that extra experiments are required. I would accept it if the authors changed all mentions of "colocalization" to 'the two proteins occupying similar intracellular areas, with a potential of partial co-localisation', as this describes the data more accurately.

We agree with the reviewer and have revised the manuscript throughout to avoid overstating the immunofluorescence data as definitive colocalization. As suggested, we have replaced "colocalization" with more cautious wording, such as "the two proteins occupying similar intracellular areas, with a potential for partial colocalization," or "spatial association," depending on context.

We have also clarified the description of the confocal analysis to avoid implying that 2D projections were used to establish colocalization. The 3D reconstruction is now described only as a supporting visualization to reduce possible artifacts from image projection, not as evidence for a definitive molecular interaction or strict colocalization.

4. Furthermore, with regards to co-localisation, the PLA experiments lack the fundamental controls in which PLA is performed with samples in which first one of the two antibodies, and then the other, has been replaced with IgG of the correct isotype/species. PLA is a notoriously artefact-prone procedure, and without this control, specificity cannot be assessed. I would, however, accept it if the authors removed the PLA data instead of adding extra experiments.

We agree with the reviewer that, without the full set of antibody-substitution controls, the specificity of the PLA experiments cannot be rigorously assessed. As the reviewer suggested, rather than adding new PLA control experiments at this stage or over-interpreting these data, we have removed the PLA data from the revised manuscript. We have also revised the related text, figure legends, and model accordingly, so that our conclusions no longer rely on the PLA results.

5. The wording and interpretations by the authors still is frequently somewhat disconnected to the actual data, and should be toned down. (a) In the abstract, lines

50-52, and the results section (lines 379-383), the appearance is given that TBK1 loss/inhibition has a dramatic and statistically significant effect. But in actuality, the data are more nuanced, with large variability (especially between organoids, Fig. 7AB, but also between cell lines, Fig. EV4L-O), and generally mild effects (max 2-3 fold sensitivity difference). Furthermore, given that DepMap does not show a generality of mEGFR cell lines being sensitive to TBK1 loss also indicates that caution is the appropriate way forward. (b) In the Discussion, lines 467-469, again the language seems to be too grand when the data from Fig. 3K-L are somewhat subtle. (c) In the Discussion (line 569), the authors describe their findings as a paradigm shift. Please remove this.

We agree that several statements in the previous version were stronger than warranted by the data's magnitude and variability. We have therefore further toned down the wording throughout the manuscript. In the Abstract and Results, we now describe the effect of TBK1 loss or inhibition on cisplatin sensitivity as moderate and context-dependent, rather than uniformly strong. In the Discussion, we revised the text for **Figs. 3K-L** to avoid overstating the effect of STING tyrosine phosphorylation on complex assembly. We have also removed the phrase "paradigm shift" and replaced it with more cautious language.

6. The benchmarking of mEGFR effects on STING dependent signalling (lines 139-142) need to include the condition of mEGFR and STING co-expression together with diABZI or mEGFR expression together with STING/cGAS co-expression. Also, this data cannot be buried in the supplement, as it is important to assess the actual impact of mEGFR (which appears to be consistent and therefore likely to be important despite appearing to be relatively mild - Fig. S1A).

We thank the reviewer for this helpful suggestion and agree that additional benchmarking is important to place the magnitude of the mEGFR effect in the context of canonical STING activation. In response, we added the requested comparison conditions in the HEK293T/5×ISRE-Luc reconstitution system, including STING + mEGFR + diABZI and STING + mEGFR + cGAS, and moved these data into the revised Expanded View figures **(Fig. EV1D-E)**, with corresponding reference in the main text.

These data show that mEGFR reproducibly enhances STING-dependent reporter activity relative to STING alone, but that this effect is moderate compared with canonical activation by diABZI or cGAS **(Fig. EV1D-E)**. When STING signaling is already strongly activated by diABZI treatment or cGAS co-expression, mEGFR does not further increase reporter output substantially. We therefore now interpret mEGFR as a consistent but moderate potentiator, rather than a substitute for canonical STING agonism or an amplifier of an already strongly activated STING state.

7. Whilst I appreciate that generally, HEK293 experiments are followed up with more relevant cell lines, there is still a set of data that is only performed in HEK293 cells,

relating to the more detailed analysis of the tyrosine mutants (Fig. 3H-L).

We agree that the detailed tyrosine-mutant analyses in Fig. 3H-L were performed in the HEK293T reconstitution system, rather than in endogenous NSCLC cells. These experiments were designed for controlled site-mapping and phosphomimetic or phospho-deficient comparisons, not to imply that all mutant-specific effects have been fully established in tumor cell settings.

We note that complementary endogenous data, including pY100-IP analyses in H1975 and H1299 cells, EGFR kinase inhibitor sensitivity, and downstream functional phenotypes, support the relevance of these candidate residues. Nevertheless, we have revised the Discussion to state this limitation more specifically and now interpret Fig. 3H-L more cautiously as mechanistic mapping in a reconstitution system.

8. The findings on TBK1 regulating DNA damage processing are very unexpected, and more evidence is required therefore. In my previous review, I had requested rescues of shRNA experiments, but the authors only partially did this (compare the assays in Fig. 6D-J with the single rescue experiment, Fig. EV4D). Furthermore, that single rescue experiment actually shows only subtle phenotypes of TBK1 loss in contrast to the experiments of the main figure). Therefore, the authors would either need to perform rescues of the other assays as well, or remove the DNA response aspect from the manuscript. Their claims on TBK1 impacting cisplatin would obviously have to be much more subtle in that case (which they should be in any case, see Major Point 5).

We thank the reviewer for this important point and agree that the DDR-related conclusion should be supported by rescue experiments and stated cautiously. In response, we performed an additional rescue experiment using shRNA-resistant TBK1-2YE and examined key DDR-associated readouts at 24 h and 48 h after cisplatin treatment **(Fig. EV4D)**. Re-expression of TBK1-2YE increased Rad51 and partially reduced the accumulation of γH2A.X and cleaved PARP observed upon TBK1 depletion, supporting a functional role for TBK1 in these DDR-associated phenotypes.

At the same time, we recognize that these rescue experiments do not fully define the underlying DNA-repair mechanism, nor do they establish that TBK1 directly controls DNA damage processing. We have therefore revised the Abstract, Results, Discussion, and model figure to remove or soften stronger mechanistic claims. Consistent with this more cautious interpretation, we have also revised the title from "Oncogenic EGFR rewires STING–TBK1 immune machinery by tyrosine phosphorylation to license DNA damage tolerance" to "Oncogenic EGFR rewires STING–TBK1 signaling to support DNA damage tolerance." We now describe TBK1 as supporting DDR-associated responses and cisplatin tolerance, rather than as directly regulating a fully resolved DNA-repair pathway.

9. The model figure must indicate that the interpretation of a direct complex between

TBK1, STING and mEGFR is no proven by the data (none of the experiments shows a direct interaction, all the interactions the authors observed could be from bridging proteins. Direct interaction could only be inferred by recombinantly expressed and biochemically purified proteins.) At the very least the authors need to add something like a question mark next to the words 'complex formation'. In light of this, the words in the Discussion (lines 458-459) "integrate into STING signalosomes" should also be replaced with "associate with STING".

We agree with the reviewer and have revised both the model figure and the Discussion accordingly. In the model figure, we added a question mark next to "complex formation" to indicate that a direct ternary complex among mEGFR, STING, and TBK1 has not been established by the current data. We have also revised the text to avoid implying a direct biochemical interaction and replaced stronger wording, such as "integrate into STING signalosomes," with the more cautious phrase "associate with STING".

10. Fig. S7 should be removed. Fig. S7A seems to be misleading, as only a few selected patient samples from over a 1000 in total are shown. Fig. S7BC do not show data stratified by mEGFR expression, and the impact of TBK1 expression could therefore be mediated by completely different mechanisms than the ones the authors are studying.

We agree that Fig. S7, in its previous form, was not sufficiently informative for the mechanistic question addressed in this study and could lead to overinterpretation. We have therefore removed Fig. S7 from the revised manuscript and adjusted the related text accordingly.

Minor point

1. The wording of line 446-448 makes it seem like there is something special about breast cancer with regards to cGAS-STING, but this has not been established. It just happened that the studies the authors cite used breast cancer cell lines. But they did not show that the effect is limited or generally applicable to breast cancer.

We agree that the previous wording could incorrectly imply a breast cancer-specific feature of cGAS-STING signaling. We have revised the corresponding Discussion text to remove this implication and now cite these studies more generally as examples of context-dependent cGAS-STING signaling in cancer.

## 2nd Revision - Editorial Decision 1st Jul 2026

Dear Dr Xu,

Thank you for submitting the revised version of your manuscript. I have now evaluated your amended manuscript and concluded that the remaining minor concerns have been sufficiently addressed.

I am thus pleased to inform you that your manuscript has been accepted for publication in the EMBO Journal.

Your manuscript will be processed for publication by EMBO Press. It will be copy edited and you will receive page proofs prior to publication. Please note that you will be contacted by Springer Nature Author Services to complete licensing and payment information.

Please note that it is The EMBO Journal policy for the transcript of the editorial process (containing referee reports and your response letter) to be published as an online supplement to each paper. If you do NOT want this, you will need to inform the Editorial Office via email immediately. More information is available here: https://www.embopress.org/transparent-process#Review_Process

You may qualify for financial assistance for your publication charges - either via a Springer Nature fully open access agreement or an EMBO initiative. Check your eligibility: https://www.embopress.org/page/journal/14602075/authorguide#chargesguide

Should you be planning a Press Release on your article, please notify the production team via this form in order to coordinate publication and release dates: https://forms.cloud.microsoft/e/eH4A8JVP6g

If you have any questions, please do not hesitate to contact the Editorial Office. Thank you for your contribution to The EMBO Journal.

Thank you again for this contribution to The EMBO Journal and congratulations on a successful publication! Please consider us again in the future for your most exciting work.

Best regards,

Daniel Klimmeck

Daniel Klimmeck, PhD
Senior Editor
The EMBO Journal
EMBO
Postfach 1022-40
Meyerhofstrasse 1
D-69117 Heidelberg
contact@embojournal.org

*****

Please note that it is The EMBO Journal policy for the transcript of the editorial process (containing referee reports and your response letters) to be published as an online supplement to each paper. If you should prefer removal of any referee-only figures included in the point-by-point response(s), e.g. because they may still be used for future publication or because they have been reproduced from published work by others, please do let us know immediately via response email.
More information is available here: https://link.springer.com/partners/embo-press/editorial-policies#Peer%20review

## EMBO Press Author Checklist

| | |
|---|---|
| Corresponding Author Name: Pinglong Xu | |
| Journal Submitted to: The EMBO Journal | |
| Manuscript Number: EMBOJ-2025-122789 | |

**USEFUL LINKS FOR COMPLETING THIS FORM**
The EMBO Journal - Author Guidelines
EMBO Reports - Author Guidelines
Molecular Systems Biology - Author Guidelines
EMBO Molecular Medicine - Author Guidelines

**Reporting Checklist for Life Science Articles (updated January**
This checklist is adapted from Materials Design Analysis Reporting (MDAR) Checklist for Authors. MDAR establishes a minimum set of requirements in transparent reporting in the life sciences (see Statement of Task: 10.31222/osf.io/9sm4x). Please follow the journal's guidelines in preparing your
**Please note that a copy of this checklist will be published alongside your article.**

**Abridged guidelines for figures**
**1. Data**
The data shown in figures should satisfy the following conditions:
- the data were obtained and processed according to the field's best practice and are presented to reflect the results of the experiments in an accurate and unbiased manner.
- ideally, figure panels should include only measurements that are directly comparable to each other and obtained with the same assay.
- plots include clearly labeled error bars for independent experiments and sample sizes. Unless justified, error bars should not be shown for technical
- if n<5, the individual data points from each experiment should be plotted. Any statistical test employed should be justified.
- Source Data should be included to report the data underlying figures according to the guidelines set out in the authorship guidelines on Data

**2. Captions**
Each figure caption should contain the following information, for each panel where they are relevant:
- a specification of the experimental system investigated (eg cell line, species name).
- the assay(s) and method(s) used to carry out the reported observations and measurements.
- an explicit mention of the biological and chemical entity(ies) that are being measured.
- an explicit mention of the biological and chemical entity(ies) that are altered/varied/perturbed in a controlled manner.
- the exact sample size (n) for each experimental group/condition, given as a number, not a range;
- a description of the sample collection allowing the reader to understand whether the samples represent technical or biological replicates (including how many animals, litters, cultures, etc.).
- a statement of how many times the experiment shown was independently replicated in the laboratory.
- definitions of statistical methods and measures:
  - common tests, such as t-test (please specify whether paired vs. unpaired), simple χ2 tests, Wilcoxon and Mann-Whitney tests, can be unambiguously identified by name only, but more complex techniques should be described in the methods section;
  - are tests one-sided or two-sided?
  - are there adjustments for multiple comparisons?
  - exact statistical test results, e.g., P values = x but not P values < x;
  - definition of 'center values' as median or average;
  - definition of error bars as s.d. or s.e.m.

**Please complete ALL of the questions below.**
**Select "Not Applicable" only when the requested information is not relevant for your study.**

**Materials**

| **Newly Created Materials** | **Information included in the manuscript?** | **In which section is the information available?** (Reagents and Tools Table, Materials and Methods, Figures, Data Availability Section) |
|---|---|---|
| New materials and reagents need to be available; do any restrictions apply? | Not Applicable | |

| **Antibodies** | **Information included in the manuscript?** | **In which section is the information available?** (Reagents and Tools Table, Materials and Methods, Figures, Data Availability Section) |
|---|---|---|
| For **antibodies** provide the following information:<br>- Commercial antibodies: RRID (if possible) or supplier name, catalogue number and or/clone number<br>- Non-commercial: RRID or citation | Yes | Reagents and Tools Table |

| **DNA and RNA sequences** | **Information included in the manuscript?** | **In which section is the information available?** (Reagents and Tools Table, Materials and Methods, Figures, Data Availability Section) |
|---|---|---|
| **Short novel DNA or RNA including primers, probes**: provide the sequences. | Yes | Reagents and Tools Table |

| **Cell materials** | **Information included in the manuscript?** | **In which section is the information available?** (Reagents and Tools Table, Materials and Methods, Figures, Data Availability Section) |
|---|---|---|
| **Cell lines:** Provide species information, strain. Provide accession number in repository **OR** supplier name, catalog number, clone number, and/**OR** RRID. | Yes | Reagents and Tools Table; Materials and Methods |
| **Primary cultures:** Provide species, strain, sex of origin, genetic modification status. | Yes | Materials and Methods |
| Report if the cell lines were recently **authenticated** (e.g., by STR profiling) and tested for mycoplasma contamination. | Yes | Materials and Methods |

| **Experimental animals** | **Information included in the manuscript?** | **In which section is the information available?** (Reagents and Tools Table, Materials and Methods, Figures, Data Availability Section) |
|---|---|---|
| **Laboratory animals or Model organisms:** Provide species, strain, sex, age, genetic modification status. Provide accession number in repository **OR** supplier name, catalog number, clone number, **OR** RRID. | Yes | Reagents and Tools Table; Materials and Methods |
| **Animal observed in or captured from the field:** Provide species, sex, and age where possible. | Not Applicable | |
| Please detail **housing and husbandry conditions**. | Yes | Materials and Methods |

| **Plants and microbes** | **Information included in the manuscript?** | **In which section is the information available?** (Reagents and Tools Table, Materials and Methods, Figures, Data Availability Section) |
|---|---|---|
| **Plants:** provide species and strain, ecotype and cultivar where relevant, unique accession number if available, and source (including location for collected wild specimens). | Not Applicable | |
| **Microbes:** provide species and strain, unique accession number if available, and source. | Not Applicable | |

| **Human research participants** | **Information included in the manuscript?** | **In which section is the information available?** (Reagents and Tools Table, Materials and Methods, Figures, Data Availability Section) |
|---|---|---|
| If collected and within the bounds of privacy constraints report on age, sex and gender or ethnicity for all study participants. | Yes | Materials and Methods |

| **Core facilities** | **Information included in the manuscript?** | **In which section is the information available?** (Reagents and Tools Table, Materials and Methods, Figures, Data Availability Section) |
|---|---|---|
| If your work benefited from core facilities, was their service mentioned in the acknowledgments section? | Yes | Acknowledgements |

**Design**

| Study protocol | Information included in the manuscript? | In which section is the information available? (Reagents and Tools Table, Materials and Methods, Figures, Data Availability Section) |
|---|---|---|
| If study protocol has been **pre-registered, provide DOI in the manuscript**. For clinical trials, provide the trial registration number **OR** cite DOI. | Not Applicable | |
| Report the **clinical trial registration number** (at ClinicalTrials.gov or equivalent), where applicable. | Not Applicable | |

| Laboratory protocol | Information included in the manuscript? | In which section is the information available? (Reagents and Tools Table, Materials and Methods, Figures, Data Availability Section) |
|---|---|---|
| Provide DOI OR other citation details if **external detailed step-by-step protocols** are available. | Not Applicable | |

| Experimental study design and statistics | Information included in the manuscript? | In which section is the information available? (Reagents and Tools Table, Materials and Methods, Figures, Data Availability Section) |
|---|---|---|
| Include a statement about **sample size** estimate even if no statistical methods were used. | Yes | Materials and Methods |
| Were any steps taken to minimize the effects of subjective bias when allocating animals/samples to treatment (e.g. **randomization procedure**)? If yes, have they been described? | Yes | Materials and Methods |
| Include a statement about **blinding** even if no blinding was done. | Yes | Materials and Methods |
| Describe **inclusion/exclusion criteria** if samples or animals were excluded from the analysis. Were the criteria pre-established?<br><br>If sample or data points were omitted from analysis, report if this was due to attrition or intentional exclusion and provide justification. | Yes | Materials and Methods |
| For every figure, are **statistical tests** justified as appropriate? Do the data meet the assumptions of the tests (e.g., normal distribution)? Describe any methods used to assess it. Is there an estimate of variation within each group of data? Is the variance similar between the groups that are being statistically compared? | Yes | Materials and Methods |

| Sample definition and in-laboratory replication | Information included in the manuscript? | In which section is the information available? (Reagents and Tools Table, Materials and Methods, Figures, Data Availability Section) |
|---|---|---|
| In the figure legends: state number of times the experiment was **replicated** in laboratory. | Yes | Figure legends; Data Availability Section |
| In the figure legends: define whether data describe **technical or biological replicates**. | Yes | Figure legends; Data Availability Section |

## Ethics

| Ethics | Information included in the manuscript? | In which section is the information available? (Reagents and Tools Table, Materials and Methods, Figures, Data Availability Section) |
|---|---|---|
| Studies involving **human participants**: State details of **authority granting ethics approval** (IRB or equivalent committee(s), provide reference number for approval. | Yes | Materials and Methods |
| Studies involving **human participants**: Include a statement confirming that **informed consent** was obtained from all subjects and that the experiments conformed to the principles set out in the WMA Declaration of Helsinki and the Department of Health and Human Services Belmont Report. | Yes | Materials and Methods |
| Studies involving **human participants:** For publication of **patient photos**, include a statement confirming that consent to publish was obtained. | Not Applicable | |
| Studies involving experimental **animals**: State details of **authority granting ethics approval** (IRB or equivalent committee(s), provide reference number for approval. Include a statement of compliance with ethical regulations. | Yes | Materials and Methods |
| Studies involving **specimen and field samples:** State if relevant **permits** obtained, provide details of authority approving study; if none were required, explain why. | Not Applicable | |

| Dual Use Research of Concern (DURC) | Information included in the manuscript? | In which section is the information available? (Reagents and Tools Table, Materials and Methods, Figures, Data Availability Section) |
|---|---|---|
| Could your study fall under dual use research restrictions? Please check biosecurity documents and list of **select agents and toxins** (CDC): https://www.selectagents.gov/sat/list.htm | Not Applicable | |
| If you used a select agent, is the security level of the lab appropriate and reported in the manuscript? | Not Applicable | |
| If a study is subject to dual use research of concern regulations, is the name of the **authority granting approval and reference number** for the regulatory approval provided in the manuscript? | Not Applicable | |

## Reporting

The MDAR framework recommends adoption of discipline-specific guidelines, established and endorsed through community initiatives. Journals have their own policy about requiring specific guidelines and recommendations to complement MDAR.

| Adherence to community standards | Information included in the manuscript? | In which section is the information available? (Reagents and Tools Table, Materials and Methods, Figures, Data Availability Section) |
|---|---|---|
| State if relevant guidelines or checklists (e.g., **ICMJE, MIBBI, ARRIVE, PRISMA**) have been followed or provided. | Not Applicable | |
| For **tumor marker prognostic studies,** we recommend that you follow the **REMARK** reporting guidelines (see link list at top right). See author guidelines, under 'Reporting Guidelines'. Please confirm you have followed these guidelines. | Not Applicable | |
| For **phase II and III randomized controlled trials**, please refer to the **CONSORT** flow diagram (see link list at top right) and submit the CONSORT checklist (see link list at top right) with your submission. See author guidelines, under 'Reporting Guidelines'. Please confirm you have submitted this list. | Not Applicable | |

## Data Availability

| Data availability | Information included in the manuscript? | In which section is the information available? (Reagents and Tools Table, Materials and Methods, Figures, Data Availability Section) |
|---|---|---|
| Have **primary datasets** been deposited according to the journal's guidelines (see 'Data Deposition' section) and the respective accession numbers provided in the Data Availability Section? | Yes | Data Availability Section |
| Were **human clinical and genomic datasets** deposited in a public access-controlled repository in accordance to ethical obligations to the patients and to the applicable consent agreement? | Not Applicable | |
| Are **computational models** that are central and integral to a study available without restrictions in a machine-readable form? Were the relevant accession numbers or links provided? | Not Applicable | |
| If publicly available data were reused, provide the respective **data citations in the reference list**. | Not Applicable | |
