# Supplementary material for: Oncogenic EGFR rewires STING-TBK1 signalosomes to license DNA damage tolerance in NSCLC
Source: EMBO J. 2026 Jul 10;45(16):5759–92. doi: 10.1038/s44318-026-00856-3 (PMC13482819; doi:10.1038/s44318-026-00856-3)

# Source Data_Appendix Figures_Blots

## Appendix Fig. S1A

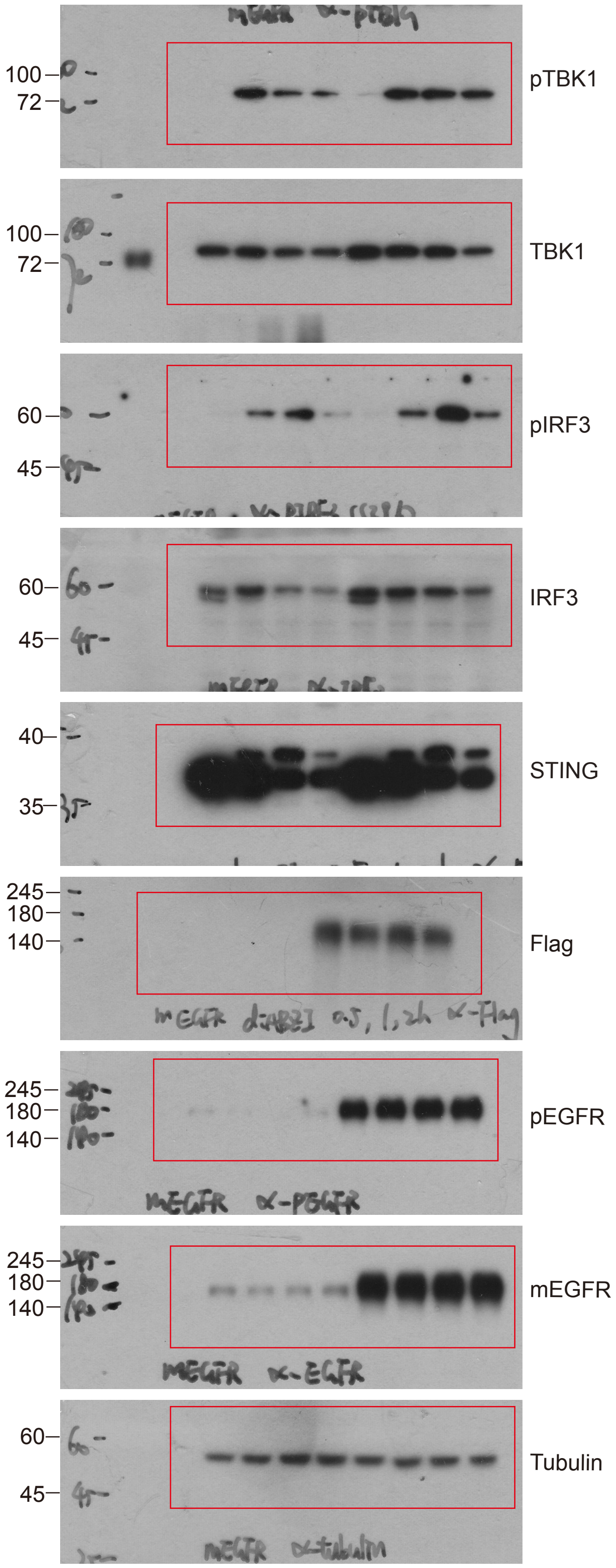


## Appendix Fig. S1B

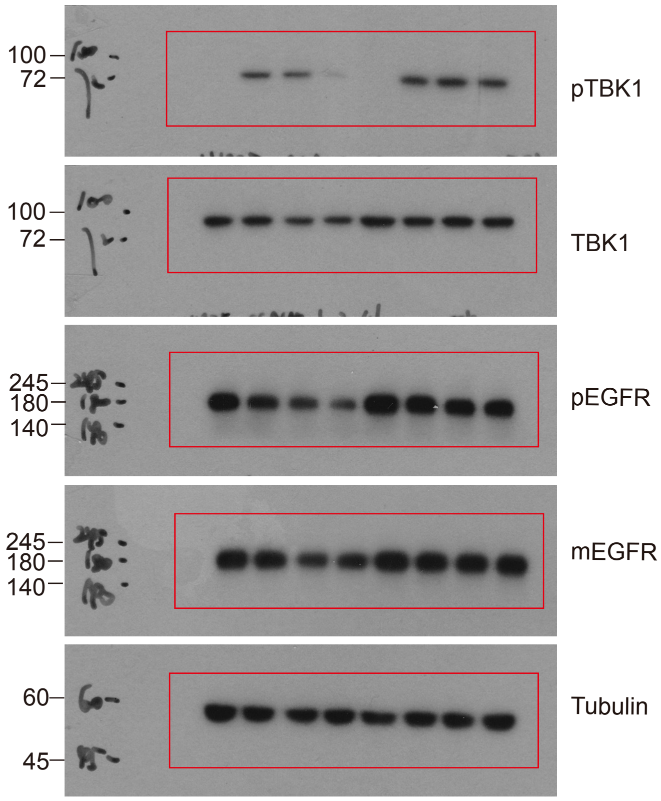

## Appendix Fig. S2A

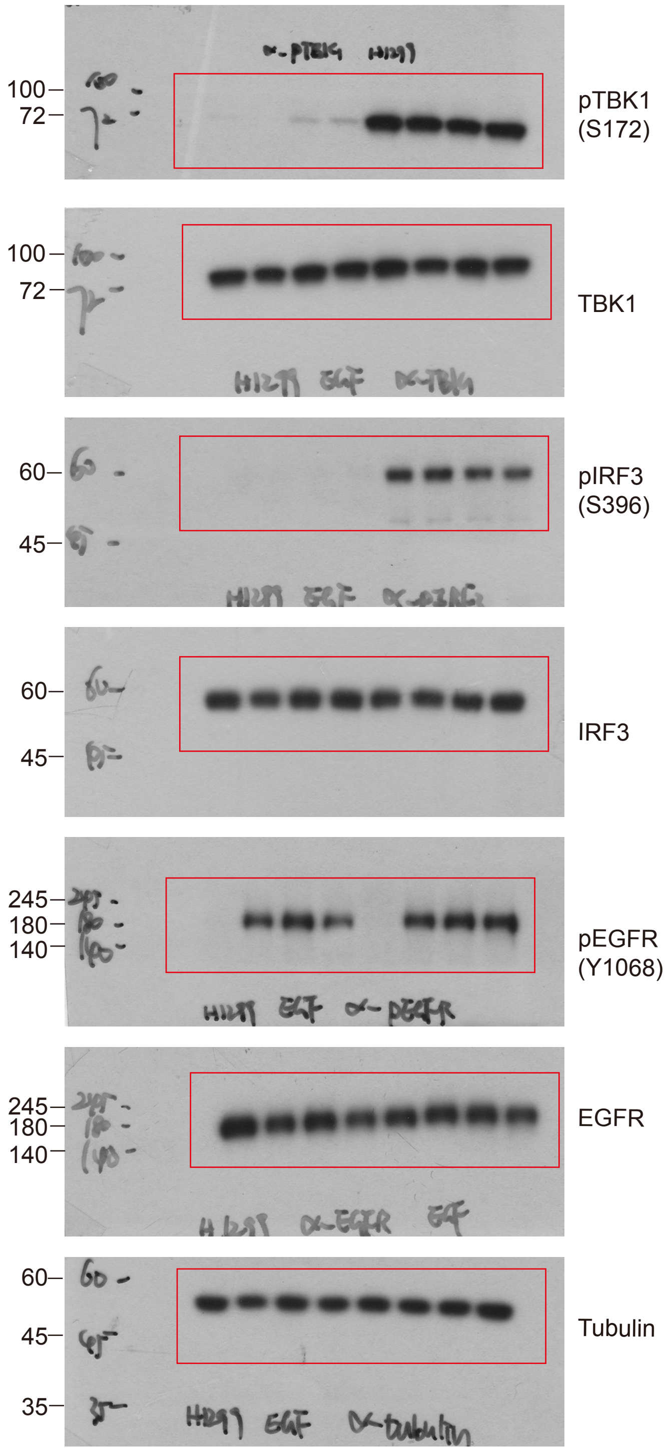


## Appendix Fig. S2B

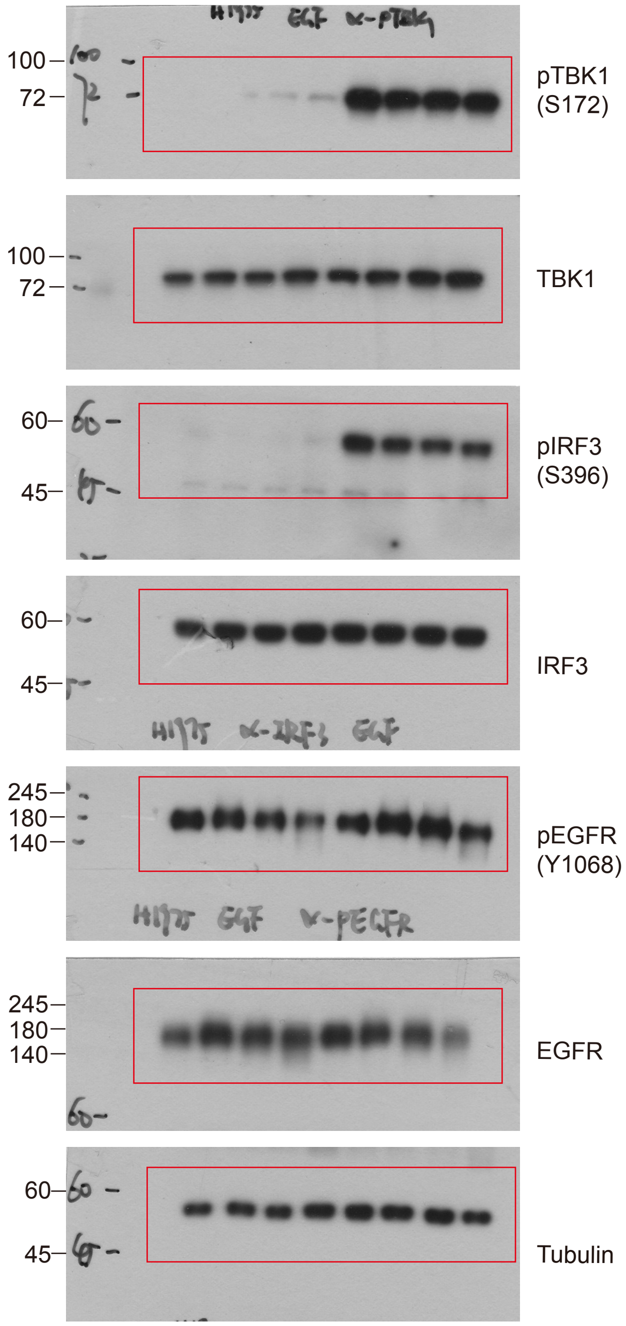

Supplement: Supplementary file 9 [file 44318_2026_856_MOESM15_ESM.zip › Appendix_Source data/Source Data_Appendix Figures_Blots.pdf]
