# Supplementary material for: Oncogenic EGFR rewires STING-TBK1 signalosomes to license DNA damage tolerance in NSCLC
Source: EMBO J. 2026 Jul 10;45(16):5759–92. doi: 10.1038/s44318-026-00856-3 (PMC13482819; doi:10.1038/s44318-026-00856-3)
Supplement: Supplementary file 14 [file 44318_2026_856_MOESM1_ESM.zip › Movie EV1/Movie EV1 README.rtfd/TXT.rtf]

Title: Movie EV1. Three-dimensional reconstruction of STING and TBK1 localization in H1975 cells expressing mEGFR
Description: This folder contains the video files corresponding to Movie EV1, showing representative three-dimensional reconstructions of immunofluorescence images acquired from H1975 cells expressing mEGFR under the indicated treatment conditions. The movies were generated from z-stack confocal images and are provided to facilitate visualization of the subcellular localization patterns of STING and TBK1 in relation to mEGFR.
Included files:
	1	H1975 Ctrl STING+mEGFR.mp4 Three-dimensional reconstruction showing STING and mEGFR localization in control-treated H1975 cells.
	2	H1975 Ctrl TBK1+mEGFR.mp4 Three-dimensional reconstruction showing TBK1 and mEGFR localization in control-treated H1975 cells.
	3	H1975 diABZI STING+mEGFR.mp4 Three-dimensional reconstruction showing STING and mEGFR localization in diABZI-treated H1975 cells.
	4	H1975 diABZI TBK1+mEGFR.mp4 Three-dimensional reconstruction showing TBK1 and mEGFR localization in diABZI-treated H1975 cells.
